# Profilin-1 regulates DNA replication forks in a context-dependent fashion by interacting with SNF2H and BOD1L

**DOI:** 10.1038/s41467-022-34310-9

**Published:** 2022-11-01

**Authors:** Cuige Zhu, Mari Iwase, Ziqian Li, Faliang Wang, Annabel Quinet, Alessandro Vindigni, Jieya Shao

**Affiliations:** 1 Divison of Oncology, Department of Medicine, Washington University School of Medicine, St. Louis, MO USA; 2 Department of Microbial and Biochemical Pharmacy, School of Pharmaceutical Sciences, Sun Yat-sen University, Guangzhou, China; 3 UMR Genetic Stability Stem Cells and Radiation, University of Paris and University of Paris-Saclay, INSERM, iRCM/IBFJ CEA, Fontenay-aux-Roses, France; 4 Siteman Cancer Center, Washington University School of Medicine, St. Louis, MO 63110 USA

**Keywords:** Stalled forks, Cancer therapy

## Abstract

DNA replication forks are tightly controlled by a large protein network consisting of well-known core regulators and many accessory factors which remain functionally undefined. In this study, we report previously unknown nuclear functions of the actin-binding factor profilin-1 (PFN1) in DNA replication, which occur in a context-dependent fashion and require its binding to poly-L-proline (PLP)-containing proteins instead of actin. In unperturbed cells, PFN1 increases DNA replication initiation and accelerates fork progression by binding and stimulating the PLP-containing nucleosome remodeler SNF2H. Under replication stress, PFN1/SNF2H increases fork stalling and functionally collaborates with fork reversal enzymes to enable the over-resection of unprotected forks. In addition, PFN1 binds and functionally attenuates the PLP-containing fork protector BODL1 to increase the resection of a subset of stressed forks. Accordingly, raising nuclear PFN1 level decreases genome stability and cell survival during replication stress. Thus, PFN1 is a multi-functional regulator of DNA replication with exploitable anticancer potential.

## Abstract (web summary)

Subcellular localization plays an important yet underappreciated role in protein functions. Here the authors defined novel and context-dependent nuclear functions of the actin-binding factor profilin-1 in DNA replication fork dynamics and stability.

## Introduction

DNA replication is one of the most complex and tightly regulated cellular processes by virtue of its essential role in biological inheritance of all living organisms. This has become increasingly evident since the development of high-throughput approaches, most notably iPOND (Isolation of Proteins on Nascent DNA) assay, which discovered hundreds of proteins physically present at the replication forks^1–4^. Although the functional importance of most of these proteins for DNA replication are currently unknown, in-depth characterization of a few of them offered intriguing new insights into the complex regulatory mechanisms of DNA replication under normal and stressed conditions^5–9^. Since DNA replication defects cause genome instability, dissecting the mechanisms naturally governing DNA replication fork plasticity and stability may uncover therapeutically relevant clues for developing effective anticancer treatments.

DNA replication forks undergo frequent reversal during replication stress^10–14^. As an adaptive response, fork reversal allows replication forks to cope with DNA lesions by reversing their course^11,15^. Several DNA translocases and helicases including SMARCAL1, HLTF, ZRANB3, and FBH1 catalyze the reversal of model DNA fork structures in vitro and their loss of function in cells reduces the formation of reversed forks visualized by electron microscopy^11,16–22^. However, since in vivo fork reversal occurs in the context of chromatin and nascent DNA is densely chromatinized^23^, it remains unclear whether the DNA remodeling activities of the currently known enzymes are sufficient for fork reversal or their functional cooperation with other chromatin remodeling enzymes is required.

Though serving to mitigate replication stress, fork reversal bears the inherent risk of causing genomic instability due to the generation of one-ended double-stranded DNA breaks. A number of proteins have been found to protect the reversed nascent DNA against deleterious over-resection by DNA nucleases such as MRE11, DNA2, and EXO1^6,19,24–27^. While some of these fork protectors have well-known DNA repair functions such as BRCA1/2, FANCD2, and RIF1^9,19,24,26–31^, others like ABRO1 and VHL were not functionally connected to genome maintenance prior to the discovery of their fork-protective effects^32,33^. Of particular note, recent studies identified BOD1L, a previously uncharacterized large nuclear protein, as an essential fork-protective factor during replication stress by physically and functionally interacting with histone methyltransferase SETD1A and increasing histone H3 lysine 4 (H3K4) methylation needed for FANCD2-dependent nucleosome remodeling^6,34^. Despite the apparent differences in their mechanisms of action, these proteins protect stressed forks either by stabilizing RAD51 nucleofilaments on nascent DNA or controlling the access or activities of DNA resecting nucleases^15^. Interestingly, it was recently reported that at least two fork remodeling pathways generate distinct types of fork intermediates that are selectively stabilized by different fork-protective proteins. Specifically, fork remodeling by the SNF2-family DNA translocases SMARCAL1, ZRANB3, and HLTF triggers excessive nascent DNA degradation in the absence of BRCA2, FANCD2, and ABRO1, while forks remodeled by FBH1 are specifically degraded in the absence of BOD1L, VHL, and several other FA proteins^19,35^. Thus, the complexity of fork-regulatory mechanisms is due not only to the vast number of regulators but also their intricate functional relationships.

Despite our expanding knowledge of the core players in replication fork maintenance, our understanding of how they are functionally modulated by accessory factors remains relatively limited. Several findings including the inhibitory phosphorylation of SMARCAL1 by ATR kinase^36^ and the functional limitation of RAD51 by the single-stranded DNA binding protein RADX^8,37,38^ exemplified the tight functional control of the core fork regulators, but the molecular events regulating many of them are currently unknown. In this study, we report previously unrecognized nuclear functions of the small actin-binding factor profilin-1 (PFN1) as a regulator of DNA replication under both normal and stressed conditions. Identified more than four decades ago, the actin-binding protein PFN1 was extensively characterized for its cytoplasmic function in actin polymerization and dynamics^39^. However, its nuclear functions remained completely unknown until our recent discovery that it physically interacts with the poly-L-proline (PLP)-containing ENL protein in the Super Elongation Complex (SEC) and functionally inhibits the transcriptional elongation of various pro-cancer genes^40^. Not only did our prior finding reconcile the long-standing paradox of PFN1 as both an essential protein and a tumor suppressor, it also implicated other yet-unknown activities of nuclear PFN1 due to its ability to bind PLPs that are present in different proteins^41,42^. In this work, by leveraging existing iPOND/MS and PFN1 interactome data, we uncover context-dependent roles of nuclear PFN1 in regulating DNA replication forks under normal and stressed conditions via its physical and functional interactions with the PLP-containing nucleosome remodeling enzyme SNF2H and fork-protective factor BOD1L.

## Results

### PFN1 is important for unperturbed DNA replication

**Fig. 1:**
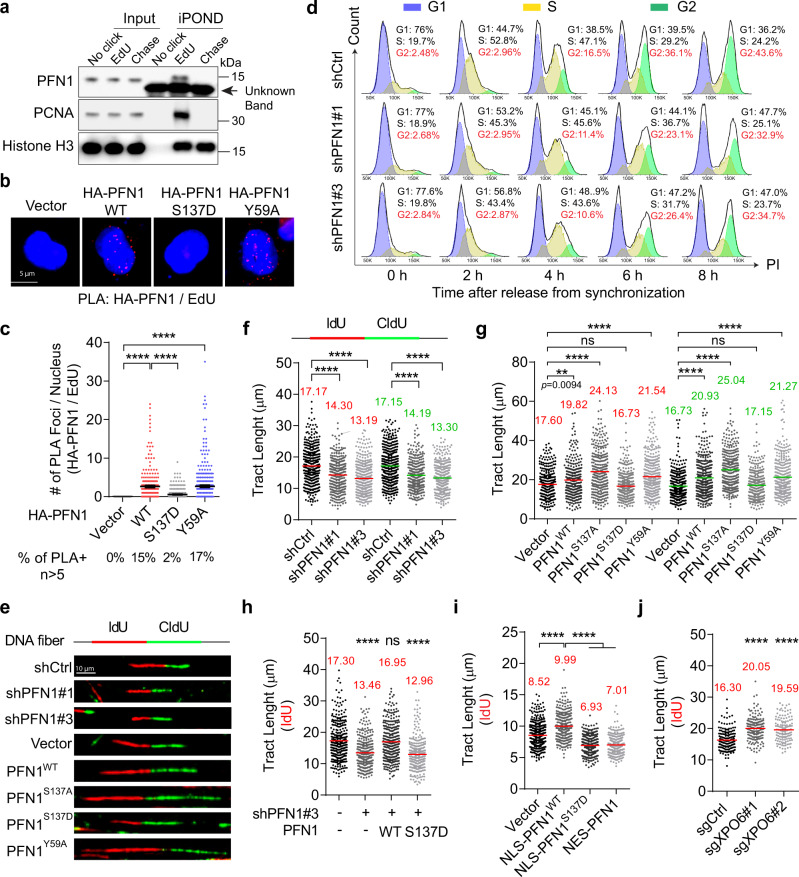
PFN1 is important for unperturbed DNA replication. **a** PFN1 is detected on nascent DNA by iPOND assay. HEK293T cells were labeled or not with EdU (10 µM) for 25 min, followed or not by thymidine (10 µM) chase for 1 h. **b**, **c** PLA between HA-PFN1 and biotinylated EdU in transfected HeLa cells. Scale bar, 5 µm. Quantification (in **c**) represents PLA foci numbers per nucleus out of at least 250 cells per condition. Error bars are mean ± SEM. Percentages of PLA-positive cells (>5 foci per nucleus) are shown on the bottom. **d** Loss of PFN1 delays S phase progression. MCF-10A cells infected with the indicated shRNAs were synchronized by double thymidine and released for the indicated times. The percentages of cells in G2 phase are colored in red. Samples were stained with propidium iodide and analyzed by flow cytometry and FlowJo. Gating strategies are provided in Supplementary Fig. 10. **e**–**g** Representative images (**e**) and tract length analysis (**f**, **g**) of single-molecule DNA fiber assay in MCF-10A cells expressing PFN1 shRNAs or cDNAs. Cells were labeled with 25 μM IdU (1st) and 250 μM CIdU (2nd) for 30 min each. Scale bar, 10 µm. **h** DNA fiber analysis in PFN1 knockdown MCF-10A cells functionally rescued by WT or S137D mutant form of PFN1. **i** DNA fiber analysis in MCF-7 cells expressing YFP or YFP-PFN1 tagged with NLS or NES. **j** DNA fiber analysis in control or XPO6 knockout MCF-10A cells. For (**f**–**j**), at least 300 DNA fibers were analyzed for each experimental condition, and mean values of tract lengths are shown. All DNA fiber and PLA data were analyzed using the Kruskal–Wallis test with Dunnett’s multiple comparisons. **p < 0.01; ****p < 0.0001; ns, not significant. Results were confirmed by at least two independent experiments. Source data are provided as a Source Data file.

In search of additional nuclear functions of PFN1, we found in a published iPOND/MS study that PFN1, but not other profilin isoforms or actin itself, interacts with newly synthesized but not mature DNA in four different cell lines^4^. Our iPOND/WB experiments confirmed that endogenous PFN1, but not actin, binds nascent but not mature DNA in HEK293T cells (Fig. 1a and Supplementary Fig. 1a). This was further validated by EdU-Click-coupled proximity ligation assay (PLA)^19,43^ using HA-PFN1-transfected HeLa cells (Fig. 1b, c). Interestingly, a separation-of-function mutation S137D, which specifically blocks PFN1 binding to poly-L-proline (PLP)-containing proteins but not actin^40,44,45^, abolished HA-PFN1 interaction with nascent DNA. In contrast, the Y59A mutation, which abolishes actin-binding but not PLP-binding of PFN1^45,46^, did not affect the interaction between PFN1 and nascent DNA. Next, we asked if PFN1 functionally regulates DNA replication. We used two shRNAs to silence PFN1 in the untransformed human mammary epithelial MCF-10A cells, synchronized them in late G1 phase by double thymidine block, and subsequently released them into S phase. By collecting released cells at different time points and performing DNA content analysis by flow cytometry, we discovered that cells with PFN1 knockdown progressed through S phase at a slower rate than control cells, and their G2/M entry was consequently delayed (Fig. 1d). This suggested that PFN1 may be important for DNA synthesis, the lack of which delays S phase completion.

To test the role of PFN1 in DNA replication, we next performed single-molecule DNA fiber assay by pulsing cells with IdU and CIdU sequentially^47^. By quantifying the tract lengths of dual-colored fibers, we found that PFN1 knockdown significantly decreased DNA replication speed in multiple human cell lines including MCF-10A, HEK293T, HeLa and breast cancer MCF-7 and MDA-MB-231 (Fig. 1e, f, Supplementary Fig. 1b and Supplementary Fig. 1c). This was also confirmed in a PFN1-null mouse chondrocyte cell line^40,48^ whose DNA fiber lengths were significantly shorter than those in wild type mouse chondrocytes or upon reconstitution with human PFN1 (Supplementary Fig. 1d and Supplementary Fig. 1e). Conversely, overexpressing PFN1 in MCF-10A and MCF-7 cells significantly increased fork speed (Fig. 1e, g, Supplementary Fig. 1f and Supplementary Fig. 1g). Notably, the actin-binding-deficient PFN1(Y59A) mutant^45,46^ was equally active as wild type PFN1 in promoting DNA replication. In contrast, the PLP-binding-deficient mutant PFN1(S137D)^44,45^ was completely inactive. Since S137D abolishes PLP-binding by mimicking Ser^137^ phosphorylation, we also examined the effect of the phospho-resistant PFN1(S137A) mutant. Consistent with its constitutive ability to bind PLPs, PFN1(S137A) promoted DNA replication to a higher degree than PFN1(WT) (Fig. 1g and Supplementary Fig. 1f). The same separation-of-function effects were also observed in the PFN1-null mouse chondrocytes where WT vs. mutant PFN1 were re-expressed (Supplementary Fig. 1d, e). Furthermore, PFN1 knockdown and rescue in MCF-10A cells confirmed the loss-of-function of S137D (Fig. 1h and Supplementary Fig. 1h). Collectively, our data suggested that PFN1 is important for DNA replication in a PLP-dependent manner.

Since PFN1 is present throughout the cell, we next tested whether its effect on DNA replication directly stems from its nuclear function. By expressing previously characterized PFN1 constructs fused with a nuclear localization sequence (NLS) or nuclear export sequence (NES)^40,44^, we observed that DNA replication was promoted by nuclear but not cytoplasmic PFN1 and the effect of NLS-PFN1 was abolished by S137D mutation (Fig. 1i). To confirm that the pro-replication activity of nuclear PFN1 can be observed on the endogenous level, we exploited the fact that its nuclear export is tightly regulated by exportin-6 (XPO6). XPO6 is a highly specific nuclear exporter of which the only known cargo thus far is the PFN1/actin complex, and prior studies from us and others showed that XPO6 loss causes nuclear retention of both PFN1 and actin^40,49^. Indeed, XPO6 knockdown or knockout increased DNA fiber tract lengths in MCF-10A and MCF-7 cells (Fig. 1j, Supplementary Fig. 1i, and Supplementary Fig. 1j), supporting the positive regulation of DNA replication by nuclear PFN1. Importantly, XPO6 knockdown in the PFN1-null mouse chondrocyte cells could not trigger a biologically (though statistically) significant increase in DNA fiber tract length until after PFN1 was re-expressed (Supplementary Fig. 1k, l). Collectively, our data uncovered a previously unknown role of nuclear PFN1 in the regulation of DNA replication fork speed, which depends on its interaction with PLP-containing proteins.

### PFN1 and SNF2H function together to promote DNA replication

**Fig. 2:**
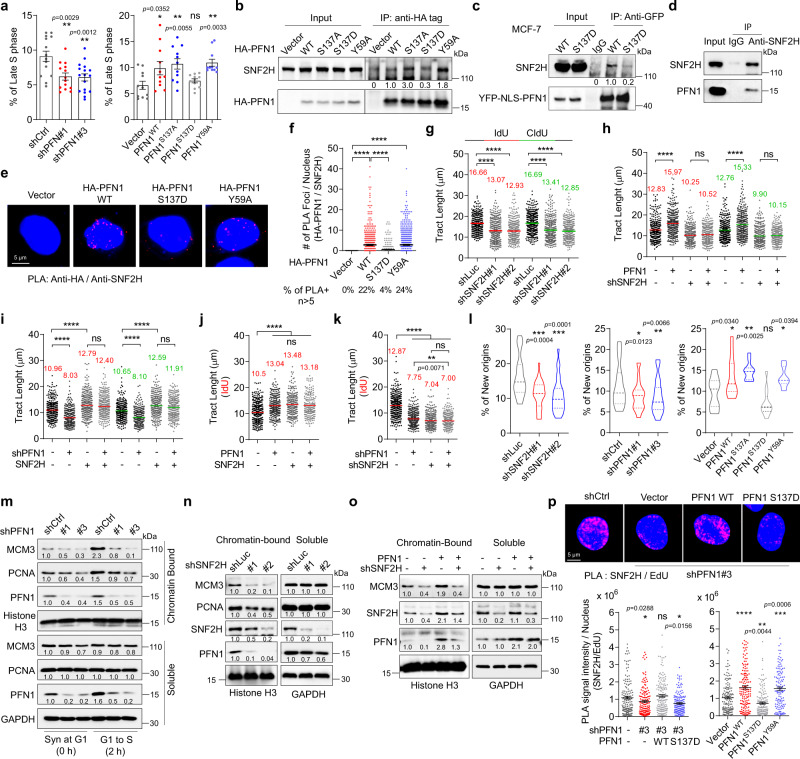
PFN1 and SNF2H function together to promote DNA replication. **a** Percentages of EdU-labeled (10 μM, 25 min) PFN1 knockdown and overexpressing MCF-10A cells in late S-phase. Data are mean ± SEM of *n* = 3 independent experiments containing about 3000 cells per group. *P*-values were based on One-Way ANOVA and Dunnett’s multiple comparisons test. **b** Anti-HA pulldown using transfected HEK293T cells. **c** Anti-GFP pulldown using MCF-7 cells expressing YFP or YFP-NLS-PFN1. Details for quantification and normalization of SNF2H intensities are described in the Methods section. **d** Anti-SNF2H pulldown using parental MCF-10A cells. **e**, **f** PLA between HA-PFN1 and endogenous SNF2H in HeLa cells. Scale bar, 5 µm. Around 500 nuclei were quantified as in (1**b**). **g**, **h** DNA fiber analysis in MCF-10A cells. **i**, **j** DNA fiber analysis in HEK293T cells. **k** DNA fiber analysis in MCF-7 cells. For (**g**–**k**), at least 300 fibers were analyzed per condition. **l** Percentages of DNA fibers representing newly fired origins (CIdU only and CIdU-IdU-CIdU) in MCF-10A cells with SNF2H or PFN1 knockdown or PFN1 overexpression. Data are mean ± SEM of *n* = 2 independent experiments with around 3000 fibers per group. *P*-values were based on One-Way ANOVA analysis and Dunnett’s multiple comparisons test. **m** Control and PFN1 knockdown MCF-10A cells were synchronized in late G1 by double thymidine block followed or not with 2 h release into S phase. Cells were lysed with RIPA buffer, and insoluble (chromatin-enriched) vs. soluble fractions were analyzed by Western blot. **n**, **o** MCF-10A cells were lysed with RIPA and analyzed as in **m**. Details for protein quantification and normalization are described in the Methods section. **p** PLA between endogenous SNF2H and biotinylated EdU in MCF-10A cells with PFN1 knockdown/rescue or overexpression. Scale bar, 5 µm. PLA intensities were quantified by image J^95^ and shown as mean ± SEM of around 200 positive cells per condition. *P*-values for PLA and DNA fiber data were based on Kruskal–Wallis test with Dunnett’s multiple comparisons as in Fig. 1. For all statistical tests, *p < 0.05; **p < 0.01; ***p < 0.001; ****p < 0.0001; ns, not significant. All results were confirmed by at least two independent experiments. Source data are provided as a Source Data file.

Replication of chromatinized DNA requires nucleosome remodeling which is particularly important for dense heterochromatin regions. Heterochromatin replication typically occurs in late S-phase and this process depends on chromatin relaxation by ATP-dependent remodeling enzymes^50^. Interestingly, by examining the nuclear patterns of EdU incorporation into replicating DNA^51–53^, we found that PFN1 knockdown significantly decreased the percentage of cells in late S-phase while PFN1 overexpression in a PLP-dependent fashion showed an opposite effect (Fig. 2a), implicating that PFN1 may be important for chromatin relaxation. Consistent with this idea, limited chromatin digestion by micrococcal nuclease (MNase), which preferentially cuts within inter-nucleosomal linkers, showed that PFN1 knockdown decreased chromatin accessibility in MCF-10A cells, as evidenced by the upshift of the digested DNA species towards higher molecular weights (Supplementary Fig. 2a, e). Conversely, PFN1 overexpression increased chromatin accessibility, and such an effect was abolished by the S137D but not Y59A mutation (Supplementary Fig. 2b, e). Importantly, digestion of BrdU-labeled chromatin followed by anti-BrdU Western blot revealed that the observed changes in chromatin accessibility caused by PFN1 knockdown and overexpression occurred at replication forks (Supplementary Fig. 2c, d).

Interested in finding the PLP-containing protein mediating PFN1 effect on chromatin compactness, we looked for clues within our recently published interactome data based on NLS-PFN1 pulldown/MS analysis^40^. No chromatin remodeling enzymes were identified among the top 37 high-confidence hits which specifically interact with NLS-PFN1(WT) but not NLS-PFN1(S137D). However, among the lower confidence hits we found SNF2H/SMARCA5, a well-known ATP-dependent nucleosome sliding enzyme which contains seven consecutive prolines in its N-terminus (aa 7–13)^54,55^. Importantly, prior studies found that SNF2H is essential for heterochromatin replication^56^, and we confirmed that SNF2H increased chromatin accessibility similarly to PFN1 by MNase digestion (Supplementary Fig. 2f–h). By pulldown and PLA assays, we confirmed that PFN1 and SNF2H interact in a PLP-binding but not actin-binding dependent manner (Fig. 2b–f).

Next, we examined by DNA fiber assay whether SNF2H functionally underlies the positive effect of Pfn1 on DNA replication. We observed that SNF2H knockdown significantly decreased DNA fiber tract length (Fig. 2g and Supplementary Fig. 3a), and more importantly, prevented the ability of PFN1 overexpression to increase fiber tract length (Fig. 2h). Conversely, the decrease in DNA replication caused by PFN1 knockdown was effectively rescued by SNF2H overexpression, which in itself increased replication relative to control (Fig. 2i). Nonetheless, co-overexpression or co-knockdown of PFN1 and SNF2H did not further promote or inhibit DNA replication compared to single manipulations alone (Fig. 2j, k), suggesting that they are functionally epistatic. These data collectively supported the idea that PFN1 positively regulates SNF2H function during DNA replication fork progression.

A prior study reported that SNF2H promotes MCM loading onto DNA replication origins and facilitates firing^57^. Indeed, we detected reduced frequency of origin firing in SNF2H knockdown MCF-10A cells by quantifying the percentages of CIdU and CIdU-IdU-CIdU fibers (Fig. 2l). Interestingly, PFN1 phenocopies SNF2H and promotes origin firing in a PLP-dependent fashion (Fig. 2l). Cellular fractionation showed that chromatin levels of MCM3 and PCNA were decreased by PFN1 and SNF2H knockdown and increased by PFN1 overexpression in a PLP-dependent manner (Fig. 2m–o, Supplementary Fig. 3b and Supplementary Fig. 3c). EdU-PCNA PLA showed reduced level of PCNA on nascent DNA in PFN1 knockdown cells and the effect was rescued by wild type but not S137D mutant of PFN1 (Supplementary Fig. 3d, e). The PLP-dependent effects of PFN1 on chromatin association of PCNA and MCM3 occurred in the absence of their corresponding changes on the protein or mRNA levels (soluble fractions in Fig. 2m–o, and Supplementary Fig. 3b, c and f). Similar to the dominant effect of SNF2H over PFN1 on replication fork speed (Fig. 2h), SNF2H loss abolished the ability of PFN1 overexpression to increase MCM3 on chromatin (Fig. 2o). Interestingly, there appeared to be mutual dependence of chromatin-binding between PFN1 and SNF2H since depleting either protein inhibits chromatin-binding of the other (Fig. 2n, o, and Supplementary Fig. 3b). Using EdU-SNF2H PLA, we further confirmed that PFN1 indeed promotes SNF2H binding to nascent DNA in a PLP-binding but not actin-binding-dependent manner (Fig. 2p). Taken together, these results suggested that PFN1 binds SNF2H and positively regulates SNF2H function as a nucleosome remodeling factor to decondense chromatin and promote both DNA replication origin firing and fork progression.

### PFN1 increases the stalling of stressed DNA replication forks

**Fig. 3:**
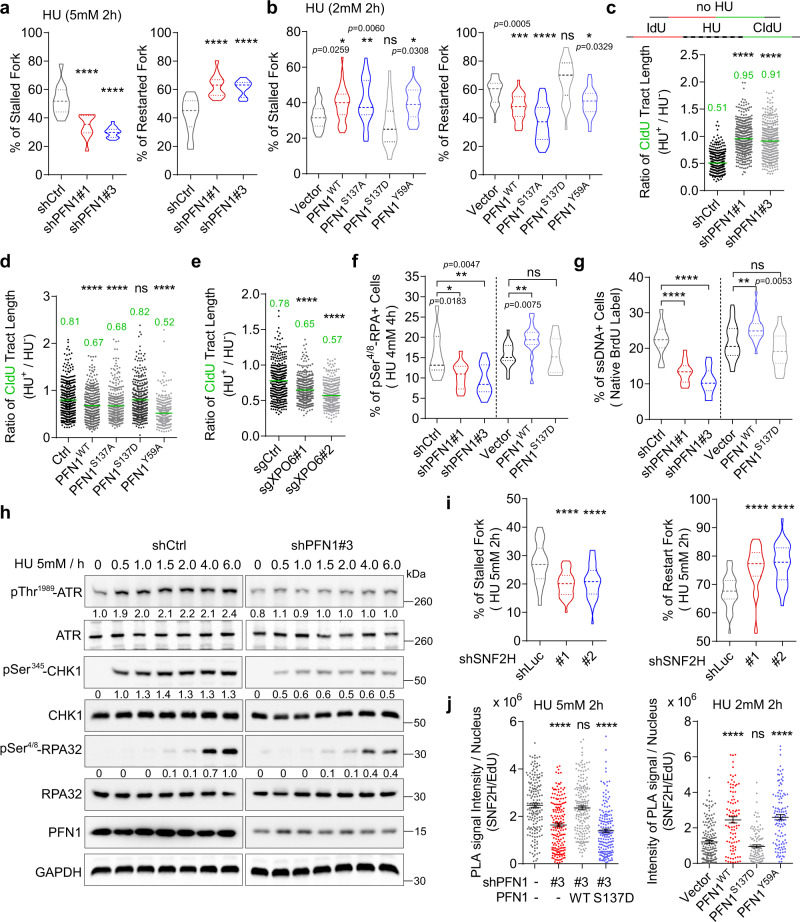
PFN1 increases the stalling of stressed DNA replication forks. **a**, **b** Percentages of DNA fibers representing stalled (IdU only) and restarted forks (dual IdU-CIdU) in MCF-10A cells sequentially labeled with 25 μM IdU and 250 μM CIdU for 30 min with 2 h HU treatment in the middle. Data represent mean ± SEM of *n* = 2 independent experiments with around 2000 fibers per group. **c**–**e** Ratios of CIdU tract lengths in dual-labeled (IdU-CIdU) MCF-10A cells with and without HU in the middle. HU was used at 5 mM in **c** and 2 mM in **d** and **e**. CIdU labeling immediately followed IdU under the no-HU condition. Individual CIdU tract lengths of HU-treated fibers were normalized to mean CIdU lengths of untreated fibers. For **c**–**e**, at least 300 fibers were analyzed per condition. **f** Percentages of pSer^4/8^-RPA32-positive MCF-10A cells following HU exposure (4 mM, 4 h) and immunofluorescence staining. **g** Same cells as in (**f**) were labeled with 10 μM BrdU for 24 h, treated with 4 mM HU for 4 h, and immuno-stained for BrdU under native conditions. Percentages of BrdU-positive cells were analyzed by ImageJ. Data in **f**, **g** are mean ± SEM of *n* = 3 independent experiments with around 3000 cells per group. **h** Western blot analysis of whole cell extracts of MCF-10A cells after HU (5 mM) exposure. Equal amounts of proteins from control and PFN1 knockdown cells were loaded on separate gels but analyzed in parallel under identical conditions. Phospho-proteins were normalized to GAPDH. **i** Percentages of stalled and restarted forks in MCF-10A cells labeled with IdU and CIdU with HU treatment in the middle as in **a**, **b**. Data are mean ± SEM of *n* = 4 independent experiments with around 3000 fibers per group. **j** PLA between endogenous SNF2H and EdU in MCF-10A cells after 2 h HU treatment. PLA intensities of around 200 positive cells per condition were analyzed and shown as mean ± SEM. *P*-values in (**a**, **b**, **f**, **g**, **i**) were based on One-Way ANOVA and Dunnett’s multiple comparisons test. *P*-values in (**c**, **d**, **e**, **j**) were based on Kruskal–Wallis test with Dunnett’s multiple comparisons. For all statistical tests, *p < 0.05; **p < 0.01; ***p < 0.001; ****p < 0.0001; ns, not significant. Source data are provided as a Source Data file.

Given the importance of PFN1 for normal DNA replication, we next investigated whether it also plays a role in fork regulation during replication stress. We labeled MCF-10A cells first with IdU, induced global replication stress with hydroxyurea (HU), and subsequently labeled with CIdU. Quantification of the relative abundance of distinct types of fibers revealed that the percentage of HU-induced stalled forks (IdU only) was significantly decreased in PFN1 knockdown cells while the frequency of restarted forks (dual-color) was increased (Fig. 3a). Conversely, PFN1 overexpression, in a PLP-dependent fashion, increased the frequency of HU-induced fork stalling and decreased fork restart (Fig. 3b). By examining the relative shortening of the CIdU tracts after HU treatment, we found that it was significantly attenuated in PFN1 knockdown cells while exacerbated in PFN1 overexpressing cells in a PLP-dependent fashion which was mimicked by XPO6 knockdown (Fig. 3c–e, and Supplementary Fig. 4a–c). Similar PLP-dependent effect of PFN1 on the relative shortening of post-HU CIdU tracts was also observed in MCF-7 cells (Supplementary Fig. 4d). These data collectively indicated that, contrary to its positive regulation of unperturbed fork progression, nuclear PFN1 increases the stalling of stressed forks.

Consistent with the fork stalling phenotype detected by DNA fiber analysis, we observed corresponding changes in single-stranded DNA (ssDNA) levels based on in situ staining for total RPA32 (in S-phase cells indicated by EdU) and pSer^4/8^-RPA32 and non-denaturing BrdU labeling. Data from both approaches showed that PFN1 increases the percentages of ssDNA-positive cells upon HU treatment in a PLP-dependent manner (Fig. 3f, g, and Supplementary Fig. 4e, f). By Western blot, we detected decreased and increased HU-induced ATR signaling (ATR autophosphorylation and CHK1 and RPA phosphorylation) in response to PFN1 knockdown and overexpression, respectively (Fig. 3h, Supplementary Fig. 4g and Supplementary Fig. 4h). This finding is consistent with the increasing effect of PFN1 on the number of fired origins and the consequent buildup of ATR-activating ssDNA/dsDNA junctions due to stress-induced fork stalling^58,59^.

Given the close functional relationship between PFN1 and SNF2H during normal replication, we next examined whether SNF2H phenocopies PFN1 under replication stress. Indeed, in HU-treated cells, SNF2H knockdown decreased the frequency of stalled forks, increased the frequency of restarted forks, decreased the relative shortening of post-HU tract lengths, and lowered CHK1 phosphorylation level (Fig. 3i, and Supplementary Fig. 4i–k), all of which mimic the effects of PFN1 loss. Further, EdU-SNF2H PLA showed that, as in unperturbed cells (Fig. 2p), PFN1 increased SNF2H level at stressed forks upon HU treatment in a PLP-dependent fashion (Fig. 3j, Supplementary Fig. 4l and Supplementary Fig. 4m). These data suggested that PFN1 and SNF2H function together to promote stalling of stressed forks.

### PFN1 decreases the stability of stressed DNA replication forks

**Fig. 4:**
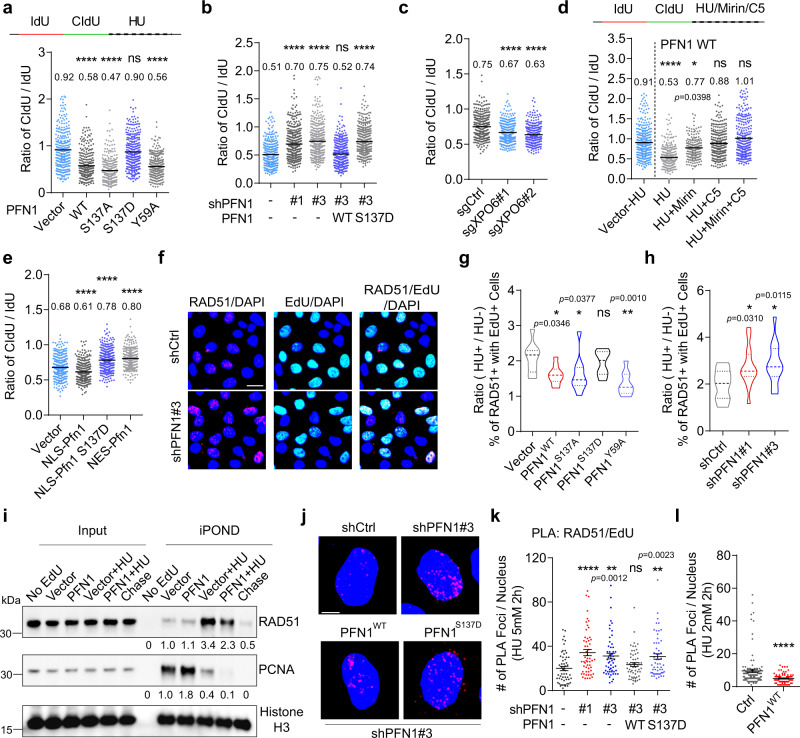
PFN1 decreases the stability of stressed DNA replication forks. **a**–**c** MCF-10A cells with PFN1 overexpression (**a**), PFN1 knockdown/rescue (**b**), and XPO6 knockout (**c**) were sequentially labeled with 25 μM IdU and 250 μM CIdU for 30 min each and treated for 2 h with HU (2 mM in **a** and 4 mM in **b**, **c**). Ratios of CIdU/IdU tract lengths are shown. **d** CIdU/IdU ratios in control or PFN1-overexpressing MCF-10A cells labeled with IdU and CIdU and subsequently treated with HU (2 mM, 2 h) in the absence or presence of 50 µM Mirin, C5, or Mirin/C5. **e** CIdU/IdU ratios in MCF-7 cells expressing YFP, YFP-NLS-PFN1(WT or S137D), or YFP-NES-PFN1(WT) labeled and treated with HU (4 mM, 2 h) as in (**a**–**d**). For (**a**–**e**), at least 300 fibers were analyzed per condition. *P*-values were based on Kruskal–Wallis test with Dunnett’s multiple comparisons. **f** Representative images of control and PFN1 knockdown MCF-10A cells co-stained for RAD51 foci and EdU following 25 min EdU pulse at 10 µM and 2 h treatment of HU at 4 mM. Scale bar, 25 µm. **g**, **h** Percentages of untreated or HU-treated PFN1 overexpression (**g**) or knockdown (**h**) MCF-10A cells stained positive for both RAD51 foci (>5 per cell) and EdU (as in **f**) were quantified and expressed as ratios. Shown are mean ± SEM of *n* = 2 independent experiments with around 2000 cells in each group. *P*-values were based on One-Way ANOVA and Dunnett’s multiple comparisons test. **i** iPOND/WB analysis of control or PFN1-overexpressing HEK293T cells labeled with 10 µM EdU for 25 min followed or not with 5 mM HU treatment for 2 h. RAD51 and PCNA levels in the iPOND samples were normalized to histone H3. **j**–**l** RAD51-EdU PLA in PFN1 knockdown/rescue (**j**, **k**) or overexpression (**l**) MCF-10A cells treated for 2 h with 5 mM or 2 mM HU. Scale bar, 5 µm. Shown are mean ± SEM of PLA foci number per nucleus of around 100 positive cells per condition. *P*-values were based on Kruskal–Wallis test with Dunnett’s multiple comparisons in **k** and two-sided Mann–Whitney test in **l**. For all statistical tests, *p < 0.05; **p < 0.01; ****p < 0.0001; ns, not significant. All results were confirmed by at least two independent experiments. Source data are provided as a Source Data file.

Many studies showed that stalled replication forks undergo nuclease attack in the absence of proper protection^15^. To determine if PFN1 plays a role in the stability of stalled forks, we first examined the relative changes in the pre-HU IdU tract lengths in the aforementioned (IdU-HU-CIdU) DNA fiber experiments (Fig. 3c–e). We found that PFN1 overexpression, in a PLP but not actin-binding-dependent manner, increased the shortening of pre-HU IdU tract lengths which was phenocopied by XPO6 loss (Supplementary Fig. 5a–c) whereas PFN1 knockdown prevented their shortening (Supplementary Fig. 5d). These data suggested that PFN1 increases the degradation of nascent DNA at stress-induced stalled forks. To more accurately test this, we sequentially labeled MCF-10A and MCF-7 cells with IdU and CIdU, treated them with HU, and quantified the ratios of CIdU/IdU tract lengths of dual-color fibers to assess nascent DNA degradation^47^. Data based on this experimental scheme completely agreed with those based on pre-HU tract length, and we further showed that the fork-protective effect of PFN1 knockdown could be reversed by WT but not the S137D mutant of PFN1 (Fig. 4a–c, and Supplementary Fig. 5e–g). Simultaneous treatment of HU and chemical inhibitors of MRE11 (Mirin)^60^ and DNA2 (C5)^61^, two main DNA nucleases involved in stalled fork resection, significantly rescued PFN1-induced nascent DNA degradation with C5 being more effective than Mirin (Fig. 4d). Further, similar to untagged PFN1 overexpression and XPO6 knockdown, NLS-PFN1, in a PLP-dependent manner, increased HU-induced nascent DNA degradation while NES-PFN1 showed no effect (Fig. 4e). Thus, nuclear PFN1 promotes nascent DNA degradation at stressed replication forks.

RAD51 is a central stabilizer of stalled replication forks by forming nucleofilaments on ssDNA and protecting nascent DNA from nucleolytic degradation^15,62,63^. To determine if the fork-destabilizing effect of PFN1 involves RAD51, we used several methods to detect RAD51 on DNA. First, we performed in situ RAD51 foci staining in mock or HU-treated MCF-10A cells which were labeled or not with EdU. Within all cells (Supplementary Fig. 5h, i) or EdU-positive S-phase cells (Fig. 4f–h), the HU-induced increase of RAD51 foci was reduced by PFN1 overexpression in a PLP-binding but not actin-binding-dependent manner and conversely augmented by PFN1 knockdown. Next, we performed iPOND/WB assay using mock or HU-treated HEK293T cells. HU expectedly increased the binding of RAD51 to nascent DNA but decreased that of PCNA. Though showing little effect on the low basal level of RAD51 on nascent DNA in unperturbed cells, PFN1 overexpression significantly reduced RAD51 level on nascent DNA in HU-treated cells (Fig. 4i). Notably, total RAD51 protein level was not reduced by PFN1 (input, Fig. 4i) although *RAD51* mRNA was upregulated by PFN1 which required both PLP and actin-binding (Supplementary Fig. 5j, k). Transcription of several additional genes (*BRCA1*, *ATR*, *CHK1*) functionally important during DNA replication stress response was either not significantly affected by PFN1 or in a way concordant with the PLP-dependent fork-destabilizing effect of PFN1 (Supplementary Fig. 5j, k). Interestingly, iPOND/WB showed a similarly increasing effect of PFN1 on the basal interaction of PCNA with nascent DNA as the EdU-PCNA assay (Supplementary Fig. 3d, e). However, in HU-treated cells, the already reduced PCNA on nascent DNA was further decreased by PFN1, consistent with its worsening effect on fork stalling known to trigger PCNA loss from nascent DNA^1,3^. Last, we performed EdU-RAD51 PLA using HU-treated PFN1 knockdown/rescue and overexpression MCF-10A cells which further confirmed that PFN1 lowers the level of RAD51 on nascent DNA in a PLP-dependent manner (Fig. 4j–l). Taken together, our data suggested that PFN1 increases stressed fork degradation by destabilizing RAD51.

### PFN1 increases genome instability during replication stress

**Fig. 5:**
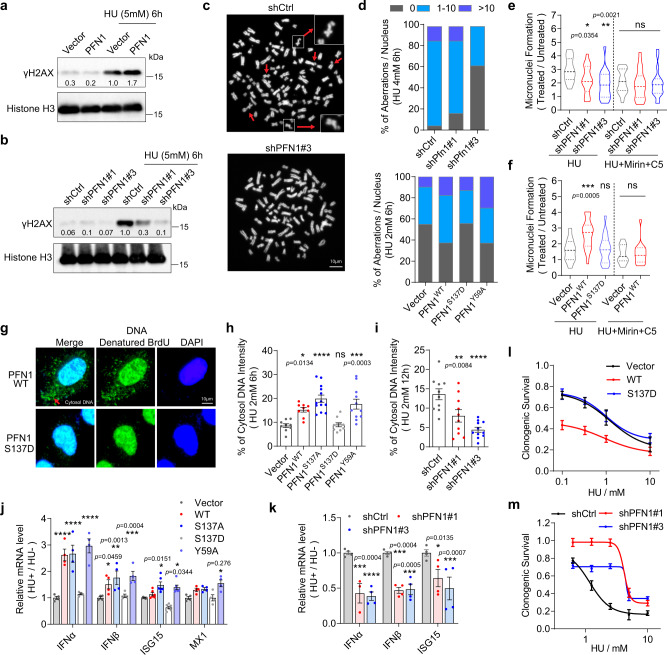
PFN1 increases genome instability during replication stress. **a**, **b** γH2AX induction in MCF-10A cells following HU treatment (5 mM, 6 h). Insoluble fractions after RIPA lysis were analyzed by Western blot. γH2AX was normalized to histone H3. **c**, **d** Metaphase spreads in HeLa cells after 6 h HU (4 mM or 2 mM) treatment, 20 h release, and 4 h nocodazole (10 μM) treatment. Aberrant chromosomes are indicated by red arrows and representative chromosomes are magnified in the insets. Scale bar, 10 µm. Around 150 metaphases per condition were analyzed from *n* = 3 independent experiments. **e**, **f** Micronuclei in PFN1 knockdown (**e**) or overexpression (**f**) MCF-7 cells after 6 h HU treatment (4 mM in **e** and 2 mM in **f**) with and without 50 µM Mirin and C5. Relative changes in the percentages of micronuclei-positive cells under HU vs. no HU conditions are shown as mean ± SEM of *n* = 2 independent experiments with around 2000 cells per condition. **g**–**i** HeLa cells with PFN1 overexpression (**g**, **h**) or knockdown (**i**) were labeled with 10 µM BrdU (**g**, **h**) or EdU (**i**) overnight, treated with 2 mM HU for 6 h (**g**, **h**) or 12 h (**i**), released overnight, and stained for BrdU under denaturing condition or EdU by Click reaction. Scale bar, 10 µm. Cytosolic DNA intensities were expressed as percentages of total DNA. Shown are mean ± SEM of *n* = 2 independent experiments with around 500 cells in each group. *P*-values in **e**, **f** and **h**, **i** were based on One-Way ANOVA and Dunnett’s multiple comparisons test. **j**, **k** PFN1-overexpressing HEK293T cells (**j**) and PFN1 knockdown MCF-7 (**k**) cells were treated or not with 4 mM HU for 6 h, released overnight, and analyzed by RT-qPCR for the indicated genes. Shown are HU/no HU ratios of mRNA levels of each gene. Data represent mean ± SEM of *n* = 4 independent experiments. Statistical significance was determined by Two-Way ANOVA and Dunnett’s multiple comparisons test. **l**, **m** Clonogenic assays using mock and HU-treated (6 h) MCF-7 cells with PFN1 overexpression (**l**) and knockdown (**m**). Data represent mean ± SEM of *n* = 5 (**l**) and *n* = 3 (**m**) independent experiments. For all statistical tests, *p < 0.05; **p < 0.01; ***p < 0.001; ****p < 0.0001; ns, not significant. Source data are provided as a Source Data file.

Prolonged stalling of stressed forks and excessive nucleolytic resection can cause fork collapse and genome instability. Consistent with the aforementioned fork stalling and over-resection phenotypes of PFN1, we detected elevated levels of HU-induced γH2AX, a marker for DSB, in PFN1 overexpressing MCF-10A cells while it was significantly reduced by PFN1 knockdown (Fig. 5a, b). Using metaphase spread, we found that PFN1, in a PLP-dependent manner, increased the numbers of HU-induced chromosomal aberrations in HeLa and MCF-7 cells while PFN1 knockdown decreased them (Fig. 5c, d, and Supplementary Fig. 6a). Since recent studies causally linked replication fork instability and accumulation of extranuclear DNA, we examined whether they can be affected by PFN1. First, we quantified micronuclei formation in mock or HU-treated MCF-7 and HeLa cells. We found that HU-caused increase of micronuclei formation was reduced by PFN1 knockdown and increased by its overexpression in a PLP-dependent fashion (Fig. 5e, f, and Supplementary Fig. 6b). Notably, co-treatment with Mirin and C5 abolished the relative effects of PFN1 knockdown and overexpression, consistent with fork resection being the source of the phenotypes (Fig. 5e, f). Second, we treated BrdU or EdU-labeled HeLa cells with HU and quantified cytosolic DNA by denaturing immunostaining or click reaction. We found that PFN1 overexpression, in a PLP-dependent fashion, significantly increased cytosolic DNA levels while PFN1 knockdown decreased them (Fig. 5g–i, and Supplementary Fig. 6c). Consistent with that micronuclei and cytosolic DNA can trigger innate immune response^64,65^, PFN1 overexpression, in a PLP but not actin-binding-dependent manner, also elevated the HU-induced mRNA increase of type I interferons and interferon-stimulated genes (Fig. 5j), which were conversely decreased by PFN1 knockdown (Fig. 5k and Supplementary Fig. 6d). Last, clonogenic assays using multiple cell lines showed that PFN1 overexpression increased cellular sensitivity to HU in a PLP-dependent manner which was phenocopied by XPO6 knockdown while PFN1 knockdown increased cellular resistance to HU (Fig. 5l, m, and Supplementary Fig. 6e–g). Taken together, these data showed that nuclear PFN1 impairs genome integrity and cellular survival upon replication stress.

### PFN1 binds BOD1L and suppresses its fork-protective activity

**Fig. 6:**
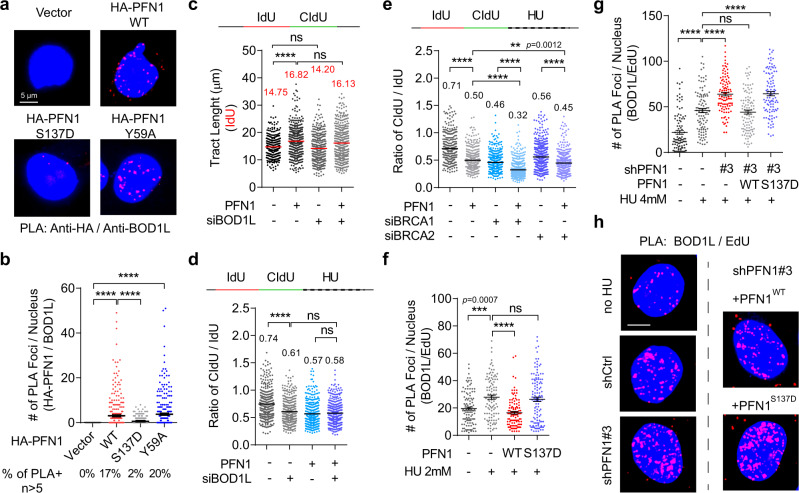
PFN1 binds BOD1L and suppresses its fork-protective activity. **a** Representative PLA images for the interaction between HA-PFN1 and endogenous BOD1L in HeLa cells. Scale bar, 5 µm. **b** Quantitative analysis of HA-PFN1/BOD1L PLA in (**a**) based on foci number per nucleus (>5 considered positive), and percentages of PLA-positive nuclei out of around 300 DAPI-positive total cells analyzed, as described in (1**c** and 2 **f**). **c** DNA fiber analysis of dual-labeled PFN1-overexpressing HeLa cells transfected with control or BOD1L siRNAs, as in (1**e**–**j**). **d**, **e** Ratios of CIdU/IdU tract lengths in dual-labeled and subsequently HU-treated (2 mM, 2 h) MCF-10A cells overexpressing PFN1 and transfected with control or siRNAs targeting BOD1L, BRCA1, or BRCA2, as described in Fig. 4**a**–**c**. In (**c**–**e**), at least 300 fibers were analyzed per condition. **f**, **g** PLA analysis between endogenous BOD1L and biotinylated EdU in PFN1-overexpressing (**f**) or PFN1 knockdown/rescue (**g**) MCF-10A cells treated or not with HU (2 mM in **f** and 4 mM in **g**) for 2 h. The numbers of PLA foci per nucleus were quantified. Data are mean ± SEM of around 100 PLA-positive cells per condition. **h** Representative PLA images for **f**, **g**. Scale bar, 5 µm. Kruskal–Wallis test with Dunnett’s multiple comparisons was used for all statistical analyses. **p < 0.01; ***p < 0.001; ****p < 0.0001; ns, not significant. All results were confirmed by at least two independent experiments.

Since fork destabilization by PFN1 depends on its ability to bind PLPs, we reasoned that a PLP-containing regulator of fork stability may underlie the effect of PFN1. Examination of our published NLS-PFN1 interactome revealed that BOD1L, an essential fork-stabilizing protein^6,34^, was a high-confidence PLP-containing PFN1 interactor. Bearing two tandem clusters of six consecutive prolines in its N-terminus, BODL1 interacted specifically with wild type but not S137D mutant of NLS-PFN1 in our prior pulldown/MS analysis^40^. BOD1L depletion was shown to trigger severe fork and genome destabilization upon replication stress which phenocopies the gain of nuclear PFN1 including DNA2-dependent over-resection of nascent DNA, ssDNA and micronuclei accumulation, chromosomal breakage, and reduced cell survival^6,34^. First, we sought to validate their physical interaction. Due to its large size (3051aa), BOD1L is prone to fragmentation and difficult to detect biochemically. However, using PLA between endogenous BOD1L and HA-PFN1, we confirmed their PLP but not actin-binding-dependent interaction (Fig. 6a, b).

Next, we used DNA fiber assay to examine the functional relationship between PFN1 and BOD1L. In unperturbed cells, BOD1L knockdown did not alter DNA fiber tract lengths or affect the ability of PFN1 overexpression to increase them (Fig. 6c and Supplementary Fig. 7a), suggesting that BOD1L does not underlie PFN1 function during normal DNA replication. When cells were treated with HU after IdU and CIdU labeling, BOD1L knockdown reduced CIdU/IdU ratio, consistent with its reported fork-protective function (Fig. 6d and Supplementary Fig. 7b). However, BOD1L knockdown could not further increase PFN1-induced fork degradation (Fig. 6d and Supplementary Fig. 7b), suggesting that functional inhibition of BOD1L is a major mechanistic underpinning of the fork-destabilizing effect of PFN1. In contrast, knocking down BRCA1 and BRCA2 worsened HU-induced fork degradation in both control and PFN1-overexpressing cells (Fig. 6e, Supplementary Fig. 7c and Supplementary Fig. 7d), indicating that PFN1 does not inhibit the fork-protective abilities of BRCA1/2. Consistent with the theory that PFN1 functionally suppresses BOD1L, EdU-BOD1L PLA showed that PFN1 overexpression significantly inhibited HU-induced fork recruitment of BOD1L in a PLP-dependent manner (Fig. 6f). Conversely, BOD1L level at stressed forks was significantly increased by PFN1 knockdown which was fully reversed by wild type but not S137D mutant of PFN1 (Fig. 6g, h). Collectively, these data suggested that PFN1 physically interacts with BOD1L and functionally inhibits its fork-protective activity during replication stress, leading to excessive nascent DNA degradation.

### PFN1 and SNF2H are required for over-resection of stressed forks

**Fig. 7:**
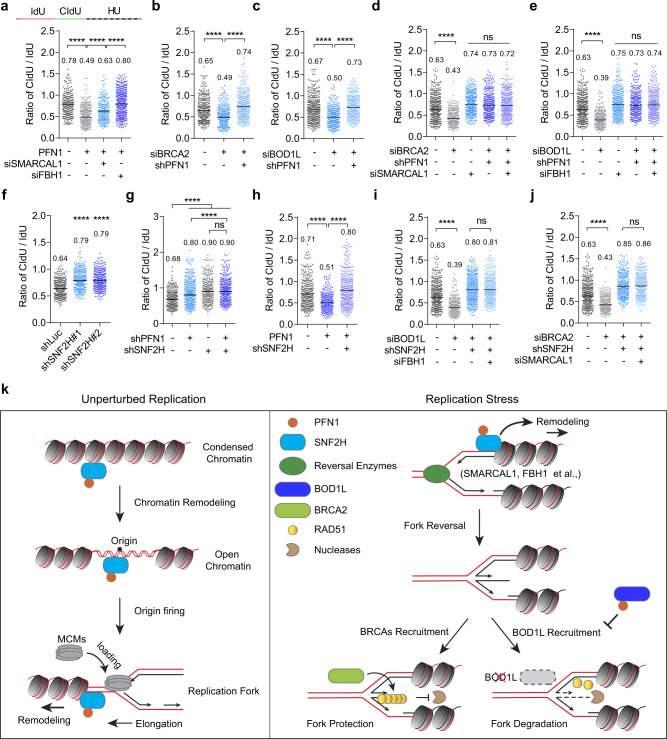
Degradation of stressed forks requires PFN1 and SNF2H. **a** Ratios of CIdU/IdU tract lengths in control or PFN1-overexpressing HeLa cells transfected with indicated siRNAs. **b**–**e** Ratios of CIdU/IdU tract lengths in control or PFN1 knockdown MCF-10A cells transfected with the indicated siRNAs. **f** Ratios of CIdU/IdU tract lengths in control or SNF2H knockdown MCF-10A cells. **g**, **h** Ratios of CIdU/IdU tract lengths in control or PFN1 knockdown or overexpression MCF-10A cells with or without SNF2H knockdown. **i**, **j** Ratios of CIdU/IdU in control or SNF2H knockdown MCF-10A cells transfected with control or gene-specific siRNAs. Cells in **a**–**j** were all dual-labeled with IdU-CIdU and subsequently treated with 4 mM HU for 2 h. Statistical significance was determined by Kruskal–Wallis test with Dunnett’s multiple comparisons. ****p < 0.0001; ns, not significant. In **a**–**j**, at least 300 fibers were analyzed per condition. All results were confirmed by at least two independent experiments. **k** Working model of the context-dependent regulation of DNA replication forks by PFN1 via its interactions with SNF2H and BOD1L. Under normal conditions, we hypothesize that the physical and functional interaction of PFN1 with SNF2H increases parental chromatin relaxation needed for both DNA replication origin firing and replication fork progression. Under stressed conditions, we hypothesize that PFN1/SNF2H remodels nucleosomes on nascent chromatin to enable general fork reversal mediated by different DNA translocases and helicases. In addition, direct binding and inhibition of BOD1L by PFN1 causes destabilization of a subset of reversed forks which are protected by BOD1L-dependent RAD51 nucleofilaments.

Fork reversal occurs frequently during replication stress and generates fork intermediates that are protected by RAD51 nucleofilaments^11,15^. Although both BRCA2 and BOD1L promote RAD51 binding to nascent DNA, recent studies suggested that they selectively protect reversed forks generated by different enzymes. While SMARCAL1 and several other DNA translocases generate reversed forks needing protection from BRCA2, forks remodeled by the DNA helicase FBH1 are specifically protected by BOD1L^19,35^. We confirmed this by showing that FBH1 or SMARCAL1 knockdown in HU-treated MCF-10A cells rescued fork degradation caused by BOD1L or BRCA2 loss, respectively (Supplementary Fig. 8a–c). Since PFN1 inhibits BOD1L during replication stress, we asked whether the excessive nascent DNA resection induced by PFN1 depends on FBH1-mediated fork remodeling. Indeed, FBH1 knockdown rescued fork degradation caused by PFN1 overexpression in HU-treated HeLa and MCF-10A cells (Fig. 7a and Supplementary Fig. 8d). Interestingly, though to a lesser extent, SMARCAL1 knockdown also rescued PFN1-induced fork degradation (Fig. 7a and Supplementary Fig. 8d), implicating that PFN1 may somehow destabilize BRCA2-dependent forks as well. This is also consistent with our finding that MRE11 inhibitor, which protects BRCA2 but not BOD1L-dependent forks, partially rescued PFN1-induced fork degradation (Fig. 4d). However, since PFN1 does not seem to block fork-protection by BRCA2 (Fig. 6e), we speculated that it may be involved in generating fork intermediates needing BRCA2 protection. But if over-produced (*e.g*. due to PFN1 overexpression), these fork intermediates may conceivably exhaust BRCA2 and become degraded. Consistent with this theory, knocking down PFN1 rescued nascent DNA degradation caused by BRCA2 loss in HU-treated cells (Fig. 7b and Supplementary Fig. 8e). Surprisingly, PFN1 knockdown also rescued nascent fork degradation caused by BOD1L loss (Fig. 7c and Supplementary Fig. 8e). This indicated that, besides directly inhibiting BOD1L, PFN1 is also important for an upstream event that is generally required for fork resection when downstream fork-protectors are deficient. Speculating that this upstream event may be fork reversal, we next examined the functional relationship between PFN1 and known fork reversing enzymes SMARCAL1 and FBH1 with regard to stressed fork resection. We found that nascent DNA degradation induced by BRCA2 loss was rescued equally well by PFN1 and SMARCAL1 knockdown to the same extent as their co-knockdown (Fig. 7d). Similarly, individual and simultaneous knockdown of PFN1 and FBH1 rescued nascent DNA degradation caused by BOD1L loss also to the same extent (Fig. 7e). These data suggested that PFN1 is important for fork reversal mediated by different pathways.

Given the close physical and functional relationship between PFN1 and SNF2H during normal DNA replication (Fig. 2) and the PLP-dependent chromatin-relaxing effects of PFN1 at unperturbed forks (Supplementary Fig. 2a–d), we examined whether chromatin at stressed forks can also be regulated by PFN1. Indeed, MNase digestion of BrdU-labeled chromatin from HU-treated MCF-10A cells revealed a similarly PLP-dependent chromatin-decondensing effect of PFN1 at stressed forks (Supplementary Fig. 9a–d). Using EdU-PLA, we detected a slightly decreased but continuous presence of HA-PFN1 on nascent DNA in a PLP-dependent manner in HU-treated HeLa cells (Supplementary Fig. 9e). PLA and co-IP assays between PFN1 and SNF2H in HU-treated HeLa and mouse chondrocyte cells showed that their interaction increased upon DNA replication stress (Supplementary Fig. 9f–i). These data suggested that PFN1 and SNF2H may function together to remodel chromatin at stressed forks and enable their reversal. Consistent with this theory, SNF2H knockdown phenocopies PFN1 loss in reducing HU-induced fork degradation, to a similar degree as their co-knockdown (Fig. 7f, g). Without SNF2H, PFN1 overexpression could not promote fork degradation in HU-treated cells (Fig. 7h). Furthermore, similar to PFN1 loss, SNF2H depletion prevented excessive fork degradation caused by BODL1 and BRCA2 depletion, regardless of whether FBH1 or SMARCAL1 was co-depleted (Fig. 7i, j and Supplementary Fig. 8f). Collectively, our data suggested that under DNA replication stress, PFN1 and SNF2H function together with different DNA remodeling enzymes to generate mixed types of reversed fork intermediates that are subsequently protected by BOD1L, BRCA2, and possibly other proteins.

## Discussion

In this paper, we identified nuclear PFN1 as a previously unrecognized regulator of DNA replication forks under both unperturbed and stressed conditions. Our study suggested that under normal conditions, PFN1 increases origin firing and replication fork progression by binding and stimulating the chromatin-remodeling activity of SNF2H. Under stressed conditions, our data suggested that PFN1 increases fork stalling and nascent DNA degradation at least via a two-pronged mechanism of stimulating SNF2H-dependent chromatin remodeling and inhibiting BOD1L-dependent fork protection (Fig. 7k). By providing mechanistic insights into the poorly understood nuclear functions of PFN1, our study implicated potential ways to improve the efficacy of genotoxic chemotherapies.

### Role of PFN1 in unperturbed DNA replication

Despite our expanding knowledge about the functional involvement of nuclear actin in DNA replication and genome integrity^66–69^, whether PFN1, an essential actin-binding protein known to exist in the nucleus and reported to associate with nascent DNA^4,49,70^, has similar nuclear functions remained completely unknown. In this study, by leveraging our prior PFN1 interactome analysis and orthogonal experimental approaches, we provided evidence that nuclear PFN1 is critically important for DNA replication. Our data support a role of nuclear PFN1 in promoting DNA replication origin firing and fork progression via binding and increasing the ability of the ATP-dependent DNA translocase SNF2H to relax chromatin. Harboring a unique PLP-binding pocket structurally separate from the actin-binding region^41,42^, PFN1 can conceivably interact with many PLP-containing proteins and functionally regulate diverse cellular processes independently of actin. Our recent discovery of the global transcriptional repression by nuclear PFN1 via its interaction with PLP-containing ENL in the Super Elongation Complex^40^, which opposes the well-established positive transcriptional regulation by nuclear actin^71,72^, supported this notion. In this paper, our separation-of-function experiments using PFN1 mutants specifically defective in binding actin vs. PLPs showed that binding to PLPs rather than actin is required for the pro-replication effect of PFN1. This indicates that the activity of nuclear PFN1 to promote unperturbed DNA replication is mediated, at least with regard to origin firing and fork progression, via actin-binding-independent mechanisms. Consistent with this theory, despite its association with several chromatin remodeling complexes such as INO80 and BAF^73–76^, nuclear actin has not been detected in any ISWI complexes containing SNF2H as the catalytic subunit.

While we are open to the possibility that multiple PLP-containing proteins may bind and collectively mediate the pro-replication function of PFN1, our data demonstrated that SNF2H is at least one major factor. Though the importance of SNF2H for nucleosome sliding has been mostly studied in the context of transcriptional control^77,78^, its essential function, together with its accessory factor ACF1/BAZ1A, in chromatin relaxation needed for DNA replication progression was described nearly two decades ago^56^. It was shown that SNF2H/BAZ1A activity is particularly important for nucleosome-dense heterochromatin regions which are typically replicated in late S phase. In addition to promoting fork progression, SNF2H was also reported to increase MCM loading at DNA replication origins and thereby promote replication initiation^57^. In this particular context, it was shown that SNF2H associates with its accessory factor WSTF/BAZ1B instead of ACF1/BAZ1A. Multiple lines of evidence in our paper support that nuclear PFN1 enhances SNF2H activity during replication origin firing and fork progression. First, PFN1 interacts with SNF2H in a PLP-dependent manner. Second, PFN1 phenocopies SNF2H and decreases chromatin compactness at replication forks in a PLP-dependent fashion. Third, PFN1 and SNF2H depletions similarly delay S phase progression. Fourth, PFN1, in a PLP-dependent manner, increases the percentage of late S-phase cells, a known phenotype of SNF2H. Fifth, PFN1 phenocopies SNF2H and increases origin firing in a PLP-dependent manner. Sixth, PFN1 increases DNA replication fork speed epistatically with SNF2H in a PLP-dependent fashion. Seventh, SNF2H overexpression can compensate for the loss of PFN1 with regard to DNA replication, but PFN1 overexpression cannot compensate for the loss of SNF2H. This is a key piece of evidence supporting a functional executor vs. regulator relationship between SNF2H and PFN1.

Currently we do not fully understand the mechanisms by which PFN1 increases SNF2H function during DNA replication. The PLP motif resides in the very N-terminus of SNF2H (7-13aa) which has no known function or interacting partners. Binding of the PLP motif by PFN1 may induce conformational change in SNF2H that increases its ATPase activity. Future structural and functional analysis using purified PFN1 and SNF2H can examine this theory. Alternatively or additionally, PFN1 binding may trigger conformational change in SNF2H to stabilize its interaction with chromatin. This theory is supported by our chromatin fractionation and PLA data that SNF2H association with nascent DNA is promoted by PFN1. In fact, when functionally idling, SNF2H samples chromatin in transient and nonspecific fashions. However, upon functional demands during DNA replication and transcriptional regulation, SNF2H becomes stably bound with chromatin to drive productive nucleosome remodeling^79–81^. Current evidence suggests that stable interactions of SNF2H with chromatin depend on specific recruitment factors and histone modifications. In the context of DNA replication, CDT1 and PCNA have been reported to recruit SNF2H directly and indirectly (via WSTF/BAZ1B) to DNA replication origins and elongating forks, respectively^57,82^. Our data that PFN1 increases MCM and PCNA levels on chromatin, new origin firing, as well as replication fork speed are all in agreement with known functions of SNF2H in promoting DNA replication initiation and progression^57,82^, and indicate that PFN1 and SNF2H function together during both stages of DNA replication. Therefore, it is tempting to speculate that the positive regulation of origin firing by SNF2H/PFN1 could increase the number of active forks containing PCNA to which more SNF2H/BAZ1B can be recruited to further promote fork progression. Nonetheless, since SNF2H exists in various complexes with different accessory factors such as ACF1/BAZ1A which is important for DNA replication but does not bind PCNA^54,56,83^, we are open to the possibility that other unknown recruitment factors may cooperate with PFN1 to increase SNF2H association with replication forks.

### Role of PFN1 during DNA replication stress

In keeping with the notion that proteins important for normal DNA replication are often involved in DNA replication stress response, we found that PFN1 significantly influences the plasticity and stability of stressed forks. By combining gain and loss-of-function approaches to increase or decrease nuclear PFN1 levels in multiple HU-treated cell lines, we observed consistent fork-stalling and destabilizing effects of PFN1 which, similarly to its effect on normal replication, depend on its ability to bind PLPs but not actin. Multiple lines of evidence support that BOD1L, a recently identified essential fork-protecting protein^6,34,35^, directly underlies the fork-stabilizing effects of nuclear PFN1. First, BOD1L contains a PLP motif and was identified as a high-confidence interacting partner of nuclear PFN1 in our prior interactome study^40^. The PLP-dependent interaction between nuclear PFN1 and BOD1L was further confirmed in this paper by PLA. Second, PFN1 gain-of-function and BOD1L loss-of-function phenocopy in causing extensive fork degradation upon HU treatment^6,34,35^. Third, the pro-degradation effect of PFN1 gain on stressed forks is epistatic with BOD1L loss, but not BRCA1 or BRCA2 loss. Fourth, fork degradation caused by PFN1 gain and BOD1L loss both depend strongly on DNA2^6^. Fifth, PFN1 gain and BOD1L loss trigger similar phenotypes of genomic instability including nuclear ssDNA accumulation, increased micronuclei formation and cytosolic DNA, and chromosomal aberrations^6^. Sixth, PFN1 gain and BOD1L loss both induce significant RAD51 destabilization on nascent DNA at stressed forks^6,34^. Seventh, fork degradation caused by PFN1 gain and BOD1L loss both require FBH1, which was found to generate fork intermediates specifically protected by BOD1L but not BRCA2^35^. Despite its established importance in fork protection during replication stress, the mechanism of action of BOD1L remains incompletely understood. BOD1L interacts with the histone methyltransferase SETD1A and they work together to promote H3K4 methylation needed for RAD51 filament stabilization on nascent DNA^34^. However, it remained completely unknown, prior to our findings in this paper, whether and how fork-protecting function of BOD1L is regulated in cells. Mechanistically, our EdU-PLA data suggested that PFN1 suppresses BOD1L function at least partially by reducing its fork interaction. Interestingly, this is reminiscent of our recent finding that nuclear PFN1 inhibits chromatin-binding of ENL at its target gene loci^40^. Since the mechanism of fork recruitment of BOD1L is currently unclear, we do not understand how interaction with PFN1 could inhibit this process. One possibility that can be tested in the future is that nuclear PFN1 may sequester certain PLP-containing proteins off chromatin and within defined subnuclear compartments such as nuclear speckles where it is known to reside. Interestingly, nuclear speckles contain high levels of PIP2 which is a well-known ligand of PFN1, and the binding site is nonoverlapping with that for PLPs^84–86^.

In addition to directly suppressing BOD1L function in fork protection, our data also suggested an upstream effect of nuclear PFN1 which precedes fork protection by both BOD1L and BRCA2. Interestingly, loss of SNF2H and PFN1 prevented fork degradation caused by both BOD1L and BRCA2 depletions. These findings have two important implications. First, PFN1 and SNF2H function together upstream of fork protection independently of the identity of the protecting factors. Second, nucleosome remodeling by SNF2H, positively regulated by PFN1, influences the stability of stalled replication forks besides its role in unperturbed DNA replication. In fact, two prior studies are consistent with our findings. First, loss of SNF2H but not the closely related SNF2L was found to decrease HU-induced DNA breaks indicated by γH2AX^2^. Second, SNF2H and its cofactor BAZ1B were enriched in CPT-induced stalled forks, and loss of BAZ1B decreases CPT-induced DNA damage and cell death^7^. Although the latter study did not directly assess the loss-of-function effect of SNF2H, these data are consistent with our finding that loss of SNF2H prevented the degradation of stressed forks. Importantly, BAZ1B loss was found to decrease the frequency of CPT-induced fork reversal based on EM analysis^7^, the only study so far, to our knowledge, which indirectly showed a role of SNF2H in fork reversal upon replication stress. Since fork reversal is a double-edged sword and an established enabling event for extensive degradation of nascent DNA in the absence of proper protection by proteins such as BOD1L and BRCA2, our findings served as additional evidence supporting an important role of SNF2H in promoting fork reversal. Much of our knowledge regarding fork reversing enzymes (DNA helicases or translocases) relied on in vitro assays using naked model DNA substrates^11,16,18–21,87^. However, within cells nucleosomes are rapidly assembled onto newly synthesized DNA in high density^23^. Though rarely discussed in the literature, this poses a conceivable barrier to the unwinding of daughter ssDNA strands from their complementary parental strands necessary for their self-annealing into the reversed DNA arm. In other words, nucleosome mobilization and unwrapping are expected to be an inherent part of the reversal process. We thus hypothesize that SNF2H, under positive regulation by PFN1, may slide the nucleosomes on the two daughter dsDNA arms in the opposite direction from fork junction to facilitate DNA unwinding and reannealing mediated by known DNA translocases and helicases. Consistent with this theory, we detected PLP-dependent chromatin relaxation by PFN1 at stressed forks and epistatic rescue of fork degradation by depleting PFN1 or SNF2H with FBH1 and SMARCAL1 in BOD1L or BRCA2-deficient cells. Therefore, our data suggested that nucleosome remodeling by SNF2H/PFN1 may be a previously unknown but critically important step of fork reversal functionally integrated into diverse fork-remodeling pathways. Future experiments based on EM are warranted to test this theory.

An important question awaiting future elucidation is why does nuclear PFN1 simultaneously promote fork reversal (a genome-protective mechanism) and inhibit fork-protection by BOD1L, which seems self-contradictory. Although we cannot answer this question at the moment, there is no doubt that PFN1 has complicated nuclear functions many of which remain to be discovered and reconciled. Our prior and current findings that nuclear PFN1 influences transcriptional elongation and DNA replication forks by its interaction with PLP-containing ENL^40^, SNF2H, and BOD1L likely reflect the tip of the iceberg for its functional involvement in various nuclear processes which may collectively determine the fate of replication forks during stress. Notably, recent studies of RADX revealed that differential amounts of RAD51 are required to promote fork reversal vs. stabilization^8,37,38,88^. While too little RAD51 is detrimental to stalled forks due to the lack of protection, too much RAD51 causes uncontrolled fork remodeling, stalling and collapse in unperturbed cells^8,37,38,88^. Therefore, properly balancing the functions of most, if not all, regulators of replication forks is likely important to maintain genome stability. Though speculative, fine tuning of the activities of various PLP-containing ligands (SNF2H, BOD1L, and others) at the forks may be an essential function of nuclear Pfn1 under both normal and stressed conditions. As for BOD1L, it is possible that limiting its activity by physiologically relevant amount of nuclear Pfn1 may be necessary to maintain an optimal local level of H3K4 methylation and RAD51 that are compatible with the desired balance between fork progression and stability. In contrast, high levels of nuclear PFN1, such as those induced by PFN1 overexpression and XPO6 depletion, would over-inhibit BOD1L and cause severe fork instability. Nevertheless, our data strongly suggested that increasing nuclear PFN1 by inhibiting XPO6 could be a promising therapeutic approach to sensitize cancer cells, which frequently overexpress XPO6^40^, to replication stress-inducing chemotherapies.

## Methods

### Cell culture

All cell lines except the mouse chondrocytes^40,48^ were purchased from ATCC and authenticated and tested for mycoplasma. MCF-10A cells were grown in DMEM/F12 plus 5% or 10% horse serum and supplements (50 μg/mL gentamycin, 20 ng/mL EGF, 0.5 mg/mL hydrocortisone, 100 ng/mL cholera toxin, and 10 μg/mL insulin). MCF-7 and MDA-MB-231 cells were grown in RPMI1 1640 plus 5% or 10% fetal bovine serum (FBS) and supplements (50 μg/mL gentamycin, 1 mM sodium pyruvate, 10 mM HEPES and 4.5 g/L glucose). HeLa, HEK293T, and mouse chondrocytes were grown in high glucose DMEM plus 5% fetal bovine serum and 50 μg/mL gentamicin.

### Antibodies

Primary antibodies used for Western blot are as follows: rabbit anti-Pfn1 (CST, #3246), rabbit anti-SNF2H (EMD Millipore, #ABE1026), rabbit anti-BOD1L (gift from Grant S. Stewart lab), rabbit anti-RAD51 (Merck Millipore, #PC130), mouse anti-PCNA (Santa Cruz, #sc-56), mouse anti-MCM3 (Santa Cruz, #sc-390480), mouse anti-Polδ (Santa Cruz, #sc-17776), mouse anti-GAPDH (Santa Cruz, #sc-47724), rabbit anti-Histone H3 (CST, #4499), mouse anti-HA-tag (BioLegend, #MMS-101P), rabbit anti-pThr^1989^-ATR (CST, #30632), rabbit anti-ATR (CST, #13934), rabbit anti-pSer^345^-CHK1 (CST, #2348), mouse anti-CHK1 (CST, #2360), rabbit anti-pSer^4/8^-RPA32 (Bethyl, #A300-245A), mouse anti-RPA32 (Santa Cruz, #sc-56770), rabbit anti-γH2AX (CST, #9718), Rabbit anti-BRCA1 (Bethyl, #A301-377), Rabbit anti-BRCA2 (Bethyl, #A303-434), Rabbit anti-SMARCAL1 (CST, #44717), mouse anti-FBH1 (Santa Cruz, #sc-81563), rabbit anti-XPO6 (ThermoFisher, #PA5-31813). Primary antibodies for immunofluorescence staining are as follows: mouse anti-RPA32 (Santa Cruz, #sc-56770), rabbit anti-pSer^4/8^-RPA32 (Bethyl, #A300-245A), rabbit anti-RAD51 (Merck Millipore, #PC130), mouse anti-BrdU (Becton Dickinson, #37580) and rat anti-BrdU (Novus Biologicals, #NB500169; Abcam, #ab6326). Primary antibodies for immunoprecipitations are as follows: mouse anti-GFP (DSHB, #DSHB-GFP-12E6), mouse anti-HA-tag (BioLegend, #MMS-101P), mouse anti-SNF2H (Santa Cruz, #sc-365727) and control mouse IgG (Santa Cruz, #sc-2025). Primary antibodies for PLA: mouse anti-Biotin (Jackson ImmunoResearch, #200-002-211), rabbit anti-Biotin (Bethyl, #A150-109A), mouse anti-HA tag (BioLegend, #MMS-101P), mouse anti-PCNA (Santa Cruz, #sc-56), rabbit anti-SNF2H (EMD Millipore, #ABE1026) and rabbit anti-BOD1L (gift from Grant S. Stewart lab). Secondary antibodies for Western blots are horseradish peroxidase-conjugated anti-rabbit (CST, #7074) and anti-mouse (CST, #7076). Secondary antibodies for immunofluorescence are Alexa Fluor 594-conjugated goat anti-mouse IgG (H + L) (Invitrogen, #A-11032), Alexa Fluor 488 -conjugated donkey anti-rat IgG (H + L) (Invitrogen, #A-21208), Alexa Fluor 594-conjugated goat anti-rabbit IgG (H + L) (Invitrogen, #A-11037) and Alexa Fluor 488-conjugated goat anti-mouse IgG (H + L) (Invitrogen, #A-11029).

### cDNAs, shRNAs, and sgRNAs

Three shRNAs targeting human PFN1 in lentiviral pFLRu-FH vector are GGAATTTAGCATGGATCTTCG (#1), TACGTGAATGGGCTGACACTT (#2), GTGGTTTGATCAACAAGAA (#3), and are controlled by a scrambled shCtrl (CAACAAGATGAAGAGCACCAA)^40,44^. Silent mutations resistant to shPFN1#3 were introduced in human PFN1 cDNA by QuickChange: TTG to CTC (nucleotide 367-369). Single guide RNAs in pSpCas9n(BB)-2A-Puro targeting human XPO6 and shRNAs in pLKO.1 targeting human and mouse XPO6 were previously reported^40^. Untagged PFN1 in lentiviral pFLRu-FH vector, HA-tagged PFN1 in pcDNA3 vector, and YFP tagged PFN1 with and without NES or NLS tag in lentiviral pFLRu-FH vector were described previously^40,44,45^. Two shRNAs targeting human SNF2H in lentiviral pLKO.1 vector are CGTCGAATTAAGGCTGATGTT (#1) and CGACTGCTGATGTAGTAATTT (#2), and controlled by a shRNA targeting luciferase (shLuc) as previously described^40^. Human SNF2H (Myc-DDK-tagged) ORF clone was purchased from OriGene Technologies, Inc (Cat# RC203775). Small interfering RNAs targeting human BOD1L/FAM44A (#sc-88933), SMARCAL1 (#sc-63042), BRCA1 (#sc-29219), BRCA2 (#sc-29825), and FBH1/FBXO18 (#sc-90469) were purchased from Santa Cruz Biotechnology, Inc.

### Transfections and infections

Transfections of siRNAs (100 pmole) and cDNAs (3 μg) encoding SNF2H (#RC203775, OriGene) and HA-PFN1 were performed in 6-well plates with 5–9 μl lipofectamine 2000 in Opti-MEM reduced serum medium for 4–6 h, and cells were cultured in fresh growth medium and used for further analysis within 72 h of transfection. Lentiviral production and infection were performed as previously described^40,44^, and cells were selected with 1 μg/ml puromycin for 3–4 days after the infection.

### Drugs and inhibitors

Hydroxyurea (#8627) and thymidine (#T1895) were purchased from Sigma Aldrich and dissolved in ddH2O. EdU (#900584), BrdU (#B9285), CldU (#C6891) and IdU (#I7125) were purchased from Sigma Aldrich and dissolved in DMSO. Mirin was from Sigma Aldrich (#M9948) and C5 was from AOBIOUS (#AOB9082), and both were dissolved in DMSO.

### Single-molecule DNA fiber spreading

DNA fiber spreading was performed as reported previously^6,13,19,47^. Logarithmically growing cells were first pulse labeled with 25 µM ldU, washed twice with PBS, and then labeled with 250 µM CIdU under unperturbed condition. For fork stalling condition, HU was added between IdU and CIdU pulses. For fork resection condition, HU in the absence or presence of 50 μM Mirin or C5 were added after dual labeling with IdU and CIdU. The time of labeling with IdU and CIdU is 30 min for MCF-10A, HeLa, MDA-MB-231 and HEK293T cells and 40 min for MCF-7 cells. Collected cells were resuspended in cold PBS (1500 cells per µL) on ice. 2.0 µL cells and 8.0 µL lysis buffer (0.5% SDS, 200 mM Tris-HCl pH 7.5, 50 mM EDTA) were mixed on a glass slide (#9991004, ThermoFisher), lysed for 5 min, and spread at around 20–40 degrees with a constant speed. After drying for 15 min, DNA fibers were fixed in methanol: acetic acid (3:1), denatured in 2.5 N HCl for 60 min, and blocked in 3% BSA/PBST (0.1% tween 20 in PBS) for 60 min at 37 °C. ldU and CIdU staining was performed using mouse anti-BrdU (1:20; Becton Disckson) and rat anti-BrdU (1:100; Abcam or Novus Biologicals) for 2 h at RT or overnight at 4 °C followed by Alexa 594 goat anti-mouse (1:100; Invitrogen) and Alexa 488 donkey anti-rat (1:100; Invitrogen) for 1 h at RT. Slides were mounted with Prolong Gold antifade mountant (#P36930, Invitrogen) and dried overnight at 4 °C. Fibers were imaged with a 60X objective on an epifluorescence microscope (Olympus IX70) and CellSens as the acquisition software. Tract lengths of at least 300 fibers per sample and percentages of forks were quantified in a blinded fashion using Image J and calculated as previously reported^47^. In untreated cells or cells exposed to HU between IdU and CIdU, tract lengths of IdU, CIdU, or combined IdU-CIdU of dual-color fibers were individually quantified. To study DNA degradation in cells exposed to HU following dual labeling, matched IdU and CIdU tract lengths of the same dual-color fibers were quantified to calculate the CIdU/IdU ratios.

### The Isolation of Proteins on Nascent DNA (iPOND)

iPOND was performed as described previously with minor modifications^5,89^. Parental HEK293T cells and stable HEK293T cells infected with vector control or PFN1 were used for iPOND. Six 150 mm dishes of cells per sample with ~50 million cells/dish were cultured. For capturing proteins associated with nascent DNA, cells were pulse-labeled with 10 µM EdU for 25 min. For capturing proteins at stalled forks, cells were treated with HU (5 mM) for 2 h following EdU-labeling. For thymidine chase, cells were incubated with 10 µM thymidine for 1 h after EdU labeling. Cells were crosslinked with 1% formaldehyde for 20 min, quenched with 0.125 M glycine and washed three times with PBS. They were permeabilized with 0.25% Triton X-100 in PBS (30 mL per sample) for 30 min at room temperature and washed twice with PBS. For conjugation of EdU with biotin azide, cells were subjected to Click-iT reaction in the reaction buffer (20 µM biotin azide (#B10184, Invitrogen), 10 mM sodium ascorbate (#A4034, Sigma), and 2 mM CuSO4 in PBS) (10 mL per sample) for 2 h at room temperature. Cells were washed twice with PBS and resuspended in lysis buffer (50 mM Tris-HCl, pH 8, and 1% SDS) (3 mL per sample) supplemented with protease inhibitors. Chromatin was solubilized by sonication using a microtip sonicator (30 s pulse and 30 s pause) in cold water until Lysates appear translucent. Samples were centrifuged for 10 min at 16,100 g at room temperature. Supernatants were diluted 1:1 with PBS (vol/vol) containing protease inhibitors and incubated overnight with streptavidin MyOne C1 beads (200 µL per sample) (#65001, Invitrogen) at 4 °C. Beads were washed once with lysis buffer for 5 min, once with low salt buffer (1% Triton X-100, 20 mM Tris pH 8.0, 2 mM EDTA, and 150 mM NaCl), once with high salt buffer (1% Triton, 20 mM Tris pH8, 2 mM EDTA, 500 mM NaCl), once with LiCl salt buffer (100 mM Tris, pH 8.0, 500 mM LiCl, and 1% NP-40) and once with lysis buffer. Captured proteins were eluted by boiling beads for 30 min at 95 °C in 30 µL 2X SDS sample buffer. The beads were vortexed and spun down, and the supernatants were analyzed by SDS PAGE and Western blot.

### Proximity ligation assay (PLA)

Protein-protein or protein-EdU PLA was performed as previously described^19,34^ using the Duolink® PLA kit (#DUO92101, Sigma Aldrich) according to the fluorescence protocol. Briefly, cells were seeded on Nunc® Lab-Tek II® -CC2 Chamber Slide (Sigma-Aldrich, #S6815) at 12000 cells/well density. For protein-EdU PLA, cells were pre-pulsed with 10 µM EdU for 20 min and subsequently treated or not with HU for different lengths of time. They were permeabilized with detergent extraction buffer (25 mM HEPES, pH 7.5, 50 mM NaCl, 1 mM EDTA, 3 mM MgCl2, 300 mM sucrose, and 0.5% Triton X-100) for 5–10 min on ice and then fixed with 4% paraformaldehyde for 15 min at room temperature and blocked with the Duolink ® block solution or 3% BSA in PBST (0.1% tween-20 in PBS) for 1 h at 37 °C. After blocking, cells were subjected to Click-iT reaction with biotin-azide for 30 min in click solution (20 µM biotin azide, 10 mM sodium ascorbate, and 2 mM CuSO4 in PBS) and then incubated overnight with primary antibodies in PBS including 1% BSA and 0.1% tween-20 at 4 °C. The primary antibodies are: mouse monoclonal anti-Biotin (Jackson ImmunoResearch, 1:2000), rabbit polyclonal anti-Biotin (Bethyl, 1:2000), mouse monoclonal anti-HA tag (BioLegend, 1:1000), mouse monoclonal anti-PCNA (Santa Cruz, 1:500), rabbit monoclonal anti-SNF2H (EMD Millipore, 1:1000), and rabbit polyclonal anti-BOD1L (Grant S. Stewart lab, 1:2000). PLA reaction steps including probe incubation, ligation, and amplification were performed according to the manufacturer’s instructions. Images were acquired with a 60X objective on an epifluorescence microscope (Olympus IX70) with CellSens as the acquisition software and PLA signals were quantified using Image J. PLA foci number per nucleus was quantified using Image J, and those containing >5 foci were considered positive. For EdU-SNF2H and EdU-PCNA PLA, intensities of positive nuclei were quantified by Image J as individual foci could not be easily identified due to the high levels of signals.

### Flow cytometry

MCF-10A cells were stably infected with shCTRL and shPFN1, synchronized by double thymidine block (2 mM thymidine, 18 h first block, 9 h release, 16 h second block) and then released in fresh medium for the indicated lengths of time. After release, cells were harvested, fixed in 70% ethanol at −20 °C for 2 h, and permeabilized with 0.25% Triton X-100 for 15 min at 4 °C. Cells were stained with propidium iodide containing 0.1 mg/ml RNase A and subjected to flow cytometry analysis. 30,000 cells per sample were analyzed by FACScan system (BD Biosciences). Data were analyzed by FlowJo v10.0 software using the univariate Watson model as previously described^40^. The representative images of gating strategies were provided with this paper in Supplementary Information (Supplementary Fig. 10).

### Metaphase spreads

Chromosomal aberrations were detected by metaphase spread as described previously^24,90^. HeLa and MCF-7 cells were treated with HU for 6 h and released in fresh medium for 24 h. During the last 4 h of release, 10 µM nocodazole was added. Cells were harvested by trypsinization, incubated in pre-warmed hypotonic solution (10 mM KCl, 10% FBS in PBS) for 20 min at 37 °C and fixed in pre-chilled methanol/acetic acid (3:1) solution (drop slowly) for 30 min on ice. Cells were washed twice with the fixation buffer (3:1 ratio of ethanol vs. acetic acid), and cell pellets were resuspended with 10 drops of fixative solution and dropped onto pre-chilled wet slides. When the slides were dried, chromosomes were stained with DAPI for 10 min and mounted with ProLong Gold antifade mounting medium. Images were acquired with a 60X objective on an epifluorescent microscope (Olympus IX70) and CellSens as the acquisition software. Chromosomal aberrations per metaphase were counted in Image J and analyzed by Graphpad Prism 8.0.

### Chromatin accessibility by MNase digestion and BrdU Southern-Western blotting

MNase digestion was performed as described previously with minor modifications^91^, and Southern and anti-BrdU Western blottings were performed as described previously^92,93^. Cells were labeled with 10 µM BrdU for 30 min followed or not by 2 h exposure to 4 mM HU. Harvested cell pellets (2 or 4 million per group) were suspended in the nucleus extraction buffer (10 mM HEPES, pH 7.9, 10 mM KCl, 1.5 mM MgCl2, 0.34 M Sucrose, 10% Glycerol, 1 mM DTT, protease inhibitors and 0.1% Triton X-100 added just before use) on ice for 8 min to lyse the cells. The nuclei were washed once with the digestion buffer (15 mM Tris-HCl, pH 8.0, 15 mM NaCl, 60 mM KCl, 1 mM EDTA, 0.5 mM EGTA and 5 mM CaCl_2_) and then suspended in the digestion buffer (100 µL per million cells) kept warm at 37 °C. Micrococcal nuclease (MNase) (NEB, #M0247S) (0.1 µL, 30U per group) in the digestion buffer (50 µL) was warmed to 37 °C, added to each tube of nuclei for 1 min or 3 min at 37 °C, and immediately mixed with equal volume of 2x stopping buffer (50 mM Tris-Cl, pH 8.0, 100 mM NaCl, 0.1% SDS, 100 mM EDTA, 20 µg/ml RNase A). 10 or 20 µL proteinase K (NEB, # P8107S) was added and the reactions were incubated for overnight at 55 °C. DNA was extracted by adding equal volume of 25:24:1 phenol/chloroform/isoamyl alcohol (Sigma, #P2069). DNA was subsequently precipitated by adding 1/10 volume of 3 M Na-Acetate (pH 5.2), and 2–2.5 volumes of ice-cold 100% ethanol. Samples were mixed and stored at −20 °C for at least 1 h. Precipitated DNA pellets were washed twice with room-temperature 70% ethanol and air-dried before suspension in 30–50 μL sterile TE buffer. Equal amounts of DNA samples were separated on 1.2% agarose gel, and stained with SYBR safe. Images were captured on a Gel Doc XR imaging system (Bio-Rad) and DNA intensities were quantified by Image Lab.

For Anti-BrdU immuno-blotting, Southern-Western blot was performed. Briefly, the SYBR safe-stained DNA gels were incubated in denaturing buffer (0.5 M NaOH, 1.5 M NaCl) twice for 15 min each time followed by incubation in neutralization buffer (1.0 M Tris-HCL, 3 M NaCl) for 30 min. DNA was transferred to Cytiva Amersham™ Hybond™ -N^+^ Membrane (Thermo Fisher, #45-004) by Southern blotting for overnight. DNA was then cross-linked to membrane using the UV transilluminator (Stratagene, La Jolla, CA). Membranes were incubated with 5% non-fat milk for 1 h followed by overnight incubation with anti-BrdU antibody (Becton Disckson, #37580, 1:200) at 4 °C and subsequent incubation with HRP-linked secondary antibody for 1 h at room temperature. BrdU signals were developed using ECL (Thermo Fisher, West Dura, #34076), imaged on the same Gel Doc system (Bio-Rad), and quantified by Image Lab.

### Immunofluorescence

Cells seeded on 96-well plates were treated with vehicle or HU as indicated in the figures. For EdU labeling, the cells were pulsed with 10 µM EdU for 25 min before HU treatment. Cells were permeabilized with detergent extraction buffer (25 mM HEPES, pH 7.5, 50 mM NaCl, 1 mM EDTA, 3 mM MgCl2, 300 mM sucrose and 0.5% Triton X-100) for 5–10 min on ice, fixed with 4% paraformaldehyde for 15 min at room temperature and blocked with 3% BSA in PBST (0.1% tween-20 in PBS) for 1 h at room temperature. Cells were incubated with primary antibodies for 2 h at room temperature or overnight at 4 °C followed by secondary antibodies and DAPI (1 µg/ml) for 1 h at room temperature. For EdU staining, the Click-iT reaction with Alexa 488-azide (#A10266, ThermoFisher) was performed for 30 min at room temperature (click solution: 20 µM Alexa 488-azide, 10 mM sodium ascorbate and 2 mM CuSO4 in PBS) before the incubation with primary antibodies. Images were captured on an inverted epifluorescence microscope (Olympus IX70) using a 20x objective and CellSens as acquisition software. High resolution images were captured by choosing the pixel shift function of the camera at the highest setting (4140 ×3096). ImageJ was used for merging and quantitative analysis. For RAD51 foci, cells with >5 foci were considered positive and quantified as relative percentages out of all DAPI-stained cells. For RAD51 and EdU co-staining, the percentage of dual-positive cells out of all DAPI-stained cells was analyzed. For total RPA and pSer^4/8^-RPA staining, positive cells were scored based on fluorescence intensity, and quantified as relative percentages out of all DAPI-stained cells. The primary antibodies were rabbit polyclonal anti-RAD51 (Merck Millipore, #PC130, 1:500), mouse monoclonal anti-RPA32 (Santa Cruz, #sc-56770, 1:500), rabbit polyclonal anti-pSer^4/8^-RPA32 (Bethyl, #A300-245A, 1:1000). The secondary antibodies were Alexa Fluor 488 anti-mouse or rabbit and Alexa Fluor 594 anti-rabbit or mouse (Invitrogen, 1:1000).

### Late S-phase analysis

EdU incorporation pattern was used for scoring as previously described^51–53^. Nuclei containing sparse and large EdU foci characteristic of pericentromeric heterochromatin were identified as being in late S-phase, and quantified as relative percentages out of all S-phase cells stained positive for EdU.

### Analysis of micronuclei formation

Cells were seeded in 96 well plates, treated with vehicle or HU for 6 h, and released in fresh medium for overnight. After staining with DAPI for 15 min, images were captured on an inverted epifluorescence microscope (Olympus IX70) using a 60x objective and CellSens as the acquisition software, and analyzed by Image J. The percentage of cells containing micronuclei (one or more per cell) were blindly counted and analyzed by Graphpad Prism 8.0.

### Cytosolic DNA and ssDNA Quantification

To image cytosolic DNA, cells were seeded in 96 well plates, labeled with 10 µM EdU or BrdU for overnight, treated with vehicle or HU for the indicated times as shown in the figures and released in fresh medium for overnight. For EdU labeling, the cells were subjected to Click-iT reaction with Alexa 488-azide for 30 min at room temperature (click solution: 20 µM Alexa 488-azide, 10 mM sodium ascorbate, and 2 mM CuSO4 in PBS). For BrdU labeling, the cells were subjected to immunofluorescence staining (primary antibody, mouse anti-BrdU, BD Biosciences) after denaturing DNA with 2 M HCl for 30 min at room temperature. ImageJ was used for merging and quantifying cytoplasmic DNA fluorescence by the Intensity Ratio Nuclei Cytoplasm Tool macros^40^.

For ssDNA imaging, a native BrdU labeling procedure was applied^90,94^. Cells seeded in 96 well plates were labeled with BrdU (10 µM) for overnight, treated with HU (4 mM) for 4 h, pre-extracted with the detergent extraction buffer (25 mM HEPES, pH 7.5, 50 mM NaCl, 1 mM EDTA, 3 mM MgCl2, 300 mM sucrose, and 0.5% Triton X-100) for 5–10 min on ice, and subjected to immunofluorescence staining (primary antibody, mouse anti-BrdU, BD Biosciences) under the native condition without acid denaturation by HCl. The percentage of ssDNA-positive cells was quantified by Image J.

### Immunoprecipitation, subcellular fractionation, and western blot

All immunoprecipitations (anti-HA, GFP, SNF2H) were performed as previously described using RIPA-lysed cell lysates^40^. 250U/ml Benzonase (EMD Millipore, #70746) and 1.5 mM MgCl_2_ were included in cell lysates. Binding between primary antibodies (2–5 µg) and cell lysates (0.5–1 mg) was conducted for 4 h at 4 °C, followed by the incubation with Dynabeads protein G (10–20 µl) for 4 h at 4 °C. Beads were washed 3–5 times with lysis buffer, and proteins were eluted in SDS sample buffer at 95 °C and analyzed by Western blot. Subcellular fractionation was performed as described previously^40^. For Western blot, denatured proteins were separated by SDS-PAGE, transferred onto nitrocellulose membrane, incubated with primary antibody overnight, followed by HRP-linked secondary antibody for 1 h at room temperature. The signals were detected by SuperSignal West Dura Extended Duration Substrate (#34075, Thermo Fisher) or West Femto Maximum Sensitivity Substrate (#34096, Thermo Fisher), and imaged on a Gel Doc XR imaging system (Bio-Rad). Band intensities were quantified by ImageLab, and normalized to corresponding loading controls on the same gels (GAPDH, tubulin, actin, or histone H3). In most cases, the normalized protein intensities were further normalized to the controls within the experiments (which were arbitrarily set at a value of 1). In Fig. 2b, c, after subtracting the non-specific control values (vector in b and IgG in c), SNF2H intensities in the IP samples were first normalized to those of corresponding PFN1 proteins. Next, the levels of SNF2H bound to wild type PFN1 were arbitrarily set to 1 and used for further normalization for SNF2H bound to PFN1 mutants. Unprocessed raw Western blot data are provided in the Source Data file.

### RT-qPCR

Experiments were performed as described previously with minor modifications^65^. Briefly, cells were either untreated or treated with HU (4 mM) for 6 h (for type I interferon genes) and released in fresh medium for overnight. They were harvested by trypsinization and subjected to total RNA isolation by TRIzol (#15596018, Invitrogen) according to manufacturer’s instruction. The relative gene expression was quantified by RT-qPCR as described previously^40^ and normalized to GAPDH. In brief, complementary DNAs (cDNA) were synthesized from 2 µg RNA using the high-capacity reverse transcription kit (#4368814, ThermoFisher), and quantitative PCR was performed using the PowerUP SYBR Green Master Mix (#A25743, Fisher Scientific) in accordance with the manufacturer’s instruction on a CFX96 Touch™ Real-Time PCR Detection System (Bio-Rad). Primers were designed using Primer-Blast at NCBI or based on prior paper^65^. *IFNα*: Forward, CCCATTTCAACCAGTCTAGCAG; Reverse, TGTGGGTTTGAGGCAGATC. *IFNβ*: Forward, AGGATTCTGCATTACCTGAAGG; Reverse, GGCTAGGAGATCTTCAGTTTCG. *MX1*: Forward, ATGAGCTAATCACCCTGGAG; Reverse, ATACCCAATGTCAGCAGGC. *ISG15*: Forward, CGCAGATCACCCAGAAGATCG; Reverse, TTCGTCGCATTTGTCCACCA. *TNF*: Forward, ACTTTGGAGTGATCGGCC; Reverse, GCTTGAGGGTTTGCTACAAC. *PCNA*: Forward, GGCCGAAGATAACGCGGATAC; Reverse, GGCATATACGTGCAAATTCACCA. *RAD51*: Forward, GAGACCGAGCCCTAAGGAGA; Reverse, TTGCCATTACTCGGTCCGC. *BRCA1*: Forward, AGCTGTGTGGTGCTTCTGTGGT; Reverse, TGGCTGCACAACCACAATTGGG. *ATR*: Forward, GGCCAAAGGCAGTTGTATTGA; Reverse, GTGAGTACCCCAAAAATAGCAGG. *CHK1*: Forward, ATATGAAGCGTGCCGTAGACT; Reverse, TGCCTATGTCTGGCTCTATTCTG.

### Colony survival assays

Colony survival assays were performed by seeding ~500 cells/well in 6-well plates (or proportionally in 12-well or 24-well plates) and cultured in fresh medium for 14–20 days after exposure to vehicle or HU for 6 h. Cells were quantified by Alamar blue prior to fixation with 4% paraformaldehyde and staining with 0.005% crystal violet for 2 h. Colonies were imaged and quantified for percentages of occupied areas or colony numbers in the wells by ImageJ software.

### Statistics and reproducibility

Statistical significance for DNA fiber and PLA data was determined by Kruskal–Wallis test with Dunnett’s multiple comparisons or Mann–Whitney test (two-sided) depending on the number of experimental groups. RT-qPCR data were analyzed by the Two-Way ANOVA analysis and Dunnett’s multiple comparisons test. All other statistical analyses were performed using the One-Way ANOVA analysis and Dunnett’s multiple comparisons test. For all phenotypic effects, same trends were confirmed by at least two independent experiments. *P*-values were defined as follows: ns, not significant; *p < 0.05; **p < 0.01; ***p < 0.001; ****p < 0.0001. The exact adjusted *P*-value (used for all Dunnett’s multiple comparisons) for *p < 0.05; **p < 0.01; ***p < 0.001 were provided in the figures.

### Reporting summary

Further information on research design is available in the Nature Research Reporting Summary linked to this article.

## Supplementary information

Supplementary Information

Reporting Summary

## Data Availability

The data that support this study are available from the corresponding author upon reasonable request. Source data are provided with this paper.

## Source data

Source Data

## References

[1] Sirbu BM (2011). Analysis of protein dynamics at active, stalled, and collapsed replication forks. Genes Dev..

[2] Sirbu BM (2013). Identification of proteins at active, stalled, and collapsed replication forks using isolation of proteins on nascent DNA (iPOND) coupled with mass spectrometry. J. Biol. Chem..

[3] Dungrawala H (2015). The replication checkpoint prevents two types of fork collapse without regulating replisome stability. Mol. Cell.

[4] Wessel SR, Mohni KN, Luzwick JW, Dungrawala H, Cortez D (2019). Functional analysis of the replication fork proteome identifies BET proteins as PCNA regulators. Cell Rep..

[5] Genois MM (2021). CARM1 regulates replication fork speed and stress response by stimulating PARP1. Mol. Cell.

[6] Higgs MR (2015). BOD1L is required to suppress deleterious resection of stressed replication forks. Mol. Cell.

[7] Ribeyre C (2016). Nascent DNA proteomics reveals a chromatin remodeler required for topoisomerase I loading at replication forks. Cell Rep..

[8] Dungrawala H (2017). RADX promotes genome stability and modulates chemosensitivity by regulating RAD51 at replication forks. Mol. Cell.

[9] Mukherjee C (2019). RIF1 promotes replication fork protection and efficient restart to maintain genome stability. Nat. Commun..

[10] Zellweger R (2015). Rad51-mediated replication fork reversal is a global response to genotoxic treatments in human cells. J. Cell Biol..

[11] Joseph SA (2020). Time for remodeling: SNF2-family DNA translocases in replication fork metabolism and human disease. DNA repair.

[12] Berti M (2013). Human RECQ1 promotes restart of replication forks reversed by DNA topoisomerase I inhibition. Nat. Struct. Mol. Biol..

[13] Ray Chaudhuri A (2012). Topoisomerase I poisoning results in PARP-mediated replication fork reversal. Nat. Struct. Mol. Biol..

[14] Quinet A, Lemacon D, Vindigni A (2017). Replication fork reversal: players and guardians. Mol. Cell.

[15] Berti M, Cortez D, Lopes M (2020). The plasticity of DNA replication forks in response to clinically relevant genotoxic stress. Nat. Rev. Mol. Cell Biol..

[16] Fugger K (2015). FBH1 catalyzes regression of stalled replication forks. Cell Rep..

[17] Bai G (2020). HLTF promotes fork reversal, limiting replication stress resistance and preventing multiple mechanisms of unrestrained DNA synthesis. Mol. Cell.

[18] Kile AC (2015). HLTF’s ancient HIRAN domain binds 3' DNA ends to drive replication fork reversal. Mol. Cell.

[19] Taglialatela A (2017). Restoration of replication fork stability in BRCA1- and BRCA2-deficient cells by inactivation of SNF2-family fork remodelers. Mol. Cell.

[20] Betous R (2012). SMARCAL1 catalyzes fork regression and Holliday junction migration to maintain genome stability during DNA replication. Genes Dev..

[21] Betous R (2013). Substrate-selective repair and restart of replication forks by DNA translocases. Cell Rep..

[22] Vujanovic M (2017). Replication fork slowing and reversal upon DNA damage require PCNA polyubiquitination and ZRANB3 DNA translocase activity. Mol. Cell.

[23] Stewart-Morgan KR, Reveron-Gomez N, Groth A (2019). Transcription restart establishes chromatin accessibility after DNA replication. Mol. Cell.

[24] Lemacon D (2017). MRE11 and EXO1 nucleases degrade reversed forks and elicit MUS81-dependent fork rescue in BRCA2-deficient cells. Nat. Commun..

[25] Thangavel S (2015). DNA2 drives processing and restart of reversed replication forks in human cells. J. Cell Biol..

[26] Kolinjivadi AM (2017). Smarcal1-mediated fork reversal triggers Mre11-dependent degradation of nascent DNA in the absence of Brca2 and stable Rad51 nucleofilaments. Mol. Cell.

[27] Schlacher K (2011). Double-strand break repair-independent role for BRCA2 in blocking stalled replication fork degradation by MRE11. Cell.

[28] Mijic S (2017). Replication fork reversal triggers fork degradation in BRCA2-defective cells. Nat. Commun..

[29] Schlacher K, Wu H, Jasin M (2012). A distinct replication fork protection pathway connects Fanconi anemia tumor suppressors to RAD51-BRCA1/2. Cancer Cell.

[30] Ray Chaudhuri A (2016). Replication fork stability confers chemoresistance in BRCA-deficient cells. Nature.

[31] Guillemette S (2015). Resistance to therapy in BRCA2 mutant cells due to loss of the nucleosome remodeling factor CHD4. Genes Dev..

[32] Xu S (2017). Abro1 maintains genome stability and limits replication stress by protecting replication fork stability. Genes Dev..

[33] Espana-Agusti J, Warren A, Chew SK, Adams DJ, Matakidou A (2017). Loss of PBRM1 rescues VHL dependent replication stress to promote renal carcinogenesis. Nat. Commun..

[34] Higgs MR (2018). Histone methylation by SETD1A protects nascent DNA through the nucleosome chaperone activity of FANCD2. Mol. Cell.

[35] Liu, W., Krishnamoorthy, A, Zhao, R. & Cortez, D. Two replication fork remodeling pathways generate nuclease substrates for distinct fork protection factors. *Sci. Adv*. **6**, eabc3598 (2020). 10.1126/sciadv.abc3598 PMC7673757 33188024

[36] Couch FB (2013). ATR phosphorylates SMARCAL1 to prevent replication fork collapse. Genes Dev..

[37] Adolph MB (2021). RADX controls RAD51 filament dynamics to regulate replication fork stability. Mol. Cell.

[38] Bhat KP (2018). RADX modulates RAD51 activity to control replication fork protection. Cell Rep..

[39] Jockusch BM, Murk K, Rothkegel M (2007). The profile of profilins. Rev. Physiol. Biochem Pharm..

[40] Zhu C (2021). Cancer-associated exportin-6 upregulation inhibits the transcriptionally repressive and anticancer effects of nuclear profilin-1. Cell Rep..

[41] Mahoney NM, Janmey PA, Almo SC (1997). Structure of the profilin-poly-L-proline complex involved in morphogenesis and cytoskeletal regulation. Nat. Struct. Biol..

[42] Metzler WJ, Bell AJ, Ernst E, Lavoie TB, Mueller L (1994). Identification of the poly-L-proline-binding site on human profilin. J. Biol. Chem..

[43] Petruk S (2012). TrxG and PcG proteins but not methylated histones remain associated with DNA through replication. Cell.

[44] Diamond MI (2015). Subcellular localization and Ser-137 phosphorylation regulate tumor-suppressive activity of profilin-1. J. Biol. Chem..

[45] Shao J, Welch WJ, Diprospero NA, Diamond MI (2008). Phosphorylation of profilin by ROCK1 regulates polyglutamine aggregation. Mol. Cell Biol..

[46] Wittenmayer N (2004). Tumor suppressor activity of profilin requires a functional actin binding site. Mol. Biol. Cell.

[47] Quinet A, Carvajal-Maldonado D, Lemacon D, Vindigni A (2017). DNA fiber analysis: mind the gap!. Methods Enzymol..

[48] Bottcher RT (2009). Profilin 1 is required for abscission during late cytokinesis of chondrocytes. Embo J..

[49] Stuven T, Hartmann E, Gorlich D (2003). Exportin 6: a novel nuclear export receptor that is specific for profilin.actin complexes. Embo J..

[50] de la Serna IL, Imbalzano AN (2002). Unfolding heterochromatin for replication. Nat. Genet.

[51] O’Keefe RT, Henderson SC, Spector DL (1992). Dynamic organization of DNA replication in mammalian cell nuclei: spatially and temporally defined replication of chromosome-specific alpha-satellite DNA sequences. J. Cell Biol..

[52] Fox MH, Arndt-Jovin DJ, Jovin TM, Baumann PH, Robert-Nicoud M (1991). Spatial and temporal distribution of DNA replication sites localized by immunofluorescence and confocal microscopy in mouse fibroblasts. J. Cell Sci..

[53] Maya-Mendoza A (2018). High speed of fork progression induces DNA replication stress and genomic instability. Nature.

[54] Narlikar GJ, Sundaramoorthy R, Owen-Hughes T (2013). Mechanisms and functions of ATP-dependent chromatin-remodeling enzymes. Cell.

[55] Zhou CY, Johnson SL, Gamarra NI, Narlikar GJ (2016). Mechanisms of ATP-dependent chromatin remodeling motors. Annu Rev. Biophys..

[56] Collins N (2002). An ACF1-ISWI chromatin-remodeling complex is required for DNA replication through heterochromatin. Nat. Genet.

[57] Sugimoto N, Yugawa T, Iizuka M, Kiyono T, Fujita M (2011). Chromatin remodeler sucrose nonfermenting 2 homolog (SNF2H) is recruited onto DNA replication origins through interaction with Cdc10 protein-dependent transcript 1 (Cdt1) and promotes pre-replication complex formation. J. Biol. Chem..

[58] MacDougall CA, Byun TS, Van C, Yee MC, Cimprich KA (2007). The structural determinants of checkpoint activation. Genes Dev..

[59] Zou L (2007). Single- and double-stranded DNA: building a trigger of ATR-mediated DNA damage response. Genes Dev..

[60] Dupre A (2008). A forward chemical genetic screen reveals an inhibitor of the Mre11-Rad50-Nbs1 complex. Nat. Chem. Biol..

[61] Liu W (2016). A selective small molecule DNA2 inhibitor for sensitization of human cancer cells to chemotherapy. EBioMedicine.

[62] Hashimoto Y, Ray Chaudhuri A, Lopes M, Costanzo V (2010). Rad51 protects nascent DNA from Mre11-dependent degradation and promotes continuous DNA synthesis. Nat. Struct. Mol. Biol..

[63] Petermann E, Orta ML, Issaeva N, Schultz N, Helleday T (2010). Hydroxyurea-stalled replication forks become progressively inactivated and require two different RAD51-mediated pathways for restart and repair. Mol. Cell.

[64] Mackenzie KJ (2017). cGAS surveillance of micronuclei links genome instability to innate immunity. Nature.

[65] Coquel F (2018). SAMHD1 acts at stalled replication forks to prevent interferon induction. Nature.

[66] Lamm N (2020). Nuclear F-actin counteracts nuclear deformation and promotes fork repair during replication stress. Nat. Cell Biol..

[67] Belin BJ, Lee T, Mullins RD (2015). DNA damage induces nuclear actin filament assembly by Formin -2 and Spire-(1/2) that promotes efficient DNA repair. [corrected]. eLife.

[68] Parisis N (2017). Initiation of DNA replication requires actin dynamics and formin activity. EMBO J..

[69] Caridi CP, Plessner M, Grosse R, Chiolo I (2019). Nuclear actin filaments in DNA repair dynamics. Nat. Cell Biol..

[70] Skare P, Kreivi JP, Bergstrom A, Karlsson R (2003). Profilin I colocalizes with speckles and Cajal bodies: a possible role in pre-mRNA splicing. Exp. Cell Res.

[71] Virtanen JA, Vartiainen MK (2017). Diverse functions for different forms of nuclear actin. Curr. Opin. Cell Biol..

[72] Kelpsch DJ, Tootle TL (2018). Nuclear actin: from discovery to function. Anat. Rec. (Hoboken).

[73] Shen X, Mizuguchi G, Hamiche A, Wu C (2000). A chromatin remodelling complex involved in transcription and DNA processing. Nature.

[74] Shen X, Ranallo R, Choi E, Wu C (2003). Involvement of actin-related proteins in ATP-dependent chromatin remodeling. Mol. Cell.

[75] Xie X (2018). beta-Actin-dependent global chromatin organization and gene expression programs control cellular identity. FASEB J..

[76] Zhao K (1998). Rapid and phosphoinositol-dependent binding of the SWI/SNF-like BAF complex to chromatin after T lymphocyte receptor signaling. Cell.

[77] Corona DF, Tamkun JW (2004). Multiple roles for ISWI in transcription, chromosome organization and DNA replication. Biochim Biophys. Acta.

[78] Goodwin LR, Picketts DJ (2018). The role of ISWI chromatin remodeling complexes in brain development and neurodevelopmental disorders. Mol. Cell Neurosci..

[79] Erdel F, Schubert T, Marth C, Langst G, Rippe K (2010). Human ISWI chromatin-remodeling complexes sample nucleosomes via transient binding reactions and become immobilized at active sites. Proc. Natl Acad. Sci. USA.

[80] Donovan, D. A. et al. Basis of specificity for a conserved and promiscuous chromatin remodeling protein. *eLife* **10**, e64061 (2021). 10.7554/eLife.64061 PMC7968928 33576335

[81] Varga-Weisz PD (2010). Insights into how chromatin remodeling factors find their target in the nucleus. Proc. Natl Acad. Sci. USA.

[82] Poot RA (2004). The Williams syndrome transcription factor interacts with PCNA to target chromatin remodelling by ISWI to replication foci. Nat. Cell Biol..

[83] Gioacchini N, Peterson CL (2017). Chromatin remodeling: a complex affair. EMBO Rep..

[84] Sohn RH, Chen J, Koblan KS, Bray PF, Goldschmidt-Clermont PJ (1995). Localization of a binding site for phosphatidylinositol 4,5-bisphosphate on human profilin. J. Biol. Chem..

[85] Goldschmidt-Clermont PJ, Machesky LM, Baldassare JJ, Pollard TD (1990). The actin-binding protein profilin binds to PIP2 and inhibits its hydrolysis by phospholipase C. Science.

[86] Osborne SL, Thomas CL, Gschmeissner S, Schiavo G (2001). Nuclear PtdIns(4,5)P2 assembles in a mitotically regulated particle involved in pre-mRNA splicing. J. Cell Sci..

[87] Blastyak A, Hajdu I, Unk I, Haracska L (2010). Role of double-stranded DNA translocase activity of human HLTF in replication of damaged DNA. Mol. Cell Biol..

[88] Krishnamoorthy A (2021). RADX prevents genome instability by confining replication fork reversal to stalled forks. Mol. Cell.

[89] Sirbu BM, Couch FB, Cortez D (2012). Monitoring the spatiotemporal dynamics of proteins at replication forks and in assembled chromatin using isolation of proteins on nascent DNA. Nat. Protoc..

[90] Li S (2019). Ca(2+)-stimulated AMPK-dependent phosphorylation of Exo1 protects stressed replication forks from aberrant resection. Mol. Cell.

[91] Chereji RV, Bryson TD, Henikoff S (2019). Quantitative MNase-seq accurately maps nucleosome occupancy levels. Genome Biol..

[92] Green MR, Sambrook J. Southern blotting. *Cold Spring Harb. Protoc.* https://www.mybiosource.com/learn/southern-blotting/ (2021). 10.1101/pdb.prot100487 34210769

[93] Gali VK (2018). Identification of Elg1 interaction partners and effects on post-replication chromatin re-formation. PLoS Genet.

[94] Buisson R, Boisvert JL, Benes CH, Zou L (2015). Distinct but concerted roles of ATR, DNA-PK, and Chk1 in countering replication stress during S phase. Mol. Cell.

[95] Roy S, Luzwick JW, Schlacher K (2018). SIRF: Quantitative in situ analysis of protein interactions at DNA replication forks. J. Cell Biol..

