# Supplementary material for: Profilin-1 regulates DNA replication forks in a context-dependent fashion by interacting with SNF2H and BOD1L
Source: Nat Commun. 2022 Nov 1;13:6531. doi: 10.1038/s41467-022-34310-9 (PMC9626489; doi:10.1038/s41467-022-34310-9)
Supplement: Supplementary file 1 — Supplementary Information [file 41467_2022_34310_MOESM1_ESM.pdf]

SUPPLEMENTARY INFORMATION

Profilin-1 Regulates DNA Replication Forks in a Context-Dependent Fashion by Interacting with SNF2H and BOD1L

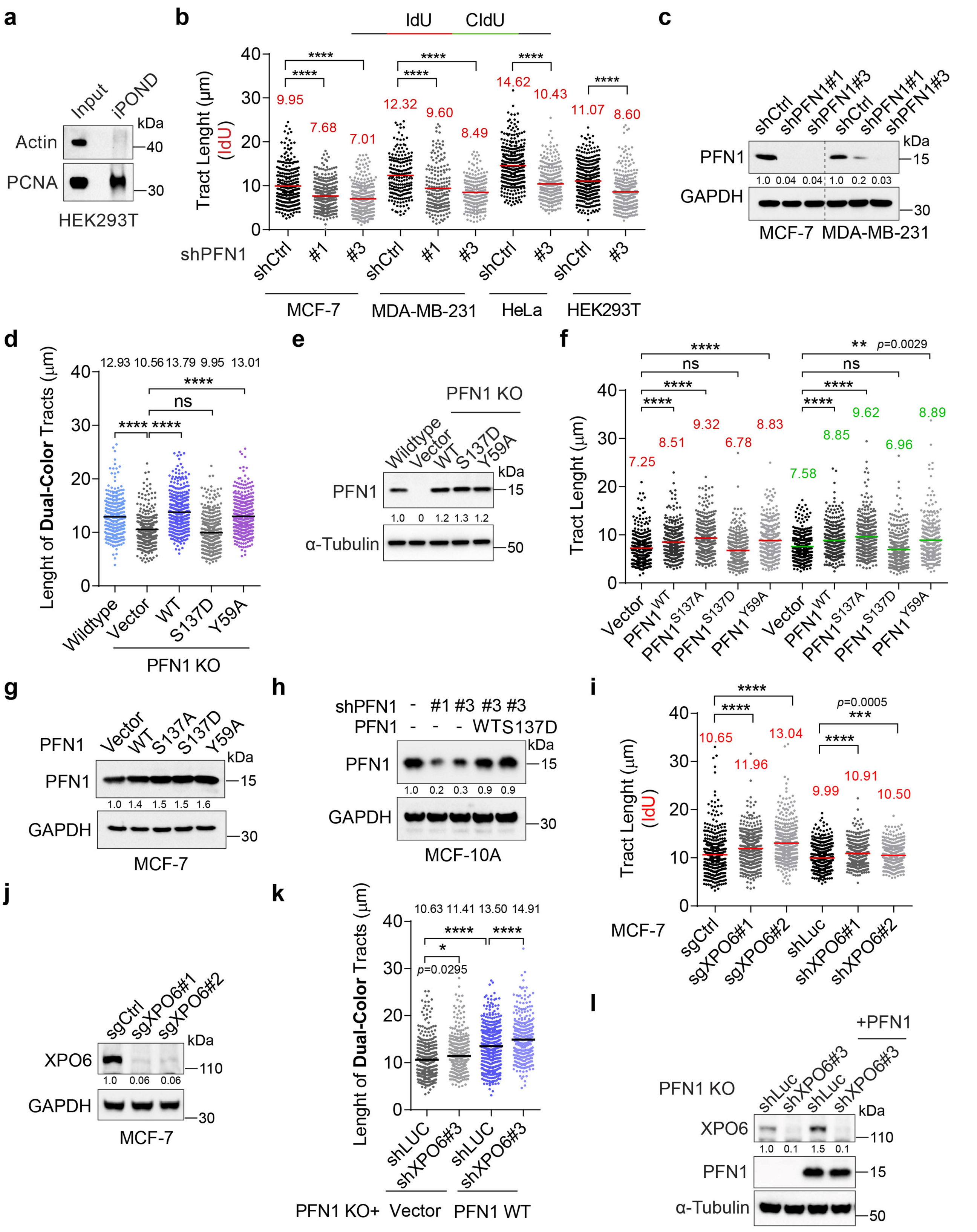

Supplementary Fig. 1 PFN1 is important for unperturbed DNA replication, related to Fig. 1
a Actin is not detected on nascent DNA by iPOND assay. Same iPOND and input samples from main Fig.1**a** were analyzed by anti-actin Western blot. **b** DNA fiber assay in control and PFN1 knockdown MCF-7, MDA-MB-231, HeLa and HEK293T cells. **c** Western blots confirming PFN1 knockdown in different cell lines used in (**b**). **d** DNA fiber assay in wild type or PFN1-null mouse chondrocyte cells infected with vector control or different PFN1 proteins. Total tract lengths of dual-color DNA fibers were analyzed. **e** Western blots showing PFN1 levels in mouse chondrocytes used in (**d**). **f** DNA fiber assay in MCF-7 cells stably infected with vector or different PFN1 proteins. **g** Western blots showing PFN1 levels in the MCF-7 cells used in (**f**). **h** Western blots confirming PFN1 knockdown with or without rescue in MCF-10A cells used for PLA and DNA fiber assays. **i** DNA fiber assay in MCF-7 cells with XPO6 knockout (sgRNA) or knockdown (shRNA). **j** Western blots confirming XPO6 knockout in MCF-7 cells for (**i**). **k** DNA fiber assay in PFN1-null mouse chondrocyte cells reconstituted with vector or wild type PFN1 which were subsequently infected with shLUC or shXPO6#3. The total tract lengths of dual-color fibers were analyzed. **l** Western blots confirming PFN1 re-expression and XPO6 knockdown in PFN1-null chondrocytes used in (**k**). In (**b, d, f, i, k**), around 300 DNA fibers were analyzed per condition. Kruskal-Wallis test with Dunnett's multiple comparisons was used. *, $p < 0.05$; **, $p<0.01$; ***, $p < 0.001$; ****, $p < 0.0001$; ns, not significant. Results were confirmed by n=3 independent experiments. Western blots in (**c**, **e**, **g**, **h**, **j** and **l**) were quantified by densitometry, and bands of interest were shown as relative values after normalizing to GAPDH or tubulin. Source data are provided as a Source Data file.

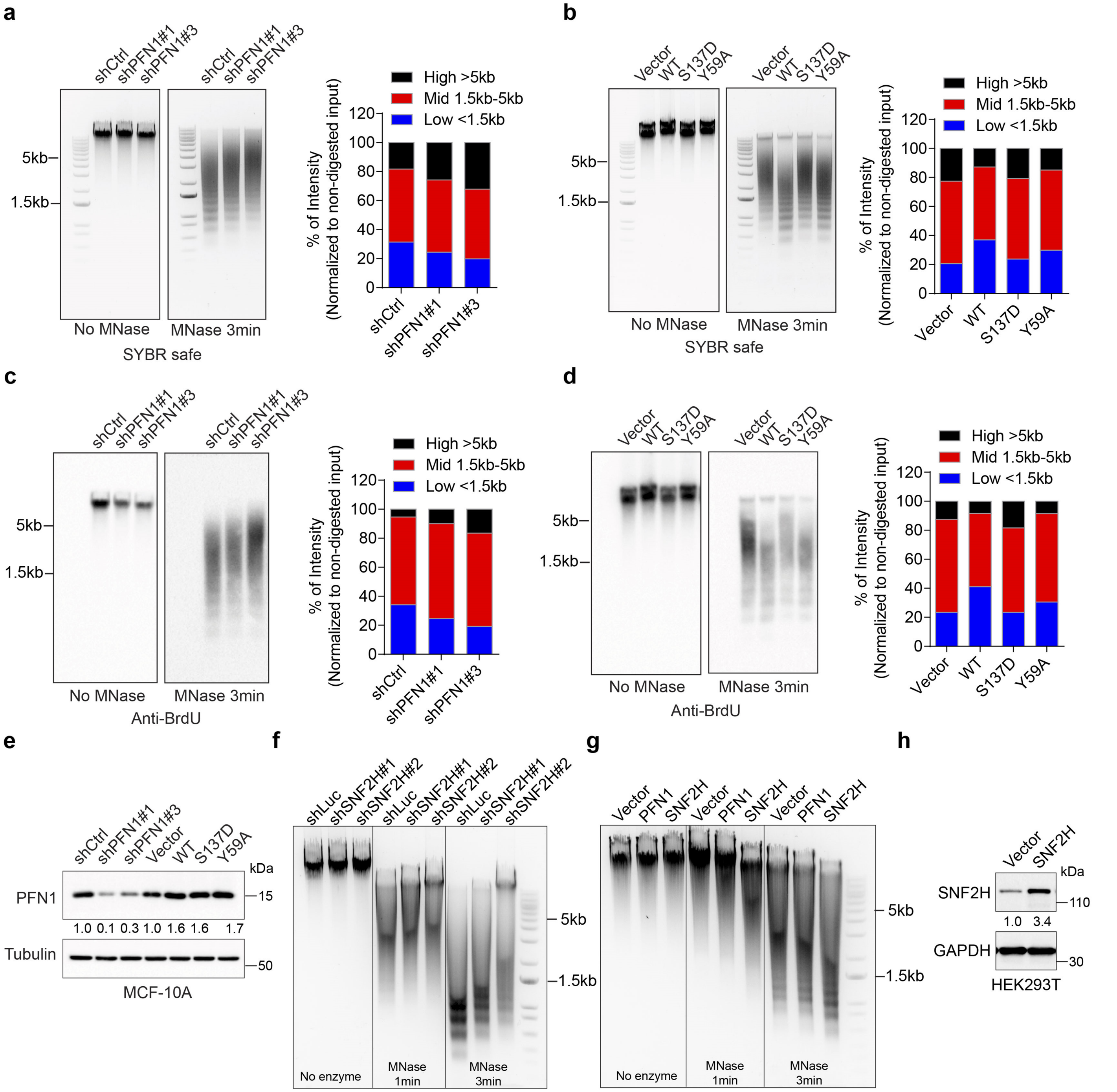

Supplementary Fig. 2 PFN1 and SNF2H function together to promote DNA replication, related to Fig. 2
a-b Micrococcal nuclease (MNase) digestion of chromatin samples stained with SYBR safe from control and PFN1 knockdown (**a**) or overexpression (**b**) MCF-10A cells. **c-d** Anti-BrdU blotting of MNase-digested chromatin samples from (**a-b**) using Southern-Western blot. For (**a-d**), each gel image or blot was divided into high (>5kb), medium (1.5-5kb), and low (<1.5kb) molecular weight regions, and the relative intensity of each region was calculated as the percentage of total intensity in the entire lane. Equal numbers of cells were used for all experimental groups, and same amounts of chromatin samples were digested by MNase as the undigested input controls. Similar phenotypes were observed in at least n=3 independent experiments. **e** Western blots confirming PFN1 knockdown and overexpression in MCF-10A cells for (**a-d**). Densitometry was performed, and relative levels of PFN1 were shown after normalizing to tubulin. **f-g** MNase digestion and quantitative analysis of chromatin DNA stained by SYBR safe using HEK293T cells with SNF2H knockdown (**f**) and overexpression (**g**) as in (**a-b**). Similar phenotypes were observed in n=3 independent experiments. **h** Western blot confirmation of SNF2H overexpression in HEK293T cells used for (**g**) and DNA fiber assay. Densitometry was performed, and relative levels of SNF2H were shown after normalizing to GADPH. Source data are provided as a Source Data file.

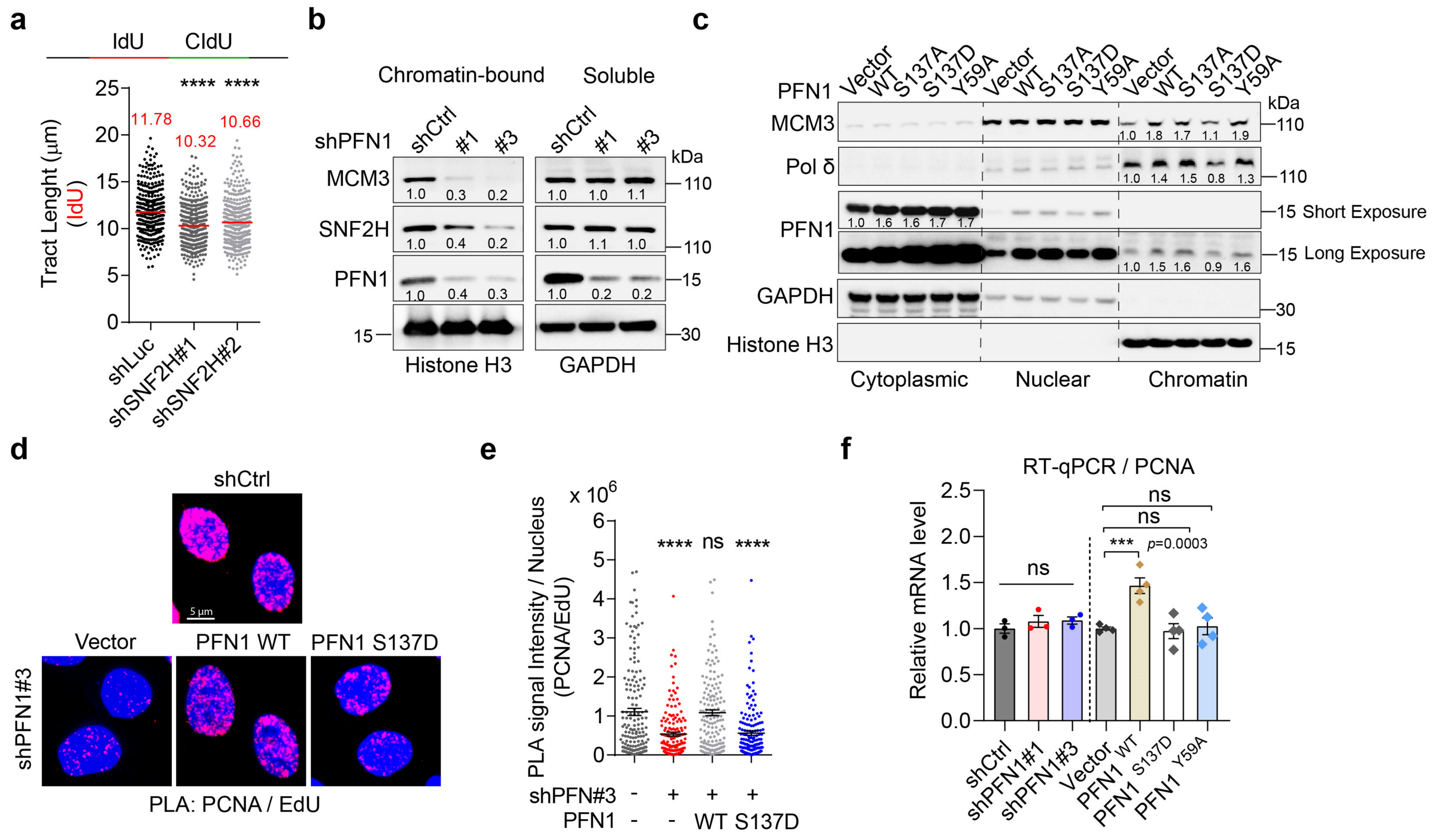

Supplementary Fig. 3 PFN1 and SNF2H function together to promote DNA replication, related to Fig. 2
a DNA fiber analysis in control and SNF2H knockdown MCF-7 cells. For each condition, at least 300 DNA fibers were analyzed. P values were based on the Kruskal-Wallis test with Dunnett's multiple comparisons. **b** Western blot analysis of insoluble and soluble fractions of RIPA-lysed MCF-10A cells with and without PFN1 knockdown. Densitometry was performed, and relative levels of MCM3, PCNA, and PFN1 were shown after normalizing to GAPDH for soluble fractions and H3 for chromatin fractions. **c** Subcellular fractionation using control and PFN1-overexpressing MCF-10A cells. Densitometry was performed, and relative levels of MCM3, Pol δ, and PFN1 were shown after normalizing to GAPDH for soluble fractions and H3 for chromatin fractions. **d**-**e** PLA between endogenous PCNA and biotinylated EdU in MCF-10A cells with PFN1 knockdown and rescue. Scale bar, 5 µm. PLA intensities of around 200 positive cells per condition were analyzed and shown as mean ± SEM. P values were based on the Kruskal-Wallis test with Dunnett's multiple comparisons. **f** RT-qPCR analysis of *PCNA* mRNA level in MCF-10A cells with PFN1 knockdown and overexpression. Shown are mean ± SEM of n=3 (PFN1 knockdown) and n=4 (PFN1 overexpression) independent experiments. P values were based on the One-Way ANOVA test with Dunnett's multiple comparisons. For all statistical tests, ***, $p < 0.001$; ****, $p < 0.0001$; ns, not significant. Source data are provided as a Source Data file.

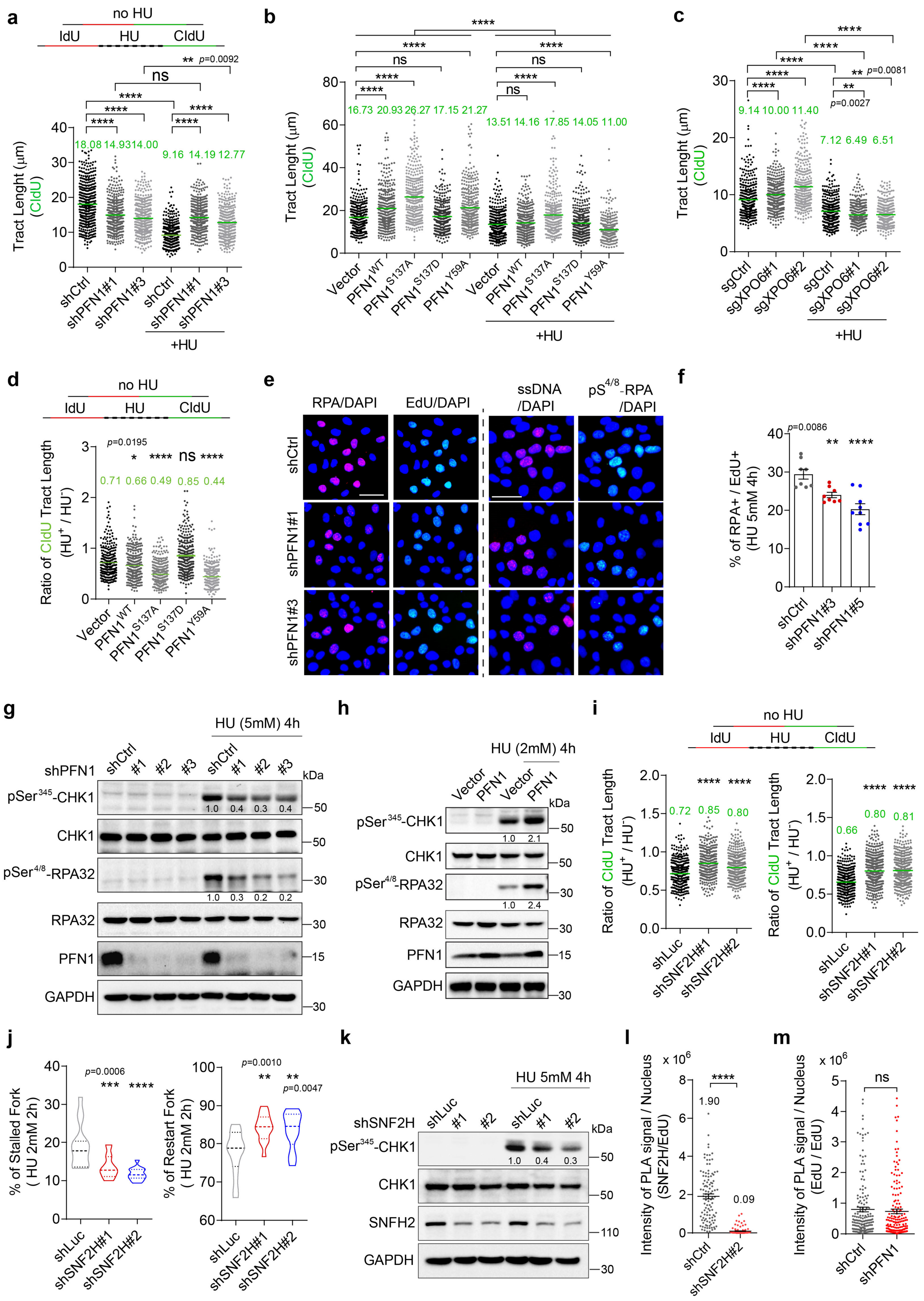

a
no HU
IdU
HU
CldU
Tract Lenght (μm) (CldU)
18.08
14.93
14.00
9.16
14.19
12.77
ns
p=0.0092
shCtrl
shPFN1#1
shPFN1#3
+HU
b
16.73
20.93
26.27
17.15
21.27
13.51
14.16
17.85
14.05
11.00
Vector
PFN1 WT
PFN1 S137A
PFN1 S137D
PFN1 Y59A
c
9.14
10.00
11.40
7.12
6.49
6.51
p=0.0081
p=0.0027
sgCtrl
sgXPO6#1
sgXPO6#2
d
Ratio of CldU Tract Length (HU+ / HU-)
p=0.0195
0.71
0.66
0.49
0.85
0.44
e
RPA/DAPI
EdU/DAPI
ssDNA /DAPI
pS4/8-RPA /DAPI
shCtrl
shPFN1#1
shPFN1#3
f
% of RPA+ / EdU+ (HU 5mM 4h)
p=0.0086
shCtrl
shPFN1#3
shPFN1#5
g
HU (5mM) 4h
shPFN1
shCtrl
#1
#2
#3
kDa
pSer345-CHK1
CHK1
pSer4/8-RPA32
RPA32
PFN1
GAPDH
1.0 0.4 0.3 0.4
1.0 0.3 0.2 0.2
h
HU (2mM) 4h
Vector
PFN1
1.0 2.1
1.0 2.4
i
0.72
0.85
0.80
0.66
0.80
0.81
shLuc
shSNF2H#1
shSNF2H#2
j
% of Stalled Fork (HU 2mM 2h)
% of Restart Fork (HU 2mM 2h)
p=0.0006
p=0.0010
p=0.0047
k
HU 5mM 4h
shSNF2H
shLuc
SNFH2
1.0 0.4 0.3
l
Intensity of PLA signal / Nucleus (SNF2H/EdU)
x 10^6
1.90
0.09
shCtrl
shSNF2H#2
m
Intensity of PLA signal / Nucleus (EdU / EdU)
shPFN1

Supplementary Fig. 4. PFN1 increases the stalling of stressed DNA replication forks, related to Fig. 3
a-**c** Raw CldU tract lengths corresponding to Fig. 3**c**-**e**. **d** Ratios of CldU tract lengths in dual-labeled (IdU 1st, CldU 2nd) control and PFN1-overexpressing MCF-7 cells with and without HU treatment in the middle, as described in Fig. 3**c**-**e**. **e** Representative images of HU-treated MCF-10A cells co-stained for total RPA32 and EdU label, pSer$^{4/8}$-RPA32 (for Fig. 3**f**), and ssDNA (native anti-BrdU staining, for Fig. 3**g**). Scale bar, 50 µm. **f** Percentages of RPA32/EdU double-positive MCF-10A cells as shown in Supplementary Fig. 3**e**. Data are mean ± SEM of one representative experiment containing around 1000 cells in each group. **g**-**h** Extracts of MCF-10A cells with PFN1 knockdown and overexpression were analyzed by Western blot after HU exposure. Phospho-proteins were quantified by densitometry and shown as relative values after normalizing to GAPDH. **i** Ratios of CldU tract lengths in dual-labeled control and SNF2H knockdown MCF-10A (left) and MCF-7 (right) cells with and without HU treatment (4mM, 2hr) in the middle, as described in (**d**). For (**a, b, c, d, i**), at least 300 fibers were analyzed per condition. **j** Percentages of stalled and restarted forks in HU-treated MCF-7 cells expressing shLuc or shSNF2H, as described in Fig. 3**a**, 3**b**, and 3**i**. Shown are mean ± SEM of n=2 independent experiments with around 1000 fibers per group. **k** Extracts of control and SNF2H knockdown MCF-10A cells were analyzed by Western blot after HU exposure. Relative levels of pSer^{345}-CHK1 were shown after normalizing to GAPDH. **l** Control PLA showing specificity of the SNF2H antibody. EdU-SNF2H PLA signals were abolished in MCF-10A cells upon SNF2H knockdown. **m** Control EdU-EdU PLA showing minimal changes in MCF-10A cells upon PFN1 knockdown. In (**l, m**), PLA intensities of around 200 cells per condition were analyzed and shown as mean ± SEM. In (**a**-**d** and **i**), statistical significance was calculated using Kruskal-Wallis test with Dunnett's multiple comparisons. In (**f** and **j**), One-Way ANOVA test with Dunnett's multiple comparisons was performed. In (**l** and **m**), two-sided Mann Whitney test was performed. For all statistical tests, *, p<0.05; **, p<0.01; ***, p<0.001; ****, p<0.0001; ns, not significant. Source data are provided as a Source Data file.

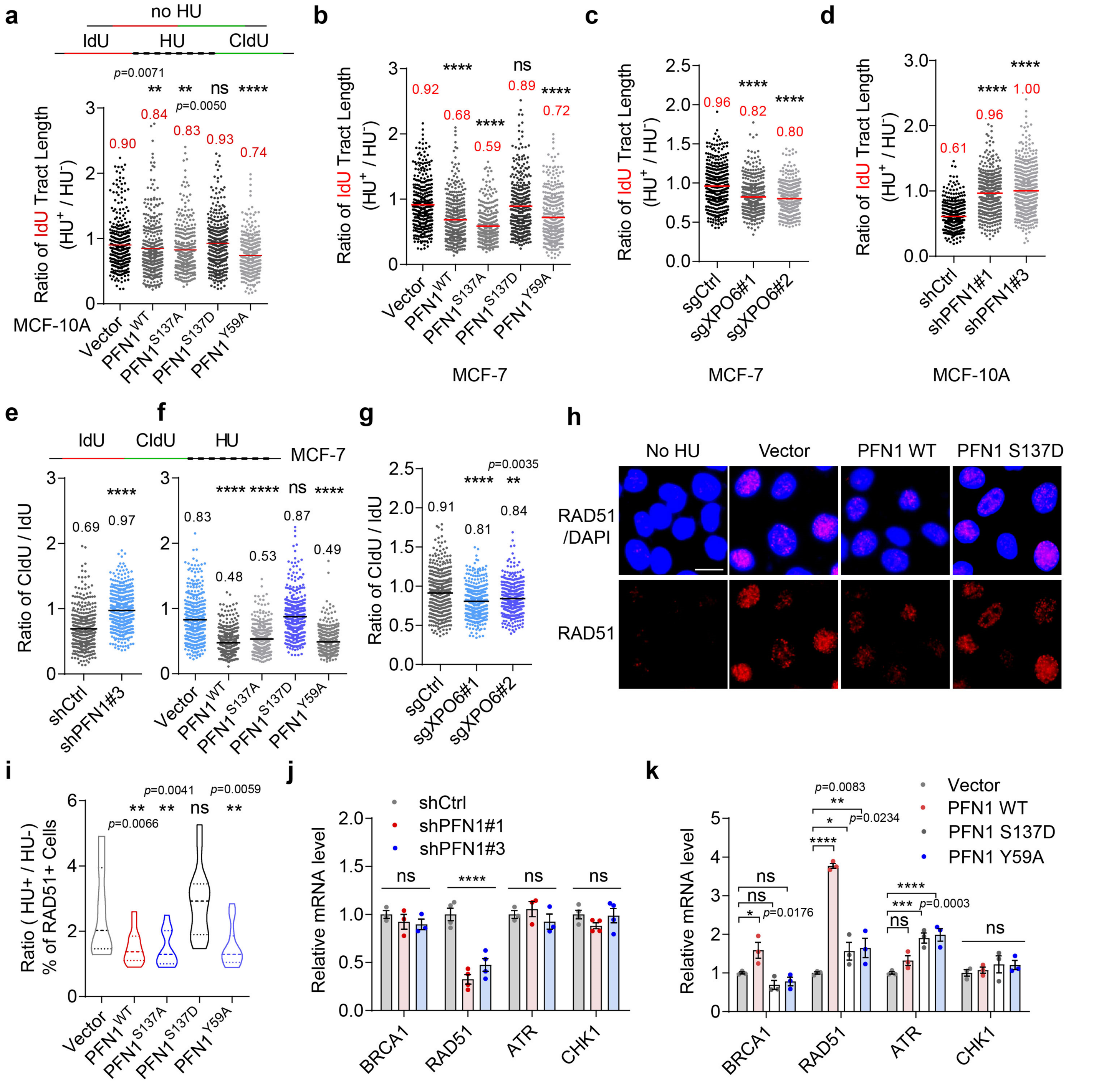

Supplementary Fig. 5 PFN1 decreases the stability of stressed DNA replication forks, related to Fig. 4
a-**d** Ratios of IdU tract lengths in dual-labeled (IdU 1st, CldU 2nd) MCF-10A (**a** and **d**) or MCF-7 (**b** and **c**) cells with and without 2hr HU (2mM in **a**-**c**; 4mM in **d**) in the middle. CldU tracts of the same experiments were analyzed in Fig. 3**c**-**e** and Supplementary Fig. 3**d**. **e**-**g** CldU/IdU ratios in MCF-7 cells (PFN1 knockdown in **e**, PFN1 overexpression in **f**, and XPO6 knockout in **g**) sequentially labeled with IdU and CldU, and treated for 2hr with HU (4mM in **e** and 2mM for **f**-**g**). In (**a**-**g**), at least 300 fibers per condition were analyzed and p values were determined by Kruskal-Wallis test with Dunnett's multiple comparisons. **h**-**i** RAD51 foci staining in control and PFN1-overexpressing MCF-10A cells treated or not with HU (4 mM, 2hr). Scale bar, 20 µm. Percentages of RAD51 foci-positive cells (foci number > 5) were quantified under HU and mock-treated conditions and expressed as ratios. Data represent mean ± SEM from around 2000 cells analyzed in each group. P values were based on One-Way ANOVA and Dunnett's multiple comparisons test. Similar effects were observed in three independent experiments. **j**-**k** RT-qPCR analysis of mRNA levels of selected genes in MCF-10A cells with PFN1 knockdown and overexpression as in Supplementary Fig. 3**f**. Shown are mean ± SEM of n=3 (BRCA1 and ATR in **j**, and all genes in **k**) or n=4 (RAD51 and CHK1 in **j**) independent experiments. Two-Way ANOVA test with Dunnett's multiple

comparisons was performed. For all statistical tests, *, p<0.05; **, p<0.01; ***, p<0.001; ****, p<0.0001; ns, not significant. Source data are provided as a Source Data file.

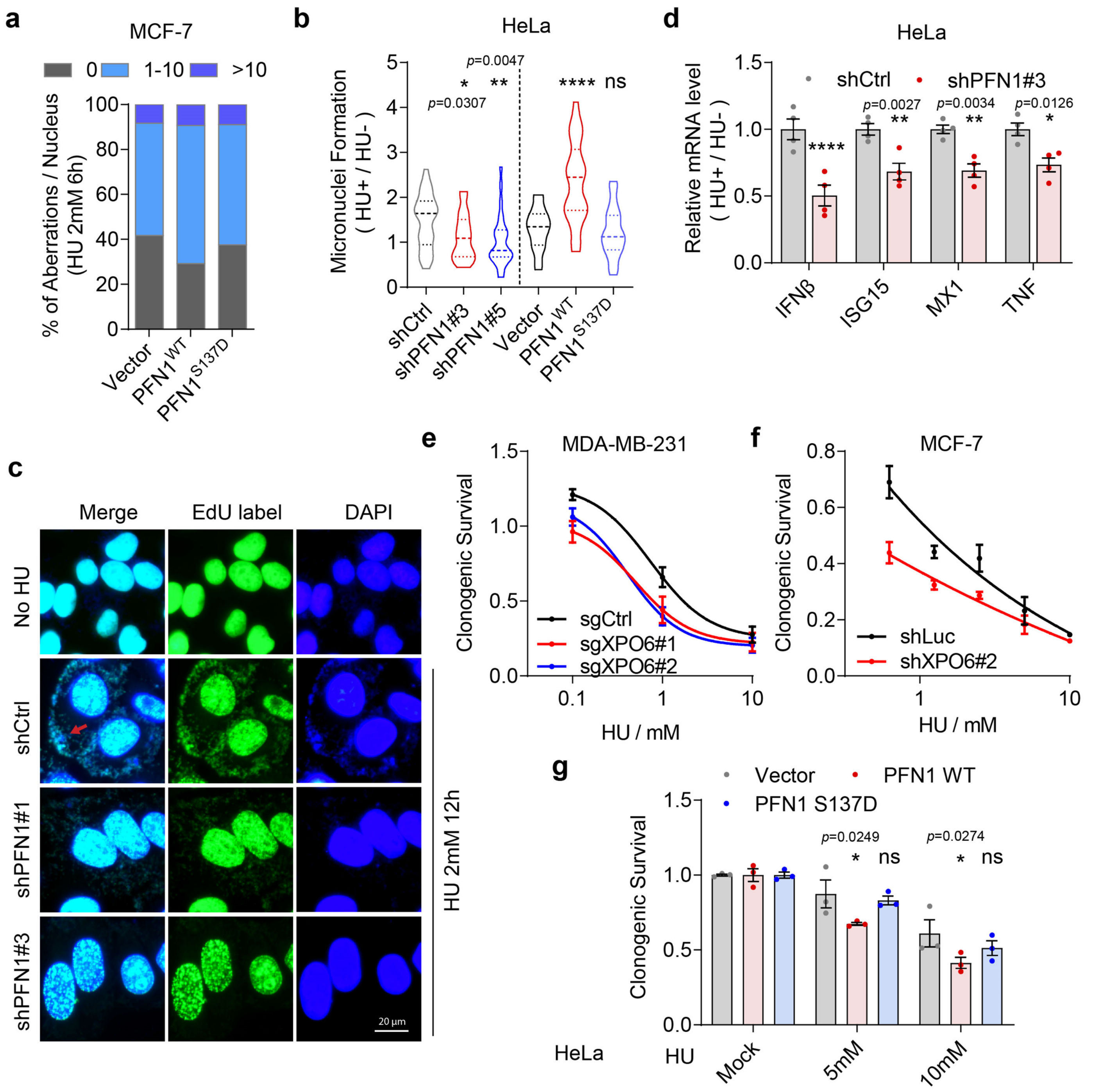

Supplementary Fig. 6 PFN1 increases genome instability during replication stress, related to Fig. 5
a Metaphase spreads using control and PFN1-overexpressing MCF-7 cells after HU exposure (2mM, 6hr). Around 100 metaphases from n=2 independent experiments were analyzed for each sample. **b** Micronuclei analysis in PFN1 knockdown or overexpression HeLa cells after HU (2mM, 6hr) treatment. Data were analyzed as in Fig. 5**e-f** with around 1000 cells in each sample. **c** Representative images of cytosolic DNA visualized by EdU staining in mock or HU-treated HeLa cells expressing control or PFN1 shRNAs (related to Figure 5**i**). Scale bar, 20 µm. **d** RT-qPCR analysis of HU-induced expression of type I interferon genes in control or PFN1 knockdown HeLa cells, as described in Fig. 5**j-k**. Shown are mean ± SEM of n=4 independent experiments. **e-g** Clonogenic assays using mock or HU-treated (6hr) MDA-MB-231 (XPO6 knockout in **e**), MCF-7 (XPO6 knockdown in **f**), and HeLa (PFN1 overexpression in **g**) cells. Shown are the mean ±

SEM of n=3 independent experiments. P values in (**b**, **d** and **g**) were based on Two-Way ANOVA analysis of Dunnett's multiple comparisons test. *, p<0.05; **, p<0.01; ****, p<0.0001; ns, not significant. Source data are provided as a Source Data file.

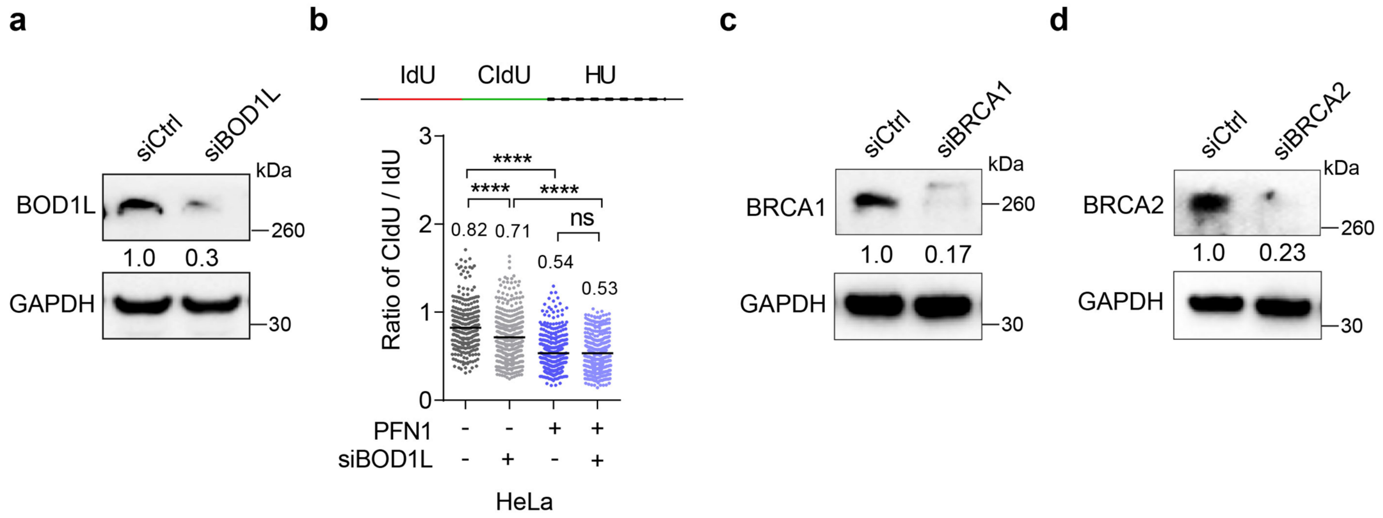

Supplementary Fig. 7 PFN1 binds BOD1L and suppresses its fork-protective activity, related to Fig. 6
a, **c** and **d** Western blot analysis of MCF-10A cell extract by RIPA lysis confirming knockdown of BOD1L, BRCA1, and BRCA2, used for DNA fiber analysis in Fig. 6**c**-**e**. Densitometry was performed for all bands, and relative levels of the silenced gene products were calculated after normalizing to GAPDH. **b** CldU/IdU ratios in dual-labeled and subsequently HU-treated (2mM, 2hr) HeLa cells overexpressing PFN1 and transfected with control or BOD1L siRNAs. At least 300 DNA fibers were analyzed per sample. P values were based on Kruskal-Wallis test with Dunnett’s multiple comparisons. ****, p < 0.0001; ns, not significant. Results were confirmed by at least n=2 independent experiments. Source data are provided as a Source Data file.

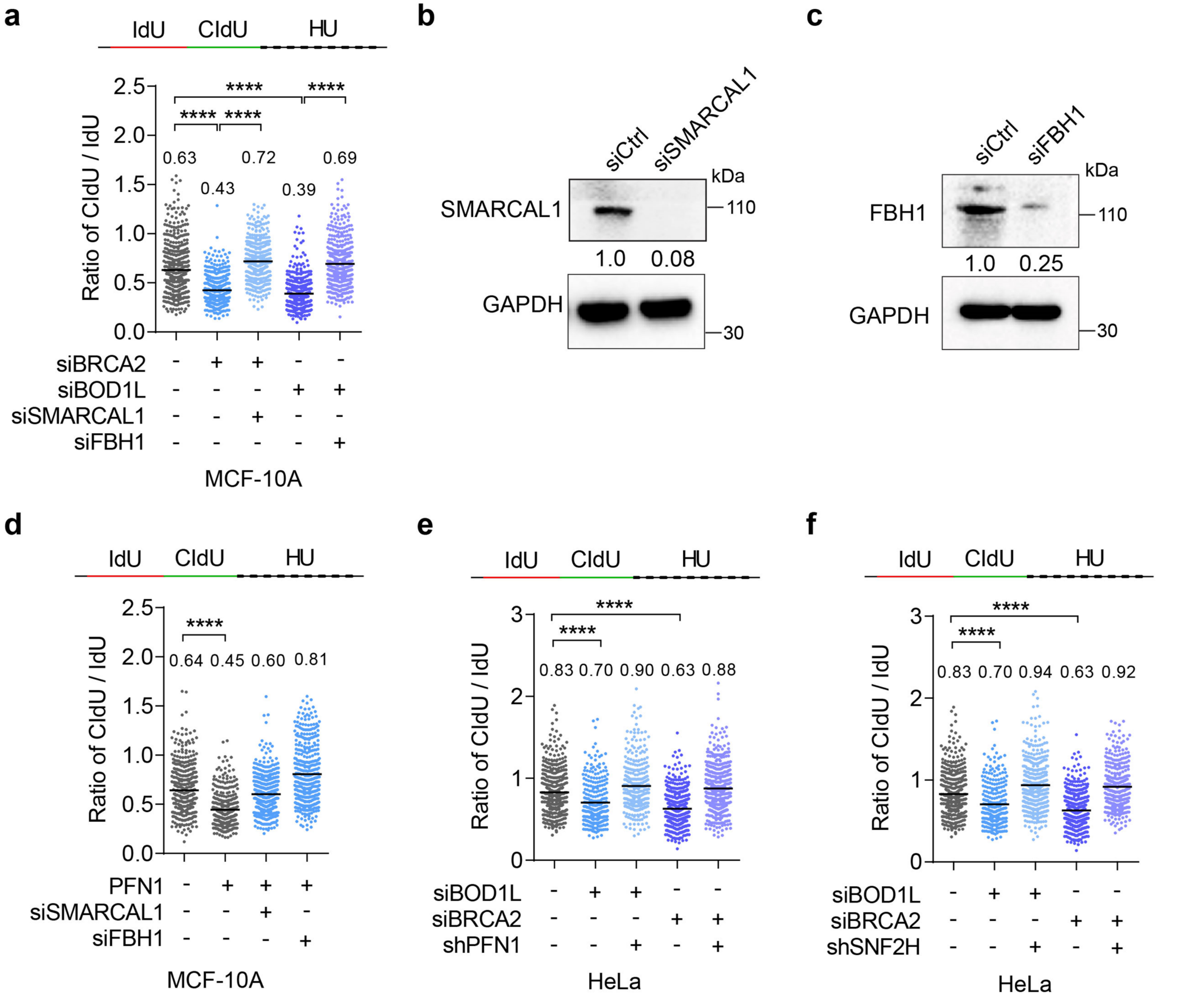

Supplementary Fig. 8 Degradation of stressed forks requires PFN1 and SNF2H, related to Fig. 7
a CldU/IdU ratios in MCF-10A cells transfected with the indicated siRNAs. **b**-**c** Western blot analysis of MCF-10A cell extracts confirming knockdown of SMARCAL1 and FBH1. Densitometry was performed, and relative levels of SMARCAL1 and FBH1 were calculated after normalization to GAPDH. **d** CldU/IdU ratios in control or PFN1-overexpressing MCF-10A cells transfected with indicated siRNAs. **e**-**f** CldU/IdU ratios in PFN1 or SNF2H knockdown HeLa cells transfected with indicated siRNAs. All cells in (**a, d**-**f**) were dual-labeled with IdU-CldU and subsequently treated with 4mM HU for 2hr, and at least 300 DNA fibers were analyzed per sample. Statistical significance was determined by the Kruskal-Wallis test with Dunnett's multiple comparisons. ****, $p < 0.0001$; ns, not significant. Results were confirmed by at least two independent experiments. Source data are provided as a Source Data file.

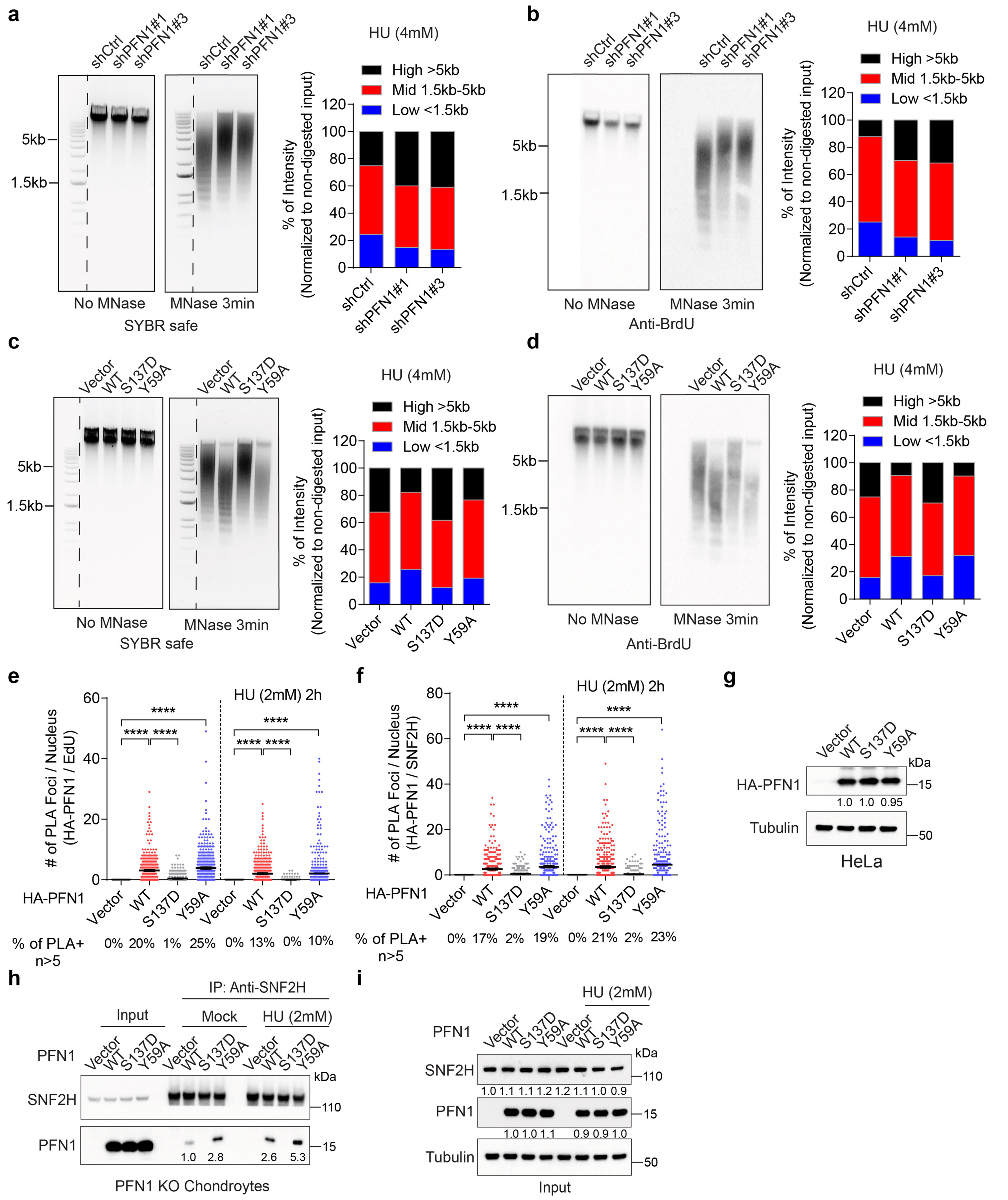

Supplementary Fig. 9 Degradation of stressed forks requires PFN1 and SNF2H, related to Fig. 7
a and **c** SYBR safe staining of micrococcal nuclease (MNase)-digested chromatin samples from control and PFN1 knockdown (**a**) or overexpression (**c**) MCF-10A cells pulsed with BrdU (10μM, 30min) followed by HU treatment (4mM,

2hr). **b** and **d** Anti-BrdU blotting of MNase-digested chromatin samples from (**a** and **c**). The dashed lines separating the DNA ladders and chromatin samples served to indicate that they were analyzed on the same gel/blot, but had to be cropped and put together since they were not immediately adjacent (see original blot in source data file). Densitometry and data quantification were performed as in Supplementary Fig. 1**a**-**d**. Similar phenotypes were observed in more than n=3 independent experiments. **e** HA-PFN1-EdU PLA in transfected HeLa cells (as in Fig. 1**c**) with or without HU exposure (2mM, 2hr). **f** PLA between HA-PFN1 and endogenous SNF2H in transfected HeLa cells (as in Fig. 2**f**) with or without HU exposure (2mM, 2hr). Quantification in (**e**-**f**) represents PLA foci number per nucleus out of around 500 cells per condition and data were shown as mean ± SEM. Statistical significance was determined by the Kruskal-Wallis test with Dunnett's multiple comparisons, ****, $p < 0.0001$. Percentages of PLA-positive cells (>5 foci per nucleus) are shown on the bottom. **g** Western blot analysis of transfected HeLa cell extracts (used for Fig. 1**c**, 2**f**, 6**b**, and Supplementary Fig. 9**e**-**f**) showing similar expression levels of wild type and mutant HA-PFN1. **h** Anti-SNF2H pulldown using PFN1-null mouse chondrocyte cells stably infected with vector or different untagged PFN1 proteins (WT vs. mutants). Cells were treated or not with HU (2mM, 2hr) before harvesting. **i** Western blot analysis of the input lysates of the mouse chondrocytes cells used for anti-SNF2H pulldown in (**h**), which showed no effect of HU on the total expression levels of SNF2H and PFN1. For (**g** and **i**), relative levels of HA-PFN1, SNF2H, and PFN1 were shown after normalization to tubulin. In (**h**), PFN1 levels (co-IPed) were normalized over SNF2H (IPed). Source data are provided as a Source Data file.

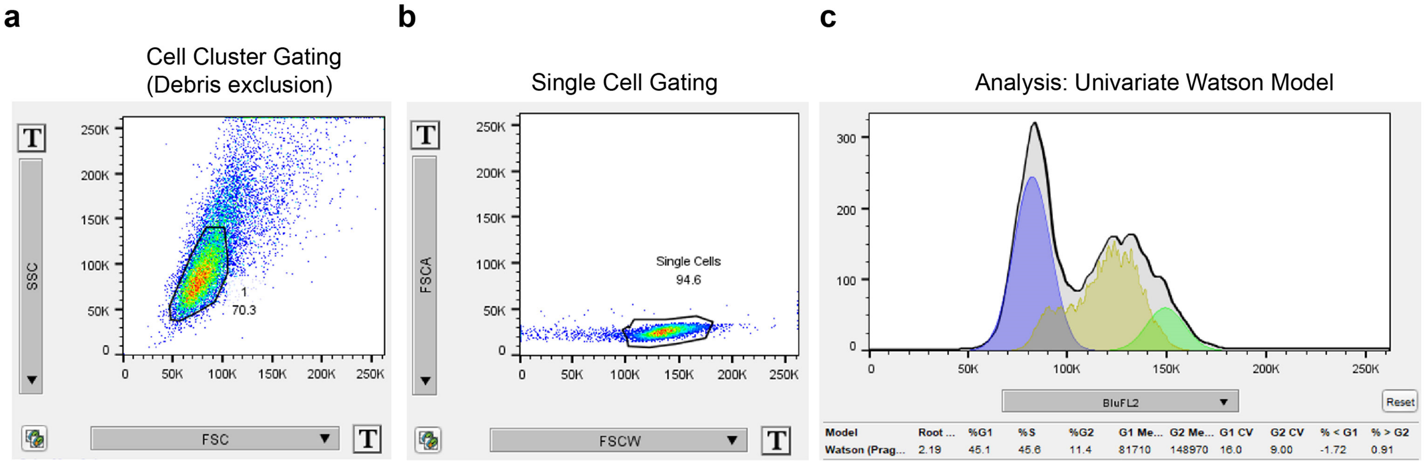

Supplementary Fig. 10 Gating strategies and analysis model for Fig. 1d. **a** Gating of cell cluster to exclude cell debris. **b** Single cell gating. **c** Cell cycle analysis using the univariate Watson model. Shown example is from shPFN1#1 MCF-10A cells which were synchronized and released for 4h. Same gating strategies and analysis model were applied to all the samples in Fig. 1**d**.
