# Supplementary material for: Profilin-1 regulates DNA replication forks in a context-dependent fashion by interacting with SNF2H and BOD1L
Source: Nat Commun. 2022 Nov 1;13:6531. doi: 10.1038/s41467-022-34310-9 (PMC9626489; doi:10.1038/s41467-022-34310-9)
Supplement: Supplementary file 2 — Reporting Summary [file 41467_2022_34310_MOESM2_ESM.pdf]

nature portfolio

Corresponding author(s): Jieya Shao

Last updated by author(s): Oct 6, 2022

Reporting Summary

Nature Portfolio wishes to improve the reproducibility of the work that we publish. This form provides structure for consistency and transparency in reporting. For further information on Nature Portfolio policies, see our Editorial Policies and the Editorial Policy Checklist.

Statistics

For all statistical analyses, confirm that the following items are present in the figure legend, table legend, main text, or Methods section.

n/a	Confirmed	
☐	☒	The exact sample size (n) for each experimental group/condition, given as a discrete number and unit of measurement
☐	☒	A statement on whether measurements were taken from distinct samples or whether the same sample was measured repeatedly
☐	☒	The statistical test(s) used AND whether they are one- or two-sided<br>*Only common tests should be described solely by name; describe more complex techniques in the Methods section.*
☒	☐	A description of all covariates tested
☐	☒	A description of any assumptions or corrections, such as tests of normality and adjustment for multiple comparisons
☐	☒	A full description of the statistical parameters including central tendency (e.g. means) or other basic estimates (e.g. regression coefficient) AND variation (e.g. standard deviation) or associated estimates of uncertainty (e.g. confidence intervals)
☐	☒	For null hypothesis testing, the test statistic (e.g. F, t, r) with confidence intervals, effect sizes, degrees of freedom and P value noted<br>*Give P values as exact values whenever suitable.*
☒	☐	For Bayesian analysis, information on the choice of priors and Markov chain Monte Carlo settings
☒	☐	For hierarchical and complex designs, identification of the appropriate level for tests and full reporting of outcomes
☒	☐	Estimates of effect sizes (e.g. Cohen's d, Pearson's r), indicating how they were calculated

Our web collection on statistics for biologists contains articles on many of the points above.

Software and code

Policy information about availability of computer code

Data collection

All the immunofluorescent images were collected by epifluorescence microscope (Olympus IX70) and CellSens as the acquisition software.
The western blot and gel images were collected by Gel Doc XR imaging system (Bio-Rad)
The flow cytometry data were collected by FACScan system (BD) with a Cytek BluFL2
RT-qPCR data were collected by CFX96 Touch™ Real-Time PCR Detection System (Bio-Rad)

Data analysis

The graphs and dots plotter were analyzed by Graphpad Prism v 8.0
Data for flow cytometry were analyzed by Flowjo v 10.0
Data for DNA fiber, PLA and immunofluorescence staining were analyzed by Image J v1.51

For manuscripts utilizing custom algorithms or software that are central to the research but not yet described in published literature, software must be made available to editors and reviewers. We strongly encourage code deposition in a community repository (e.g. GitHub). See the Nature Portfolio guidelines for submitting code & software for further information.

Data

Policy information about availability of data

All manuscripts must include a data availability statement. This statement should provide the following information, where applicable:

- Accession codes, unique identifiers, or web links for publicly available datasets
- A description of any restrictions on data availability
- For clinical datasets or third party data, please ensure that the statement adheres to our policy

The authors declare that all data are available in the main manuscript and supplementary materials. Source data are provided within this paper including raw data for all bar and violin plots and unprocessed Western blots with molecular weight markers. Raw data for dot plots can be provided upon request.

Field-specific reporting

Please select the one below that is the best fit for your research. If you are not sure, read the appropriate sections before making your selection.

☒ Life sciences ☐ Behavioural & social sciences ☐ Ecological, evolutionary & environmental sciences

For a reference copy of the document with all sections, see nature.com/documents/nr-reporting-summary-flat.pdf

Life sciences study design

All studies must disclose on these points even when the disclosure is negative.

Sample size	Sample sizes of our experiments were based on well-accepted standards in the field established by published studies (some of which are referenced below). They offered sufficient statistical power for us to draw reliable conclusions with regard to the specific biological questions.<br>1. For DNA fiber assays, at least 300 fibers were analyzed in each sample and all data were confirmed by at least two independent experiments (Quinet et al, 2017, Methods Enzymol 591, 55-82; Higgs et al, 2015, Mol Cell 59, 462-477; Taglialatela et al, 2017, Mol Cell 68, 414-430).<br>2. For proximity ligation assays, at least 100 nuclei were analyzed per condition and all data were confirmed by at least two independent experiments (Higgs et al, 2018, Mol Cell 71, 25-41; Taglialatela et al, 2017, Mol Cell 68, 414-430).<br>3. For metaphase spreading assays, at least 50 metaphases were analyzed in every sample and all data were confirmed by at least two independent experiments (Lemacon et al, 2017, Nature communications 8, 860; Li et al, 2019, Mol Cell 74, 1123-1137).
Data exclusions	No data were excluded.
Replication	All data presented in the figures were confirmed by two or more independent experiments.
Randomization	Pooled cell lines instead of single cell clones were used for all of our experiments, which provided inherent randomization for downstream analyses. All images for DNA fiber, PLA, IF, and metaphase spreading assays were randomly taken without preset orders of sample processing.
Blinding	The authors were blinded to all data analyses which were prone to bias such as DNA fiber, PLA, IF, and metaphase spreading assays. No blinding was applied to assays such as FACS, iPOND, Western blots, chromatin digestion, and DNA gels since they are either automated or produce quantitative data that are largely immune to user bias. Nonetheless, all data were confirmed by biologically independent experiments to further reduce bias.

Reporting for specific materials, systems and methods

We require information from authors about some types of materials, experimental systems and methods used in many studies. Here, indicate whether each material, system or method listed is relevant to your study. If you are not sure if a list item applies to your research, read the appropriate section before selecting a response.

Materials & experimental systems

n/a	Involved in the study	
☐	☒	Antibodies
☐	☒	Eukaryotic cell lines
☒	☐	Palaeontology and archaeology
☒	☐	Animals and other organisms
☒	☐	Human research participants
☒	☐	Clinical data
☒	☐	Dual use research of concern

Methods

n/a	Involved in the study	
☒	☐	ChIP-seq
☐	☒	Flow cytometry
☒	☐	MRI-based neuroimaging

Antibodies

Antibodies used	Primary antibodies used for Western blot: rabbit anti-Pfn1 (CST, #3246), rabbit anti-SNF2H (EMD Millipore, #ABE1026), rabbit anti-BOD1L (gift from Grant S. Stewart lab), rabbit anti-RAD51 (Merck Millipore, #PC130), mouse anti-PCNA (Santa Cruz, #sc-56), mouse anti-MCM3 (Santa Cruz, #sc-390480), mouse anti-Polδ (Santa Cruz, #sc-17776), mouse anti-GAPDH (Santa Cruz, #sc-47724), rabbit anti-Histone H3 (CST, #4499), mouse anti-HA-tag (BioLegend, # MMS-101P), rabbit anti-pThr1989-ATR (CST, #30632), rabbit anti-ATR (CST, #13934), rabbit anti-pSer345-CHK1 (CST, #2348), mouse anti-CHK1 (CST, #2360), rabbit anti-pSer4/8-RPA32 (Bethyl, #A300-245A), mouse anti-RPA32 (Santa Cruz, #sc-56770), rabbit anti-γH2AX (CST, #9718), Rabbit anti-BRCA1 (Bethyl, #A301-377), Rabbit anti-BRCA2 (Bethyl, #A303-434), Rabbit anti-SMARCAL1 (CST, #44717), mouse anti-FBH1 (Santa Cruz, #sc-81563), rabbit anti-XPO6 (ThermoFisher, # PA5-31813).<br><br>Primary antibodies for immunofluorescence staining: mouse anti-RPA32 (Santa Cruz, #sc-56770), rabbit anti-pSer4/8-RPA32 (Bethyl, #A300-245A), rabbit anti-RAD51 (Merck Millipore, #PC130), mouse anti-BrdU (Becton Dickinson, #37580) and rat anti-BrdU (Novus Biologicals, #NB500169; Abcam, #ab6326).

Primary antibodies for immunoprecipitations: mouse anti-GFP (DSHB, #DSHB-GFP-12E6), mouse anti-HA-tag (BioLegend, # MMS-101P), mouse anti-SNF2H (Santa Cruz, #sc-365727) and control mouse IgG (Santa Cruz, #sc-2025).

Primary antibodies for PLA: mouse anti-Biotin (Jackson ImmunoResearch, #200-002-211), rabbit anti-Biotin (Bethyl, #A150-109A), mouse anti-HA tag (BioLegend, # MMS-101P), mouse anti-PCNA (Santa Cruz, #sc-56), rabbit anti-SNF2H (EMD Millipore, #ABE1026) and rabbit anti-BOD1L (gift from Grant S. Stewart lab).

Secondary antibodies for Western blots: horseradish peroxidase-conjugated anti-rabbit (CST, #7074) and anti-mouse (CST, #7076). Secondary antibodies for immunofluorescence are Alexa Fluor 594-conjugated goat anti-mouse IgG (H+L) (Invitrogen, #A-11032), Alexa Fluor 488 -conjugated donkey anti-rat IgG (H+L) (Invitrogen, #A-21208), Alexa Fluor 594-conjugated goat anti-rabbit IgG (H+L) (Invitrogen, A11037) and Alexa Fluor 488-conjugated goat anti-mouse IgG (H+L) (Invitrogen, #A-11029).

Validation

The following antibodies were validated by knockdown or knockout of the target proteins by us or others in this or previous papers:
1. anti-PFN1 (CST, #3246) (this paper)
2. anti-SNF2H (EMD Millipore, #ABE1026) (this paper)
3. anti-BOD1L (gift from Dr. Grant Stewart) (this paper and Higgs et al, 2015, 59, 462-477)
4. anti-BRCA1 (Bethyl, #A301-377) (this paper)
5. anti-BRCA2 (Bethyl, #A303-434) (this paper)
6. anti-FBH1 ((Santa Cruz, #sc-81563) (this paper)
7. anti-SMARCAL1 (CST, #44717) (this paper)
8. anti-XPO6 (ThermoFisher, Cat#: PA5-31813) (this paper and Zhu et al, 2021, Cell Rep, 34, 108749)
9. anti-CHK1 (CST, #2360) (validated by CST)

The following antibodies were validated as stated on suppliers websites or independently cited by published studies. These include specific detection of the proteins by Western blot at the expected molecular weights in response to specific stimuli (e.g. pATR, pCHK1, pRPA, γH2AX upon DNA damage), specific detection of the proteins by immunofluorescence staining at the expected subcellular locations or showing expected spatial patterns, ability to recognize the intended protein targets by complementary approaches (Western blot, immunoprecipitation, immunofluoresence staining) and produce similar signals as independent antibodies raised against the same protein antigens.
1. anti-RAD51 (Merck Millipore, #PC130)
2. anti-PCNA (Santa Cruz, #sc-56)
3. anti-MCM3 (Santa Cruz, #sc-390480)
4. anti-Polδ (Santa Cruz, #sc-17776)
5. anti-pSer4/8-RPA32 (Bethyl, #A300-245A)
6. anti-RPA32 (Santa Cruz, #sc-56770)
7. anti-γH2AX (CST, #9718)
8. anti-pThr1989-ATR (CST, #30632)
9. anti-ATR (CST, #13934)
10. anti-pSer345-CHK1 (CST, #2348)
11. anti-GAPDH (Santa Cruz, #sc-47724)
12. anti-Histone H3 (CST, #4499)

The following antibodies target antigens that are not endogenously expressed in cells. All have been widely used and validated by many published studies based on 1) the inability to produce signals in cells without the exogenous expression of the target antigens and 2) detection of exogenously expressed antigens at the expected molecular weights (for proteins) or subcellular localizations (small molecules).
1. anti-HA-tag (BioLegend, # MMS-101P) (validated in this paper, the supplier, and other studies)
2. anti-GFP (DSHB, #DSHB-GFP-12E6) (validated in this paper and our previous paper Zhu C, et al, 2021, Cell reports 34, 108749)
3. anti-BrdU (Becton Dickinson #37580; Novus Biologicals #NB500169; Abcam #ab6326)
4. anti-Biotin (Bethyl, #A150-109A)

Eukaryotic cell lines

Policy information about cell lines

Cell line source(s)

MCF-10A, MDA-MB-231, MCF-7, HeLa, and HEK293T cells were purchased from ATCC. Mouse chondrocytes were provided by Dr. Ralph T. Bottcher and Dr. Reinhard Fassler (Max Planck Institute of Biochemistry, Germany).

Authentication

All cell lines show their distinctive growth and morphological behaviors as expected based on information from ATCC and published studies. As such, no authentication was performed.

Mycoplasma contamination

All cell lines were tested negative for mycoplasma contamination.

Commonly misidentified lines
(See ICLAC register)

No commonly misidentified cell lines were used in the study

Flow Cytometry

Plots

Confirm that:

☒ The axis labels state the marker and fluorochrome used (e.g. CD4-FITC).

☒ The axis scales are clearly visible. Include numbers along axes only for bottom left plot of group (a 'group' is an analysis of identical markers).

☒ All plots are contour plots with outliers or pseudocolor plots.

☒ A numerical value for number of cells or percentage (with statistics) is provided.

Methodology

Sample preparation	Details are provided in the Methods section. Briefly, MCF-10A cells were synchronized by double thymidine block (18hr first block, 9hr release, 16hr second block) and released in fresh medium for the indicated lengths of time shown in Fig.1d. After release, cells were harvested by trypsinization, fixed in 70% ethanol, and permeabilized with 0.25% Triton X-100. Cells were subsequently stained with propidium iodide containing 0.1 mg/ml RNase A prior to FACS analysis.
Instrument	Digital FACScans (BD Biosciences) with 488nm laser and BluFL2 filter (Cytek)
Software	Flowjo.v10.0
Cell population abundance	30000 cells per sample were analyzed
Gating strategy	More than 70% of the cells were selected during the initial gating (SSC vs FSC) to remove cell debris. The second gating (FSC-A vs. FSC-W) was performed to select single cells which were approximately 90% of the live cells from first gating and used for cell cycle analysis. The representative images for the gating strategies are provided in supplementary Fig. 10.

☒ Tick this box to confirm that a figure exemplifying the gating strategy is provided in the Supplementary Information.
