# Supplementary material for: Profilin-1 regulates DNA replication forks in a context-dependent fashion by interacting with SNF2H and BOD1L
Source: Nat Commun. 2022 Nov 1;13:6531. doi: 10.1038/s41467-022-34310-9 (PMC9626489; doi:10.1038/s41467-022-34310-9)

Fig.1a

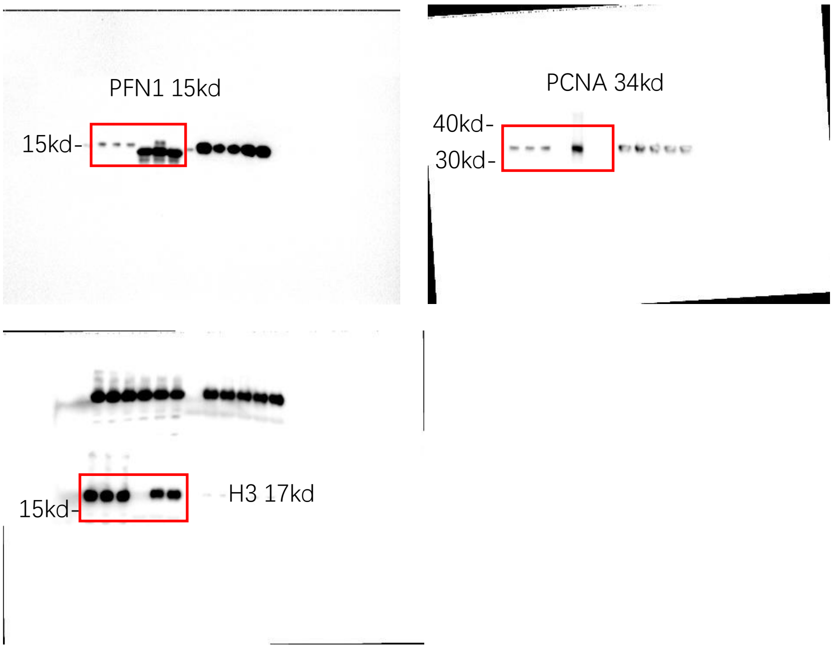

Fig. 2b

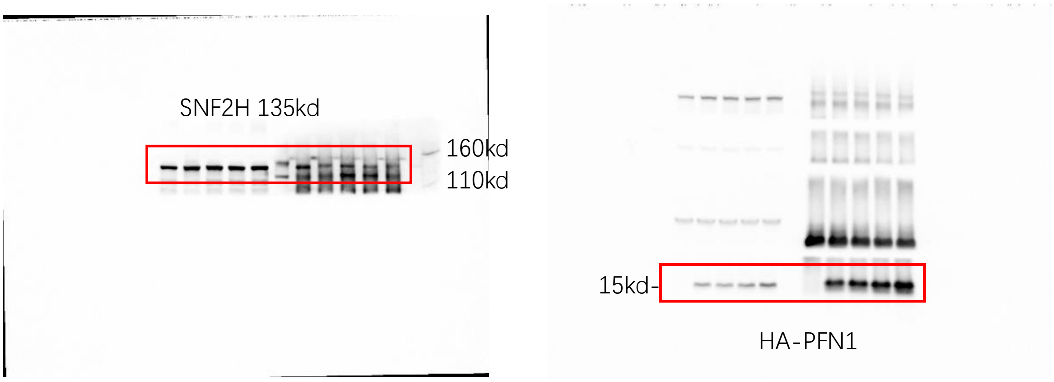

Fig.2c

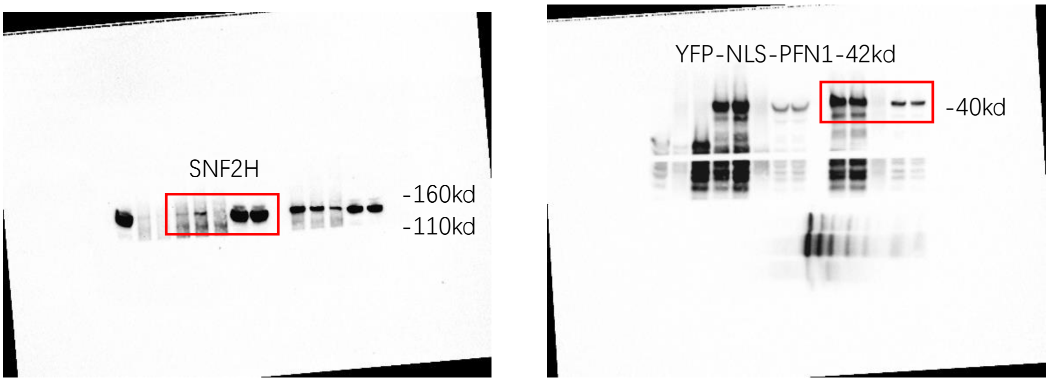

Fig. 2d

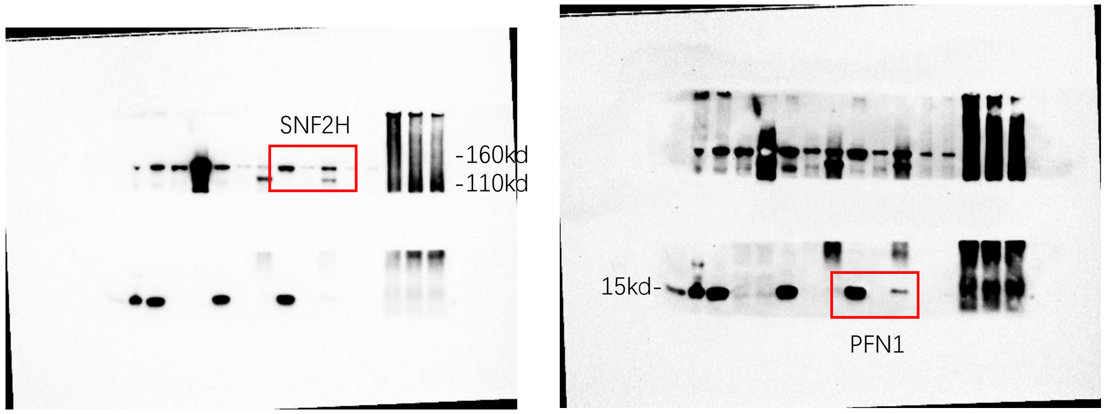
SNF2H
-160kd
-110kd
15kd-
PFN1

Fig.2m

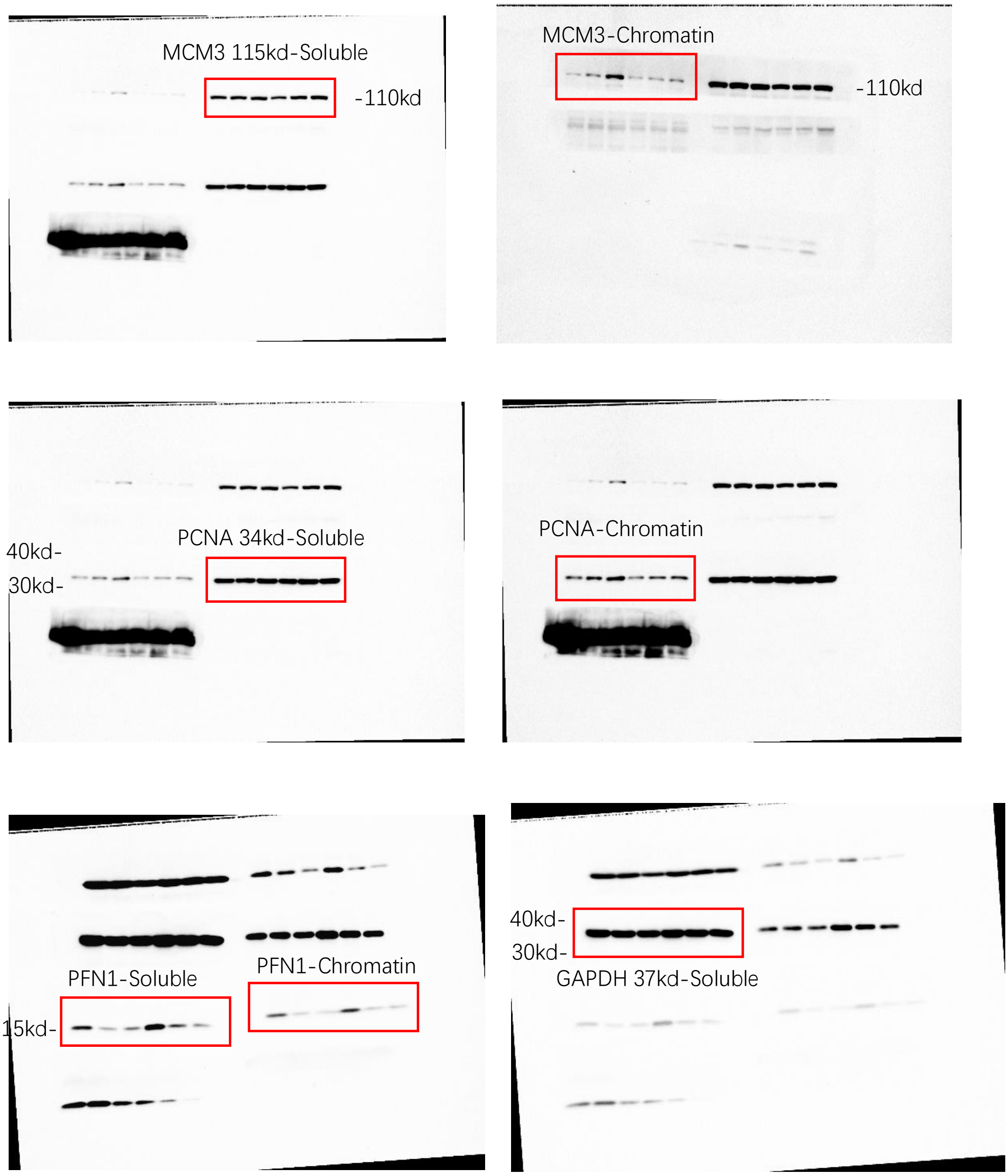
MCM3 115kd-Soluble
-110kd
MCM3-Chromatin
-110kd
PCNA 34kd-Soluble
40kd-
30kd-
PCNA-Chromatin
PFN1-Soluble
PFN1-Chromatin
15kd-
40kd-
30kd-
GAPDH 37kd-Soluble

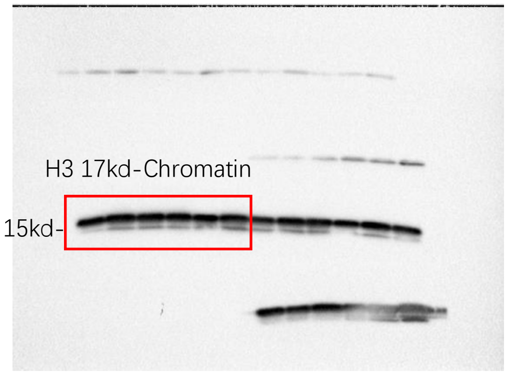
H3 17kd-Chromatin
15kd-

Fig.2n

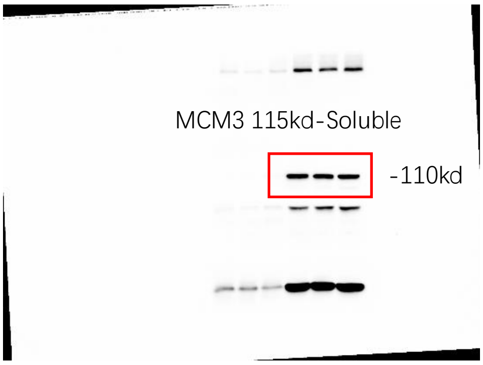
MCM3 115kd-Soluble
-110kd

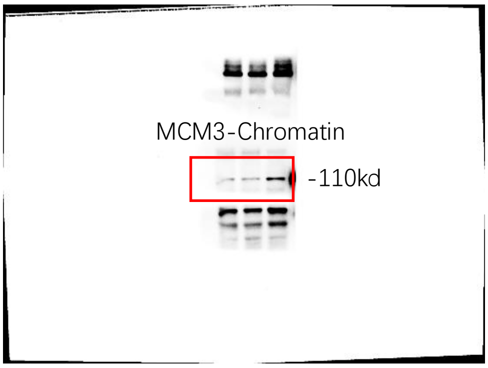
MCM3-Chromatin
-110kd

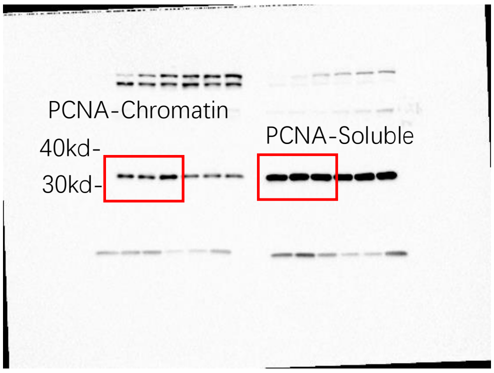
PCNA-Chromatin
PCNA-Soluble
40kd-
30kd-

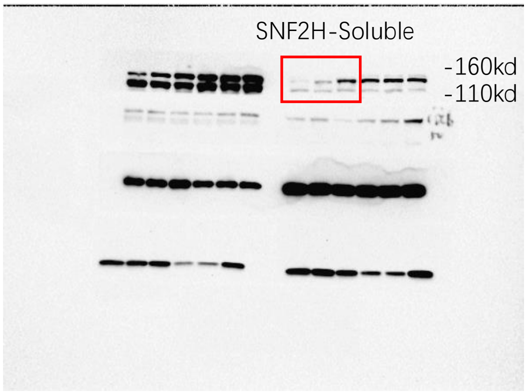
SNF2H-Soluble
-160kd
-110kd

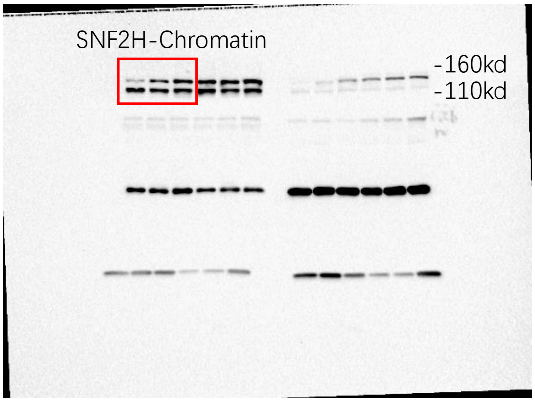
SNF2H-Chromatin
-160kd
-110kd

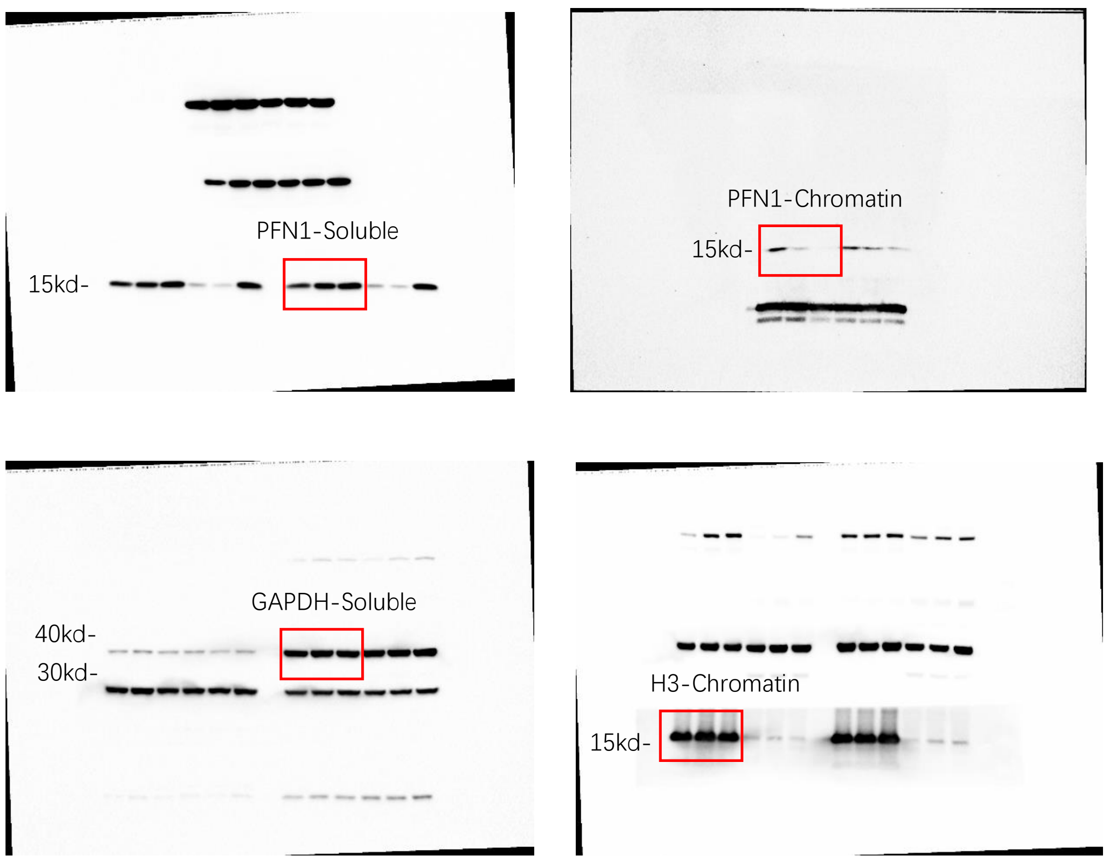
PFN1-Soluble
15kd-
PFN1-Chromatin
15kd-
GAPDH-Soluble
40kd-
30kd-
H3-Chromatin
15kd-

Fig.2o

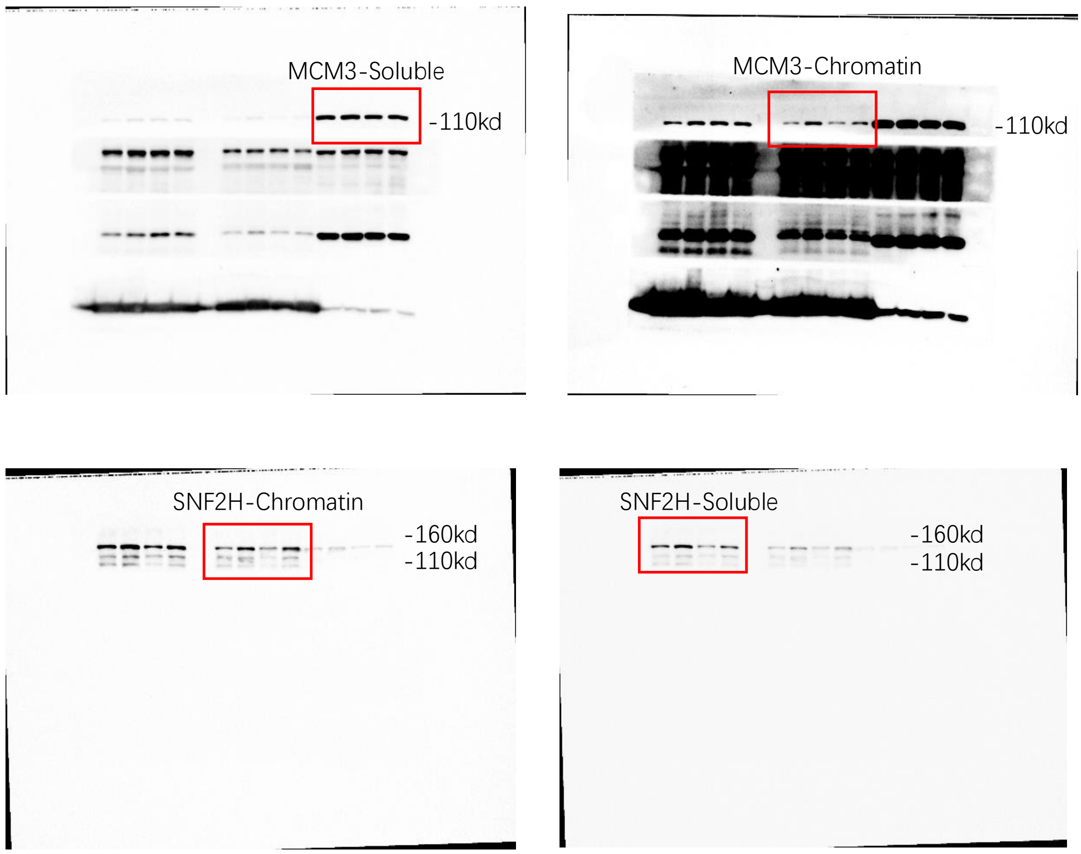
MCM3-Soluble
-110kd
MCM3-Chromatin
-110kd
SNF2H-Chromatin
-160kd
-110kd
SNF2H-Soluble
-160kd
-110kd

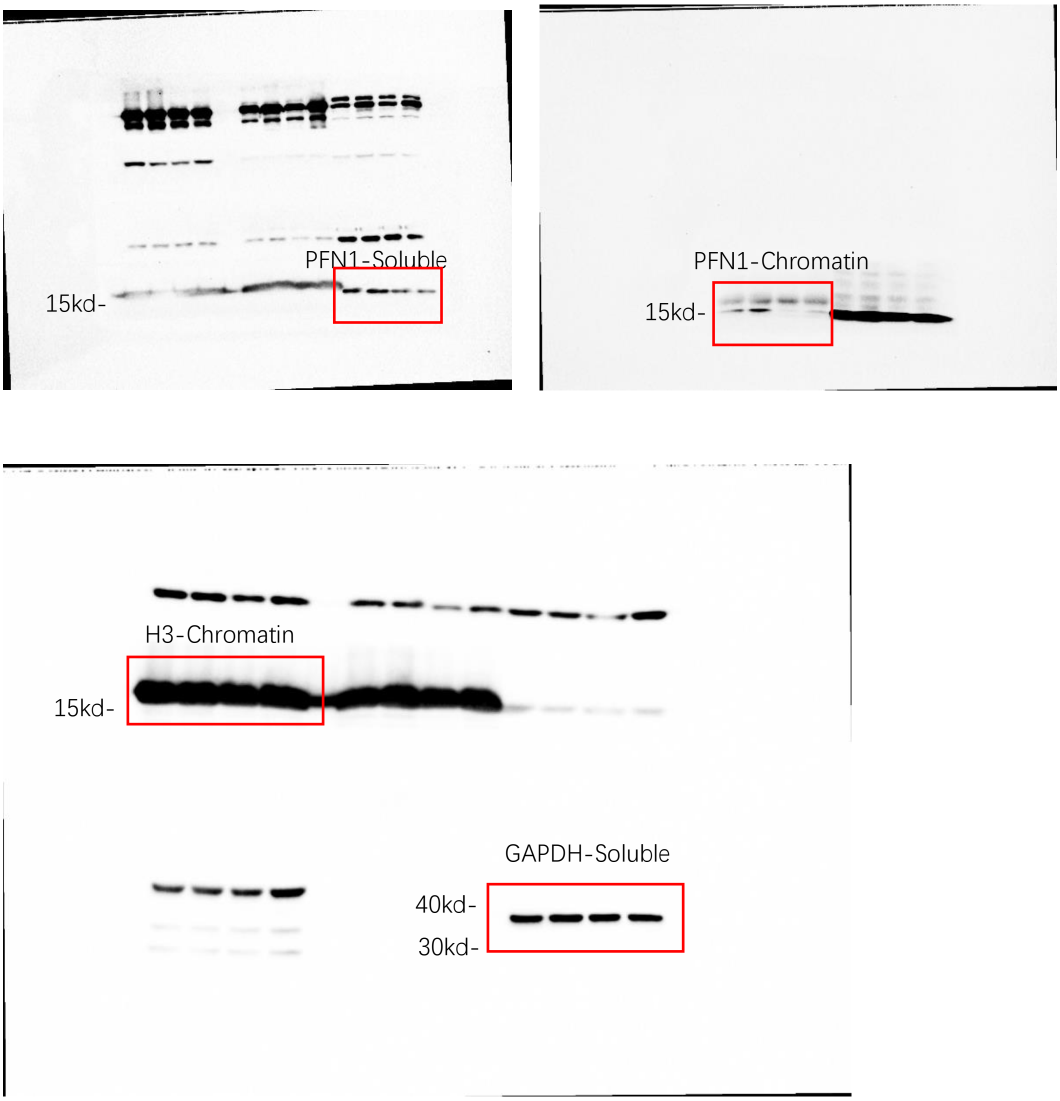
PFN1-Soluble
15kd-
PFN1-Chromatin
15kd-
H3-Chromatin
15kd-
GAPDH-Soluble
40kd-
30kd-

Fig.3h

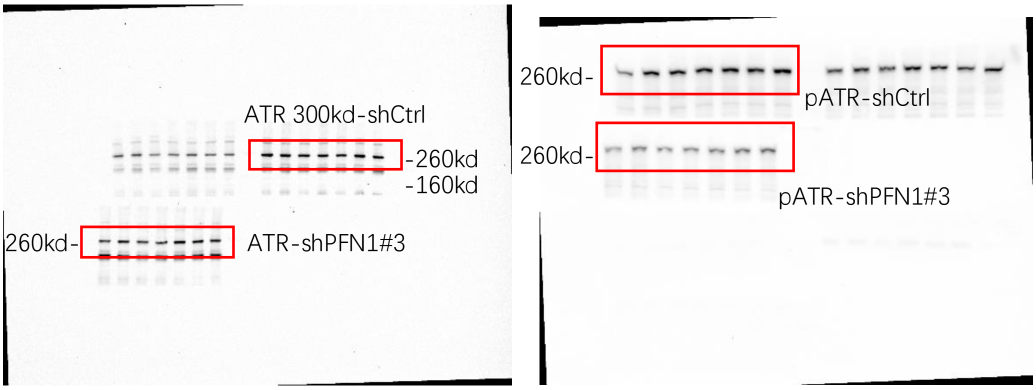
ATR 300kd-shCtrl
-260kd
-160kd
260kd-
ATR-shPFN1#3
260kd-
pATR-shCtrl
260kd-
pATR-shPFN1#3

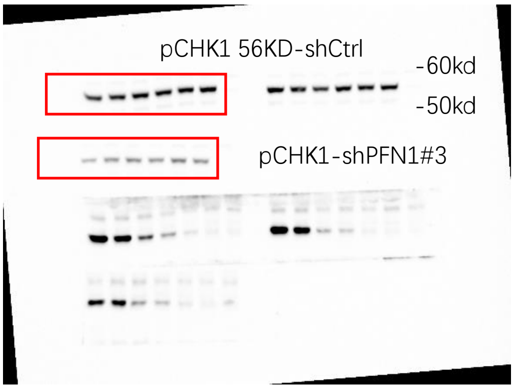
pCHK1 56KD-shCtrl
-60kd
-50kd
pCHK1-shPFN1#3

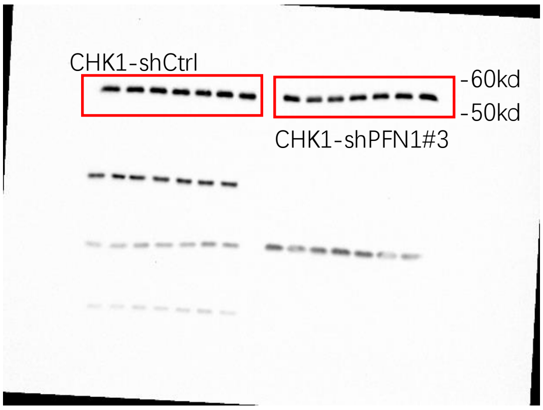
CHK1-shCtrl
-60kd
-50kd
CHK1-shPFN1#3

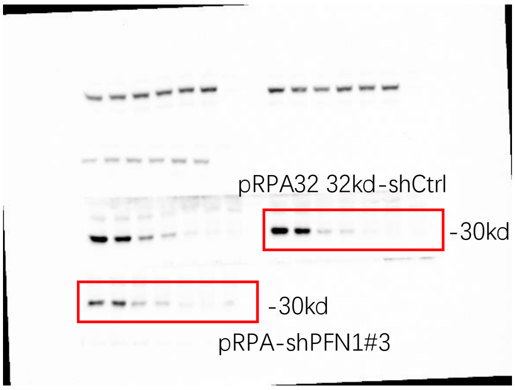
pRPA32 32kd-shCtrl
-30kd
-30kd
pRPA-shPFN1#3

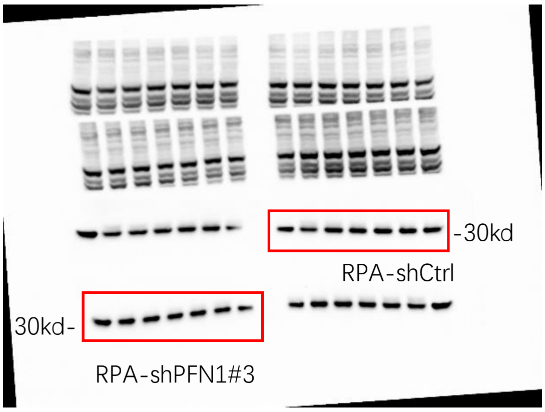
-30kd
RPA-shCtrl
30kd-
RPA-shPFN1#3

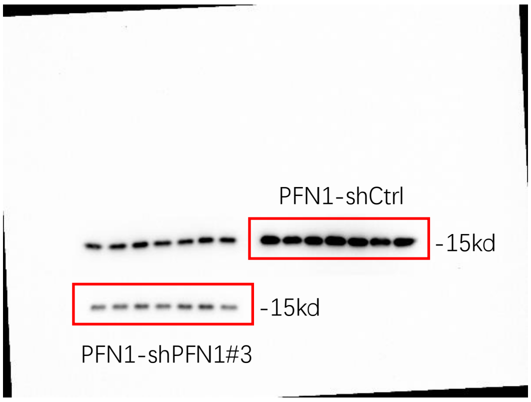
PFN1-shCtrl
-15kd
-15kd
PFN1-shPFN1#3

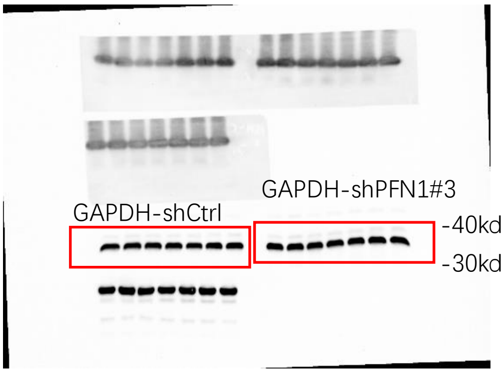
GAPDH-shPFN1#3
GAPDH-shCtrl
-40kd
-30kd

Fig.4i

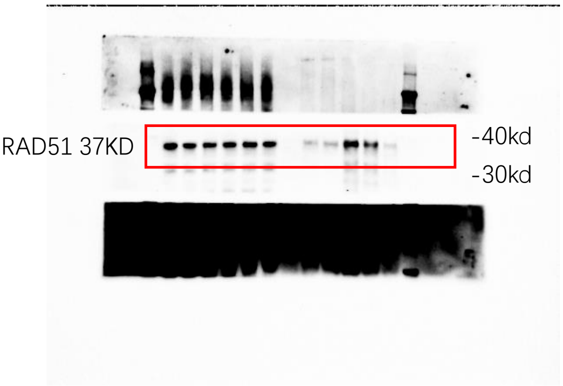
RAD51 37KD
-40kd
-30kd

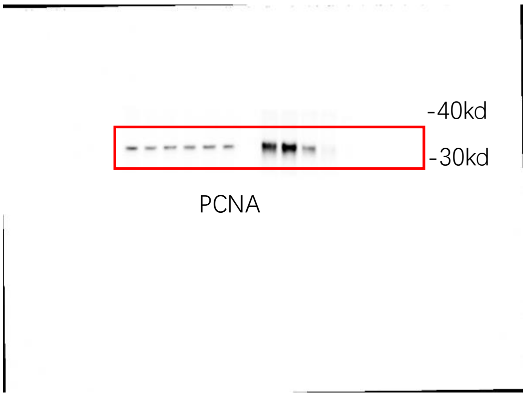
-40kd
-30kd
PCNA

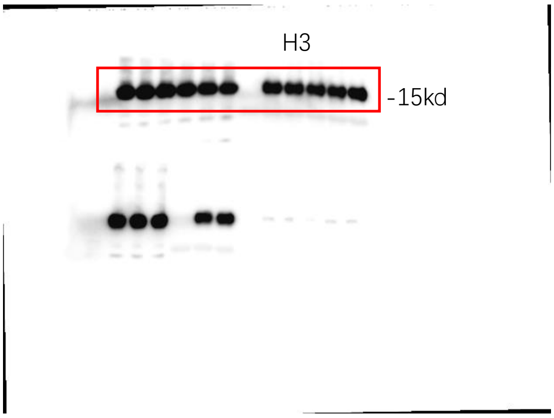

Fig.5a

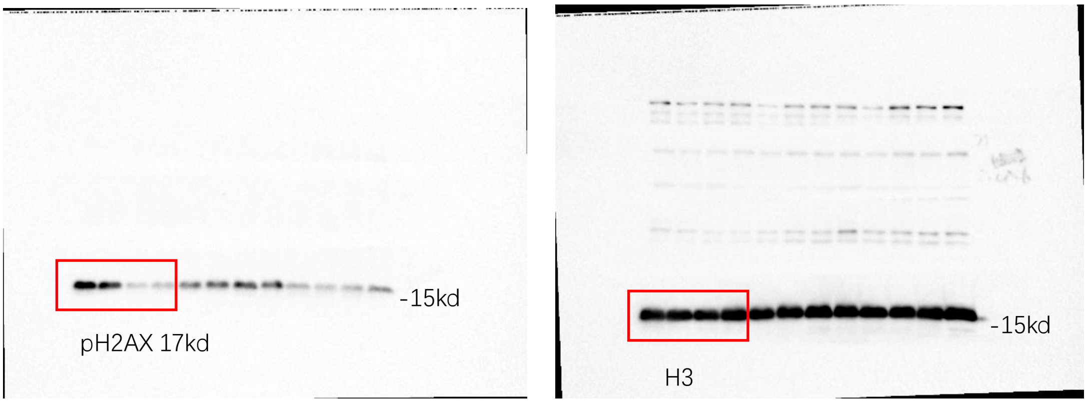

Fig.5b

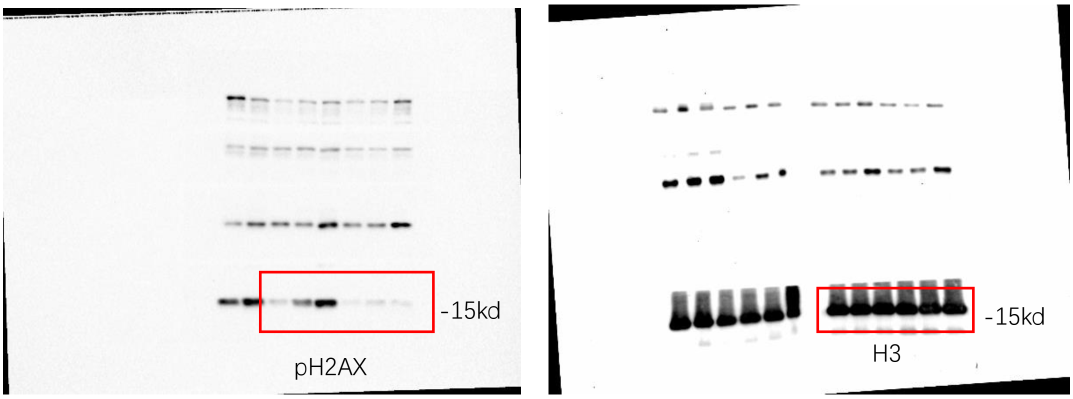

Supplementary Fig. 1a

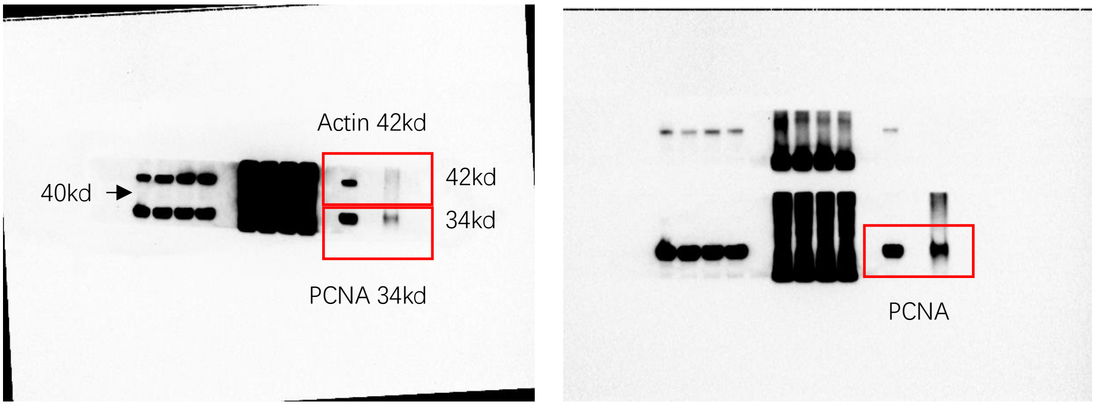

Supplementary Fig.1c

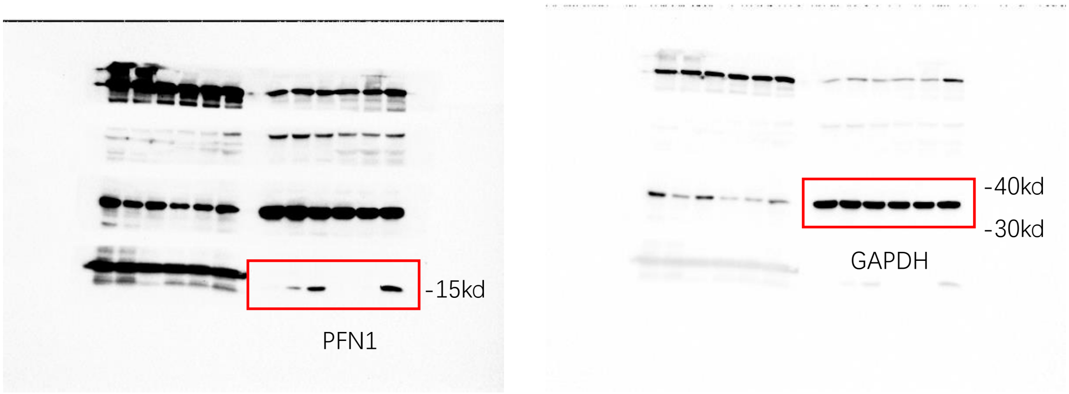

Supplementary Fig. 1e

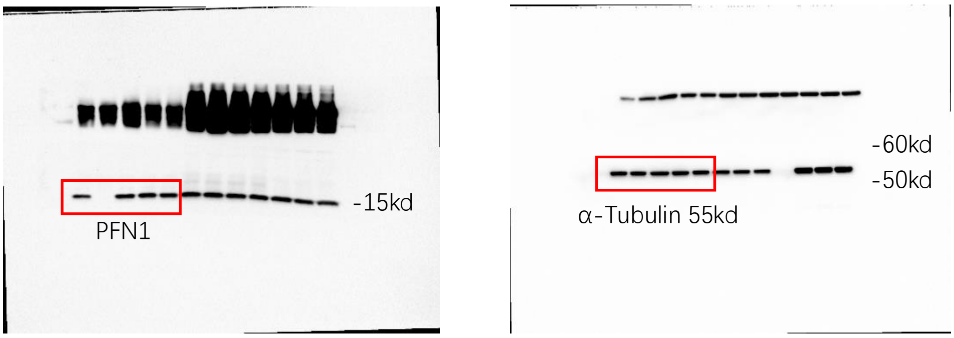

Supplementary Fig.1g

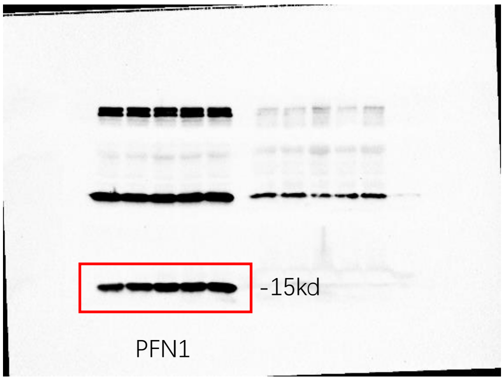

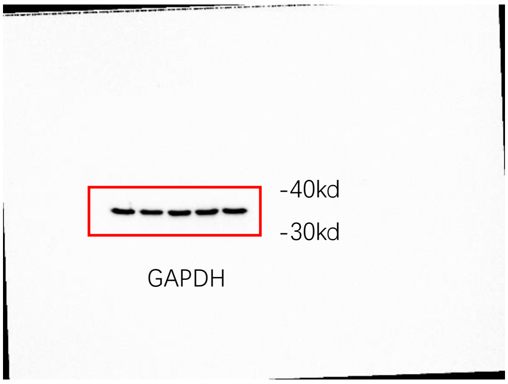

Supplementary Fig.1h

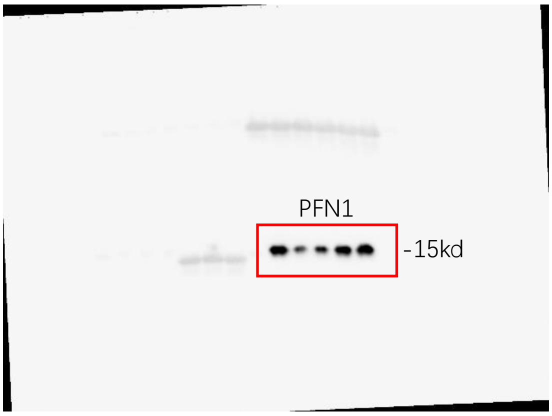

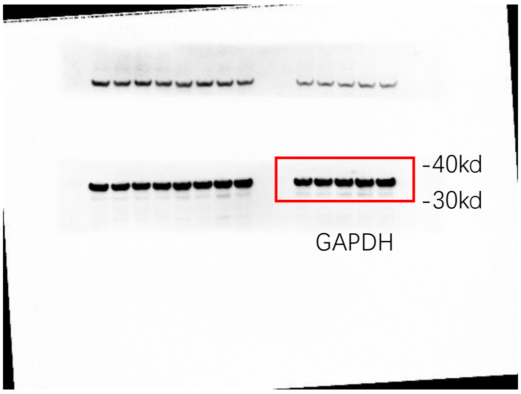

Supplementary Fig. 1j

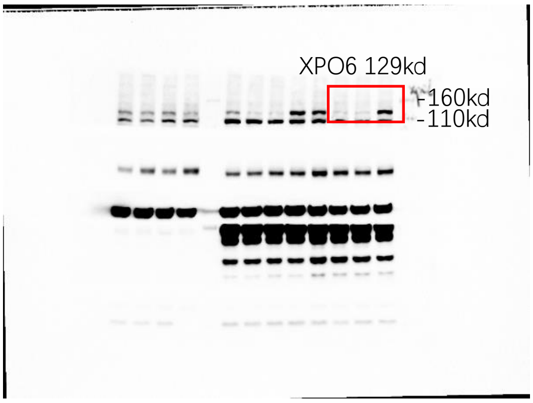

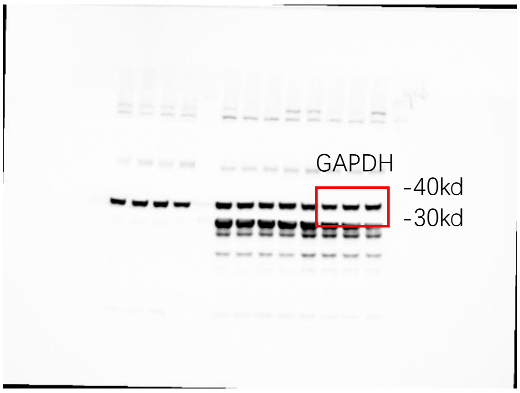

Supplementary Fig.1l

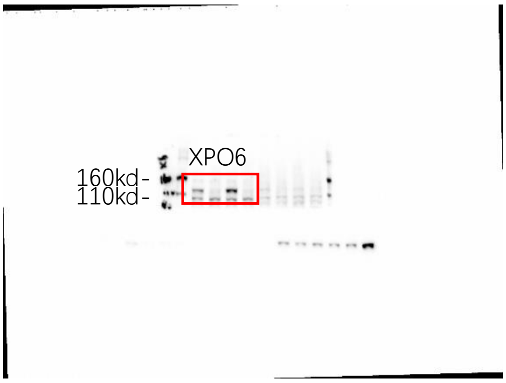

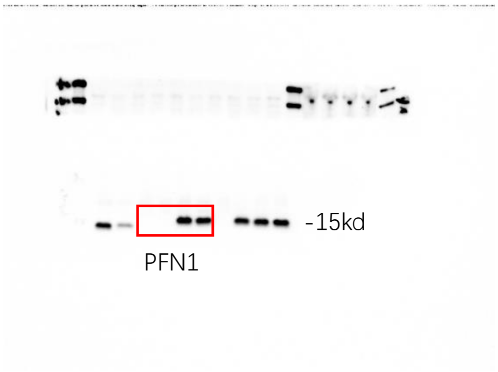

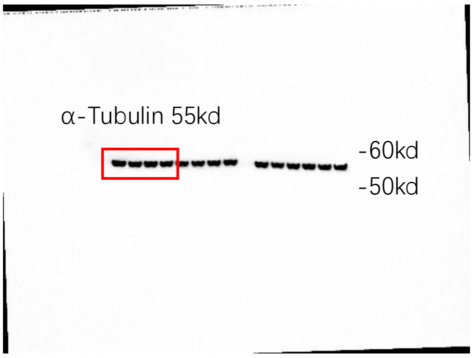

Supplementary Fig.2e

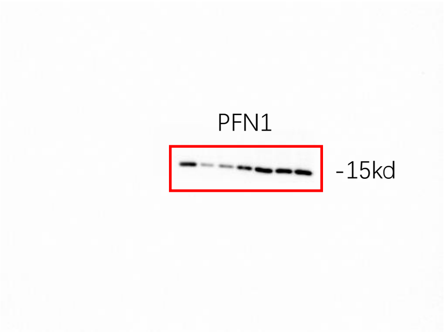

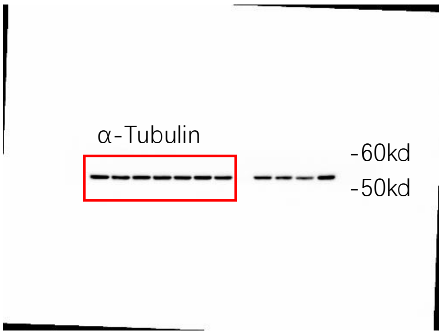

Supplementary Fig.2h

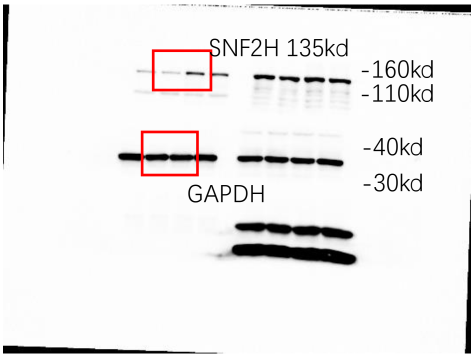

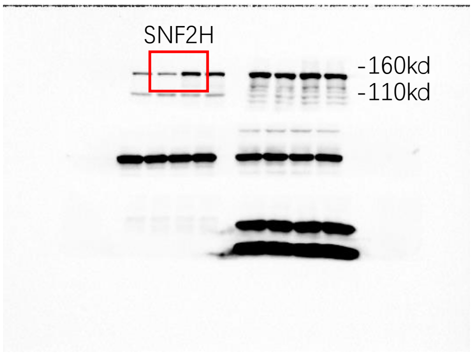

Supplementary Fig.3b

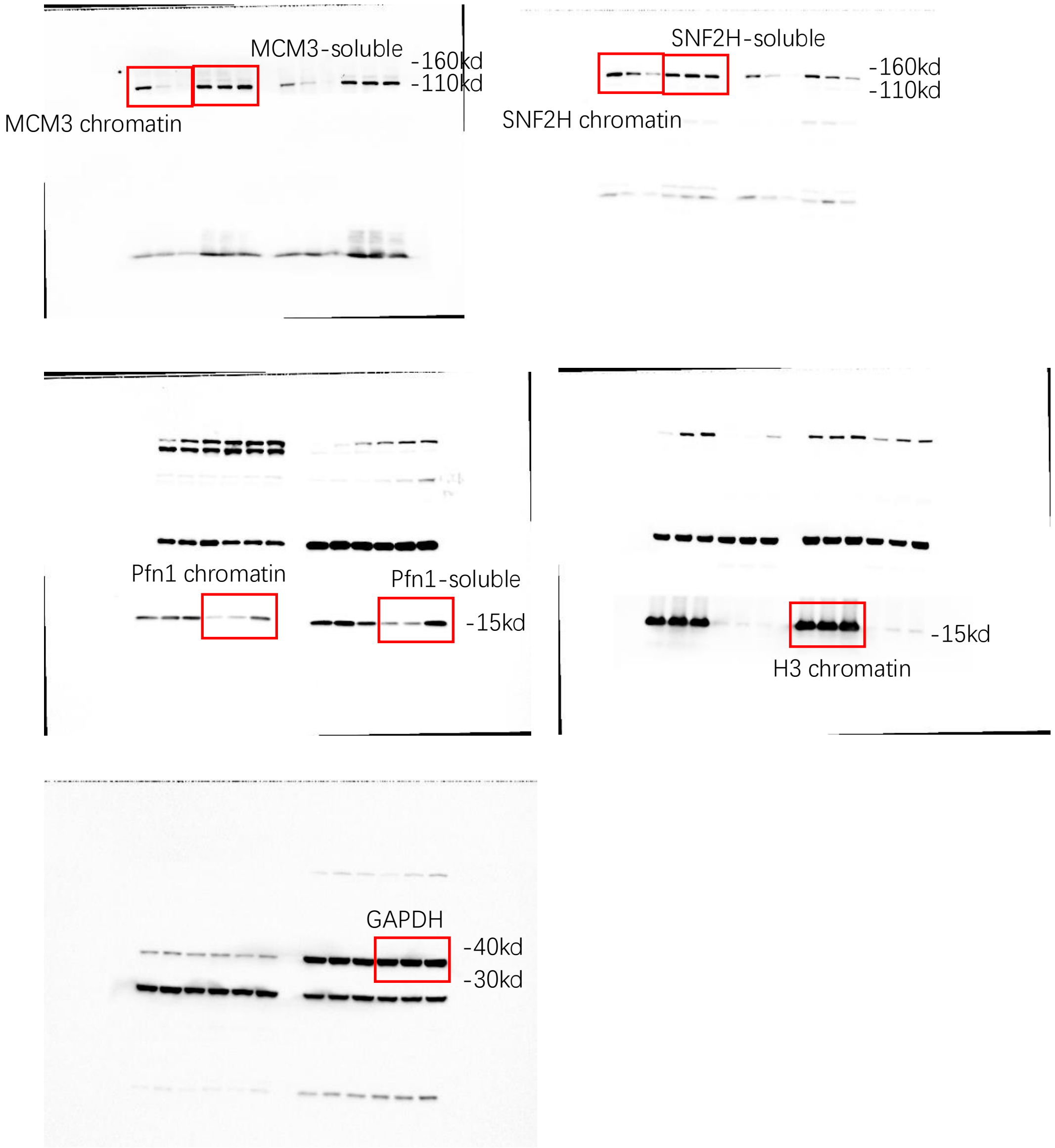

Supplementary Fig.3c

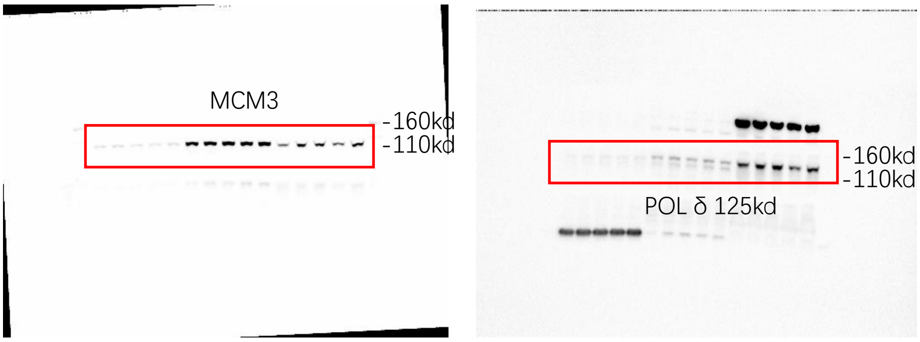

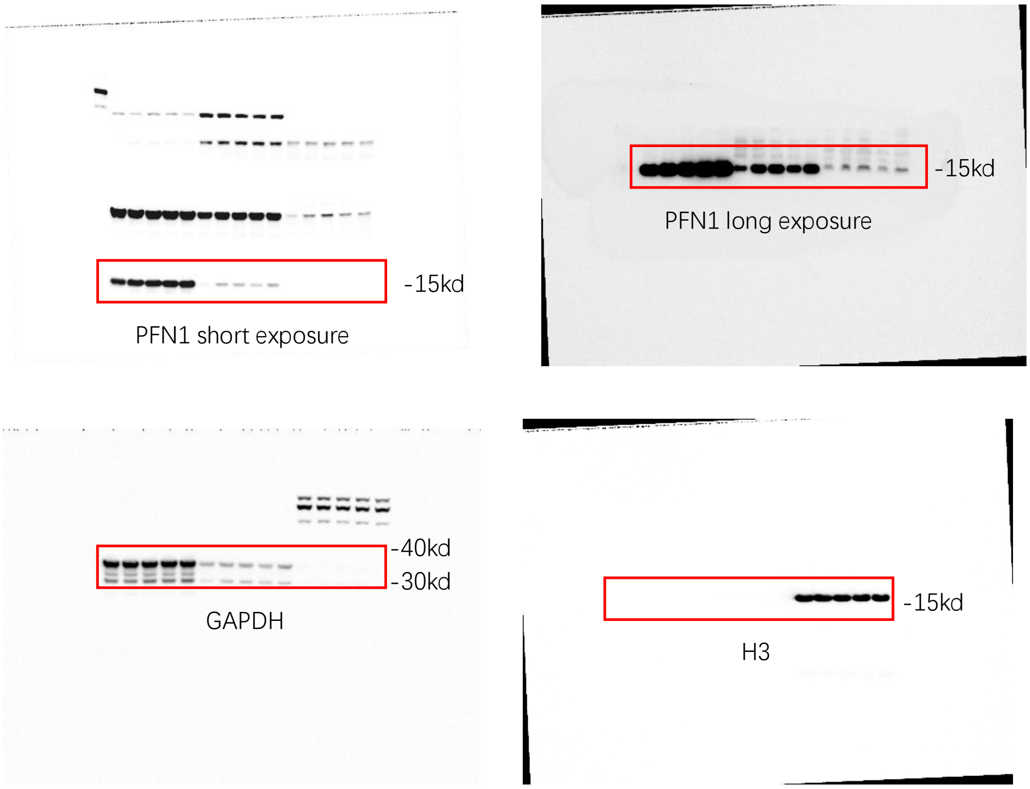

Supplementary Fig.4g

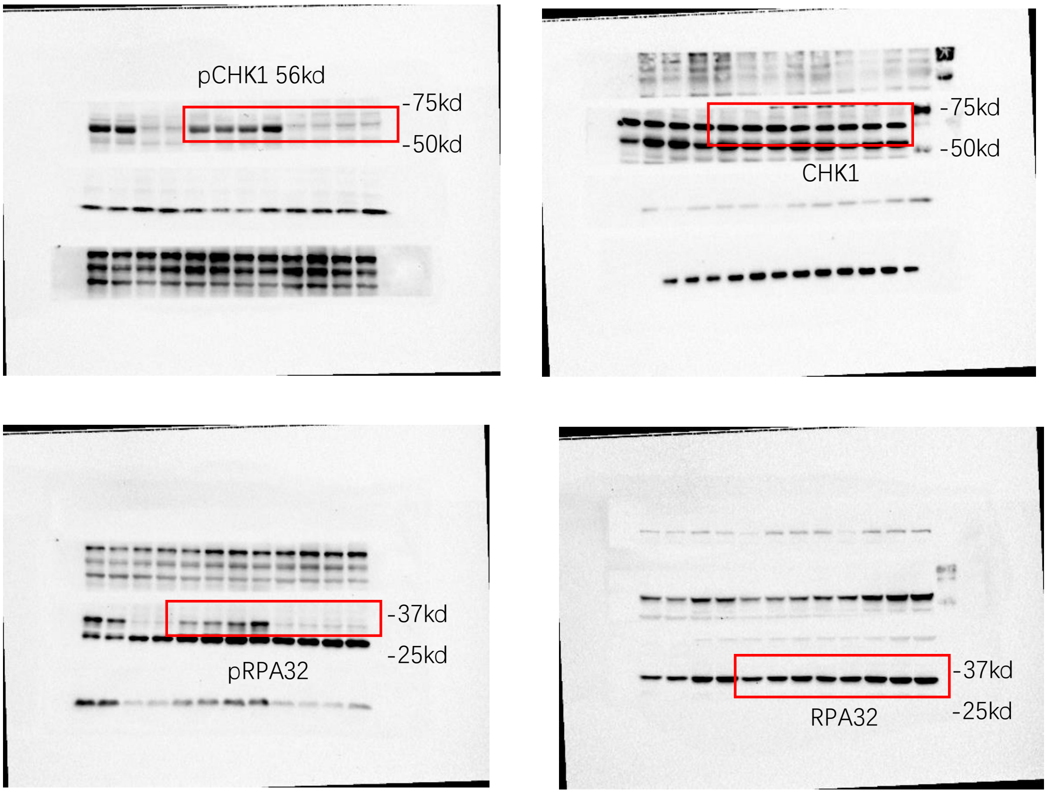

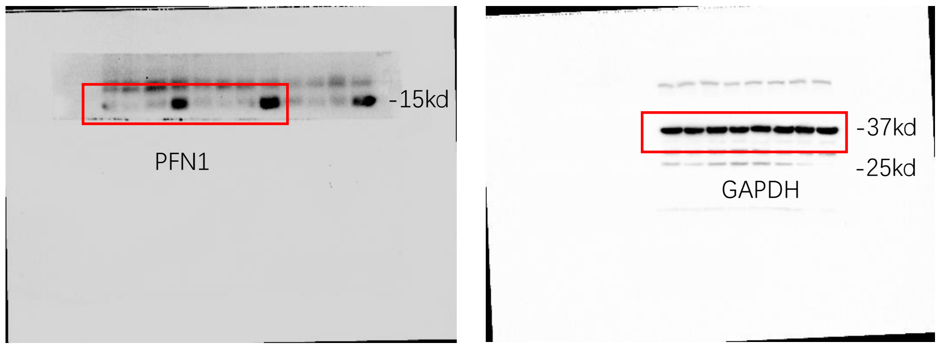

Supplementary Fig.4h

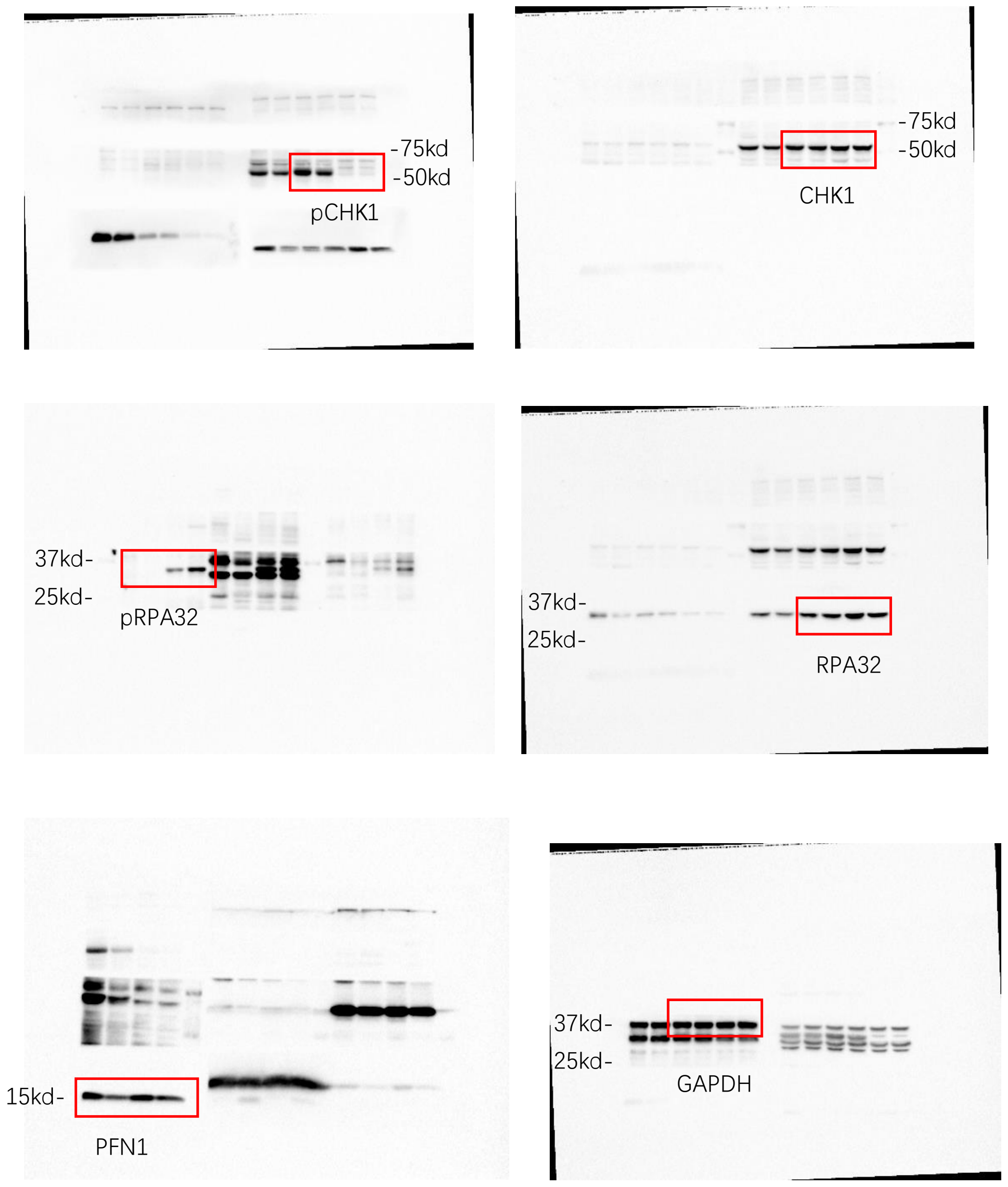

Supplementary Fig.4k

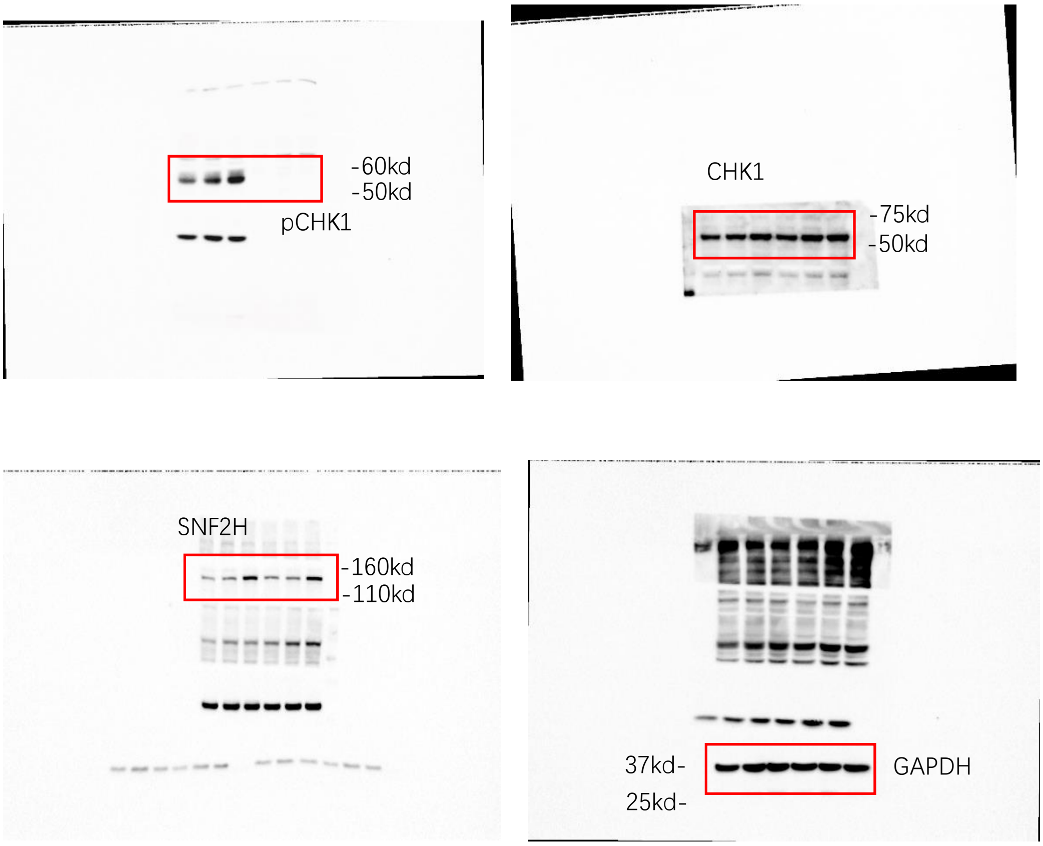

Supplementary Fig.7a

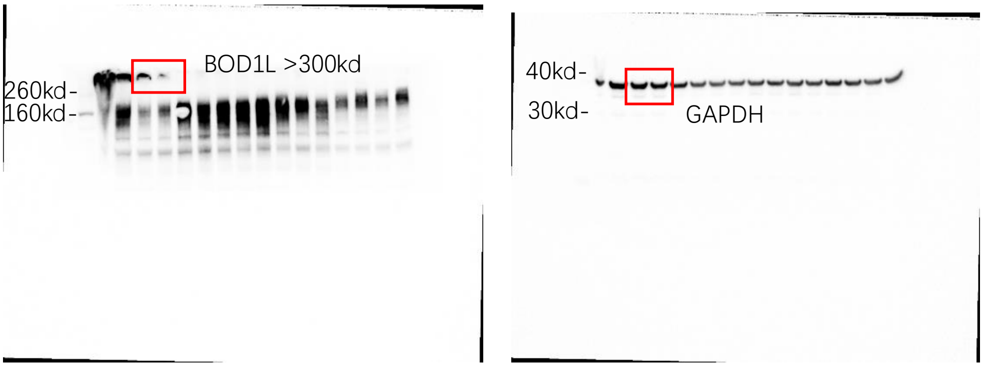

Supplementary Fig.7c-7d

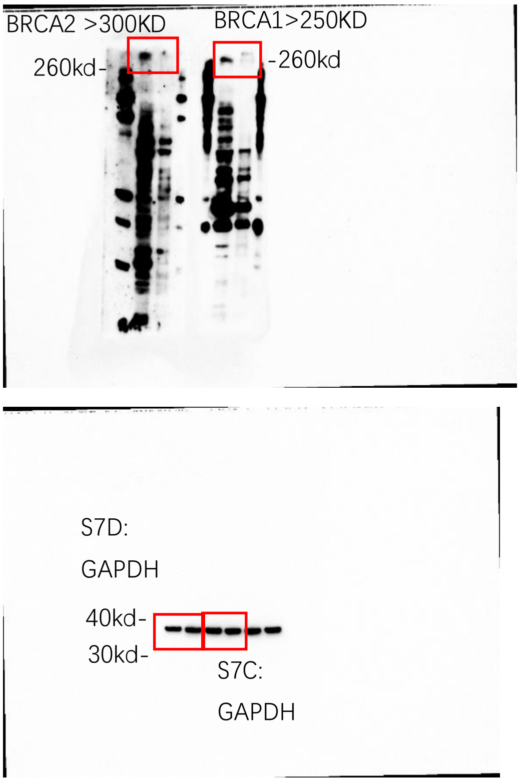

Supplementary Fig.8b

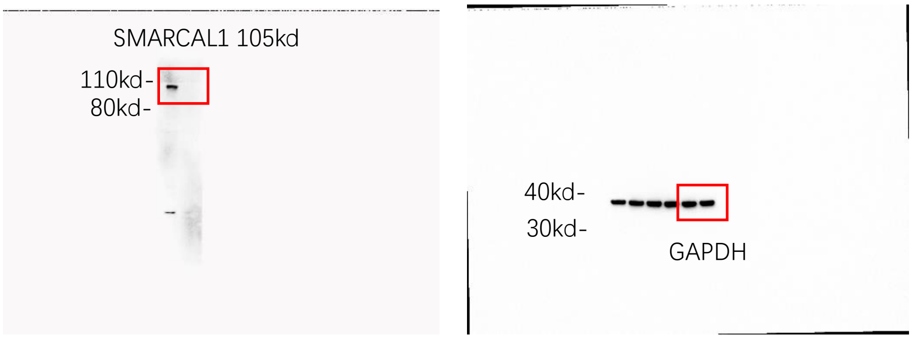

Supplementary Fig.8c

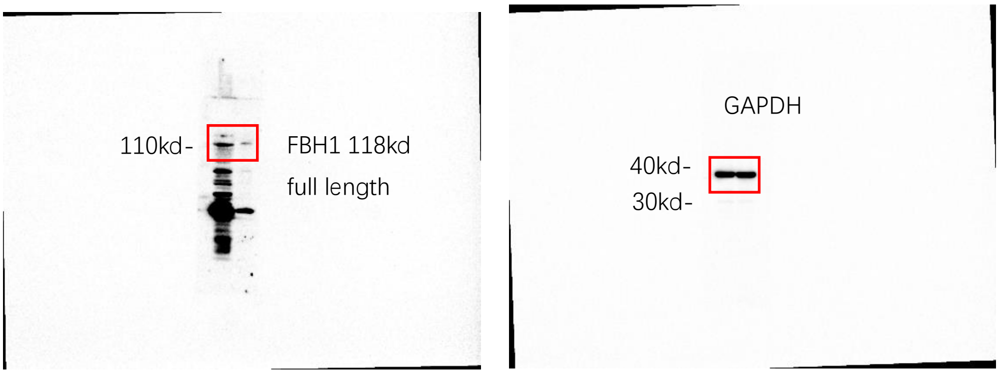

Supplementary Fig.9g

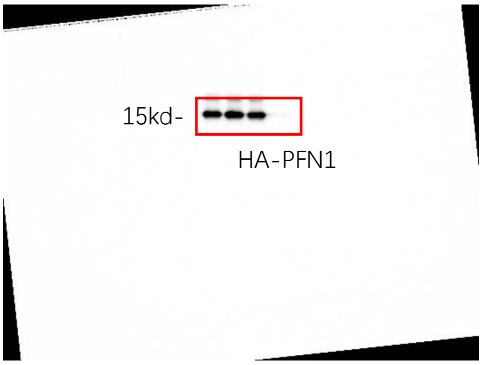

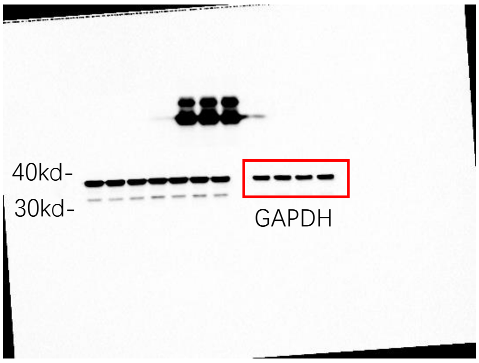

Fig.S9H

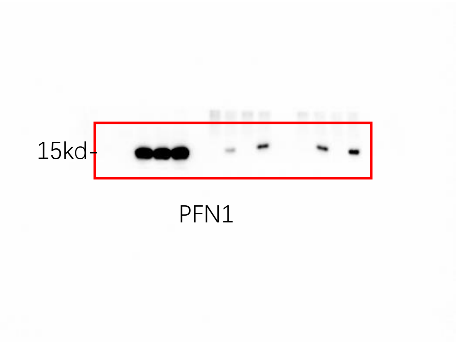

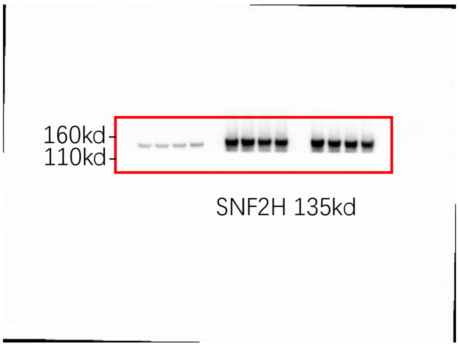

Supplementary Fig.9i

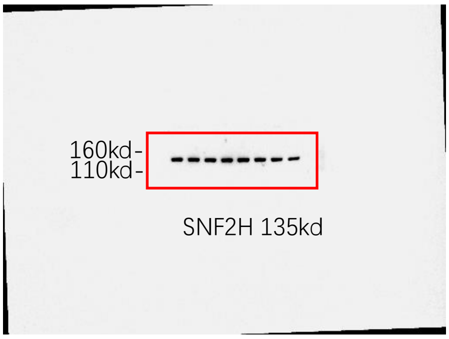

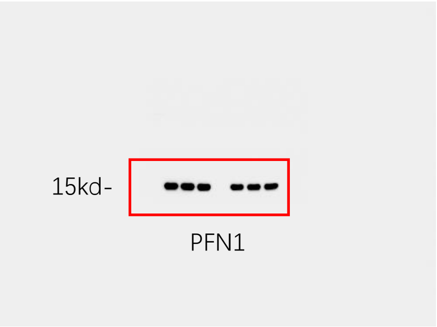

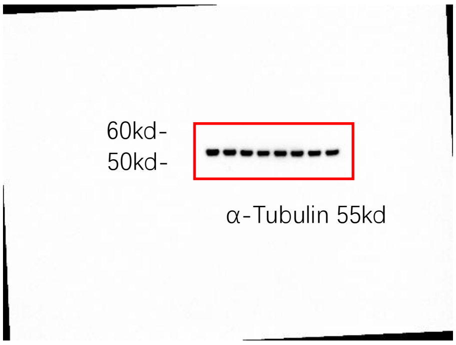

DNA gel and Anti-brdU blots

Supplementary Fig.2 and Fig.9

NO MNase SYBR gel

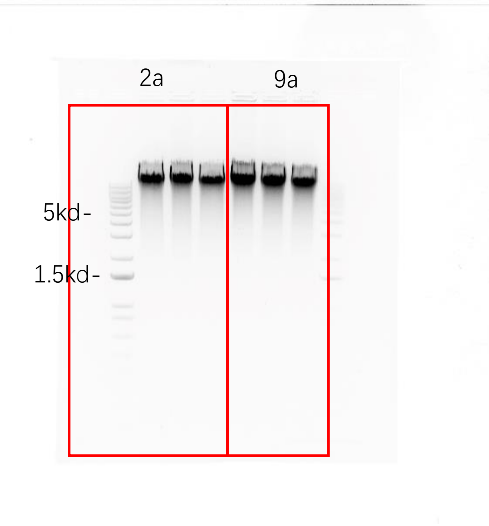

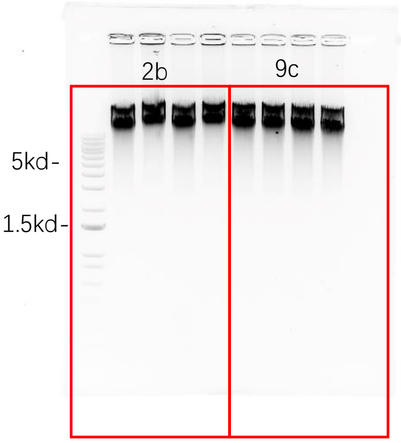

MNase SYBR Gel

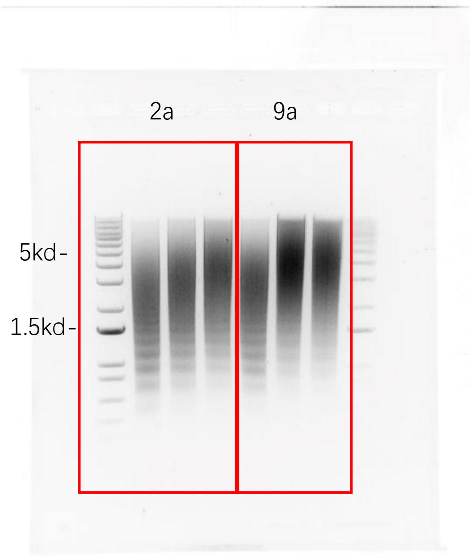

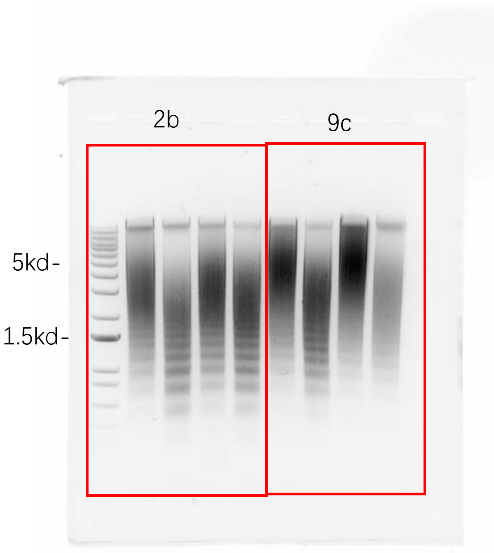

NO MNase Anti-BrdU blots

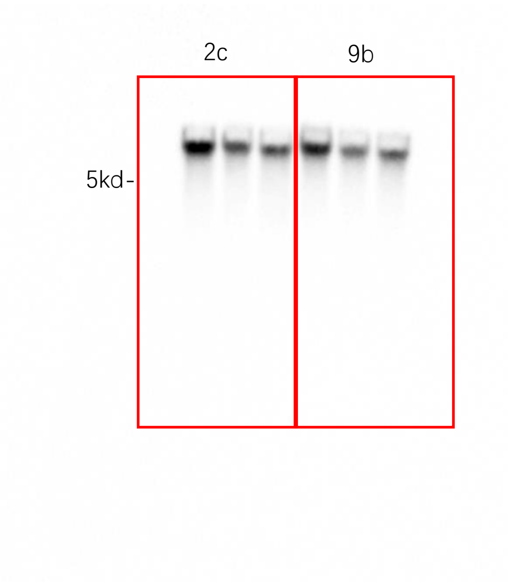

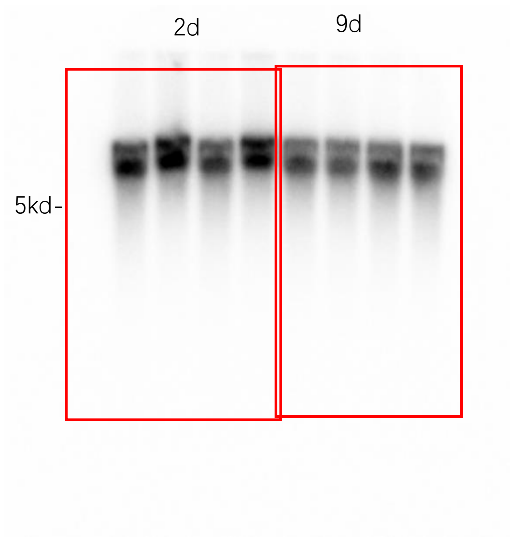

MNase Anti-BrdU blots

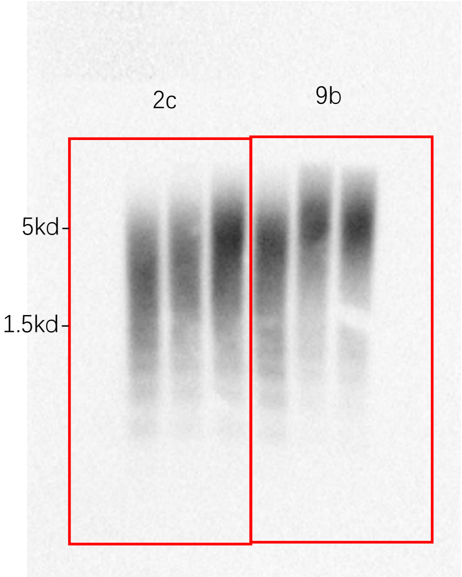

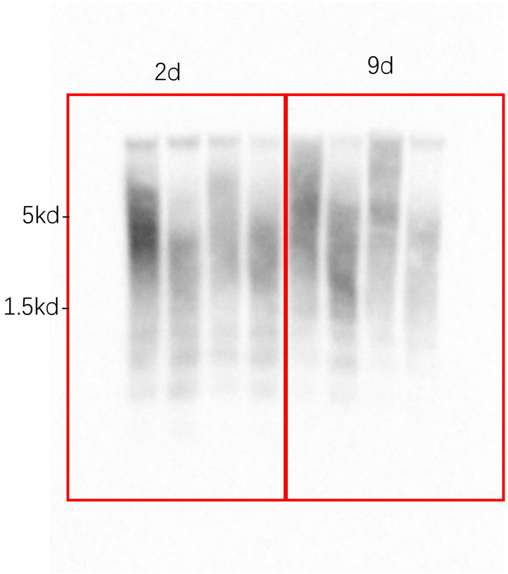

Supplement: Supplementary file 3 — Source Data [file 41467_2022_34310_MOESM3_ESM.zip › source data/Shao_uncropped blots.pdf]
